# Transcriptomic Analysis Identifies New Non-Target Site Glyphosate-Resistance Genes in *Conyza bonariensis*

**DOI:** 10.3390/plants8060157

**Published:** 2019-06-07

**Authors:** Cristiano Piasecki, Yongil Yang, Daiane P. Benemann, Frederico S. Kremer, Vanessa Galli, Reginald J. Millwood, Joanei Cechin, Dirceu Agostinetto, Luciano C. Maia, Leandro Vargas, C. Neal Stewart

**Affiliations:** 1 Department of Crop Protection, Federal University of Pelotas (UFPel), Pelotas 96010-610, Brazil; daiane.benemann@ufpel.edu.br (D.P.B.); jcechin@ufpel.edu.br (J.C.); dirceu_agostinetto@ufpel.edu.br (D.A.); 2 Department of Plant Sciences, University of Tennessee (UTK), Knoxville, TN 37996, USA; yyang98@utk.edu (Y.Y.); rmillwood@utk.edu (R.J.M.); 3 Center for Technological Development, Federal University of Pelotas (UFPel), Pelotas 96010-610, Brazil; frederico.kremer@thrivedatascience.com (F.S.K.); vanessa.galli@ufpel.edu.br (V.G.); 4 Department of Plant Breeding, Federal University of Pelotas (UFPel), Pelotas 96010-610, Brazil; luciano.maia@ufpel.edu.br; 5 Department of Weed Science, Brazilian Agricultural Research Corporation (Embrapa), Passo Fundo 99050-970, Brazil; leandro.vargas@embrapa.br

**Keywords:** hairy fleabane, herbicide resistance, herbicide metabolization, non-target-site resistance (NTSR), RNA-Seq, next-generation sequencing, differential gene expression

## Abstract

*Conyza bonariensis* (hairy fleabane) is one of the most problematic and widespread glyphosate-resistant weeds in the world. This highly competitive weed species significantly interferes with crop growth and substantially decreases crop yield. Despite its agricultural importance, the molecular mechanisms of glyphosate resistance are still unknown. The present RNA-Seq study was performed with the goal of identifying differentially expressed candidate transcripts (genes) related to metabolism-based non-target site glyphosate resistance in *C. bonariensis*. The whole-transcriptome was *de novo* assembled from glyphosate-resistant and -sensitive biotypes of *C. bonariensis* from Southern Brazil. The RNA was extracted from untreated and glyphosate-treated plants at several timepoints up to 288 h after treatment in both biotypes. The transcriptome assembly produced 90,124 contigs with an average length of 777 bp and N50 of 1118 bp. In response to glyphosate treatment, differential gene expression analysis was performed on glyphosate-resistant and -sensitive biotypes. A total of 9622 genes were differentially expressed as a response to glyphosate treatment in both biotypes, 4297 (44.6%) being up- and 5325 (55.4%) down-regulated. The resistant biotype presented 1770 up- and 2333 down-regulated genes while the sensitive biotype had 2335 and 2800 up- and down-regulated genes, respectively. Among them, 974 up- and 1290 down-regulated genes were co-expressed in both biotypes. In the present work, we identified 41 new candidate target genes from five families related to herbicide transport and metabolism: 19 ABC transporters, 10 CYP450s, one glutathione S-transferase (GST), five glycosyltransferases (GT), and six genes related to antioxidant enzyme catalase (CAT), peroxidase (POD), and superoxide dismutase (SOD). The candidate genes may participate in metabolic-based glyphosate resistance via oxidation, conjugation, transport, and degradation, plus antioxidation. One or more of these genes might ‘rescue’ resistant plants from irreversible damage after glyphosate treatment. The 41 target genes we report in the present study may inform further functional genomics studies, including gene editing approaches to elucidate glyphosate-resistance mechanisms in *C. bonariensis*.

## 1. Introduction

Weed interference is among the most significant biotic constraints to crop yield and global food security, causing up to $100 billion in damage annually [1,2,3]. Weed species with widespread occurrence and highly competitive capacity with crops that are difficult to manage are particularly damaging to agricultural security. *Conyza bonariensis* (L.) Cronq., commonly called hairy fleabane, is one of the most threatening and difficult weed species to manage around the world [4,5]. This Asteraceae species is native to the Americas and has a cosmopolitan distribution [4]. Some of its weedy traits include high production and dispersion of seeds, phenotypic plasticity, and tolerance to unfavorable environmental conditions [4,6]. Further, the evolved glyphosate resistance (GR) is a critical factor that complicates controlling weeds in the field [7]. 

The first report of glyphosate-resistant *C. bonariensis* in Brazil occurred in 2005 [8]. Since then, the evolution of resistance to glyphosate in this species has become a significant concern around the world, and to date, there have been 13 GR reports in several crops and countries [9]. *Conyza* spp. have become one of the world’s worst weeds because of their propensity to evolve strong herbicide resistance [7] in both cropland and fallow land [10,11,12]. 

Glyphosate is a valuable herbicide because of its high efficacy to a broad spectrum of annual and perennial weeds, relatively low cost, and low negative impact on the environment when compared to other herbicides [13,14]. However, the intensive and widespread use of glyphosate has been a factor in evolved resistance [13,15]. The glyphosate target site is the chloroplast enzyme 5-enolpyruvylshikimate-3-phosphate synthase (EPSPS), which catalyzes the reaction converting phosphoenolpyruvate (PEP) and 3-phospho-shikimate into phosphate and 5-enolpyruvylshikimate-3-phosphate [14,16]. Glyphosate interrupts the shikimic acid pathway and disrupts aromatic amino acid biosynthesis, causing alterations in the metabolic stoichiometry of carbon intermediates. The massive physiological disruption in glyphosate-sensitive (GS) plants leads to their death in a matter of days [17]. However, weeds have evolved multiple mechanisms to tolerate glyphosate treatment, reduce its damage, and facilitate weeds’ recovery and fitness after exposure.

GR mechanisms can be grouped into two large categories: target site (TS) resistance, i.e., caused by changes in the EPSPS gene and shikimic acid metabolism, and non-target site resistance mechanism (NTSR), which encompasses, essentially, all mechanisms not involving EPSPS-related alterations [18,19,20]. To-date, TS resistance has not been observed in *C. bonariensis*; thus, one or more NTSR mechanisms are to blame [6,16] but have not been elucidated. The best physiological evidence indicates that NTSR in *C. bonariensis* is caused by subcellular glyphosate sequestration, likely to the vacuole, which prevents glyphosate from being translocated to tissues that have not been sprayed [6,16,21,22]. However, the molecular mechanisms are unknown for any GR weed.

Transcriptomic studies have been performed at 24 h and 48 h after glyphosate treatment to understand the genomic basis of glyphosate NTSR in *Conyza canadensis* (United States) [23] and *C. bonariensis* (Australia) [16]. However, the specific mechanism of glyphosate NTSR has not yet been elucidated [6,16,24]. After herbicide treatment, plant damage begins within the first 3 to 8 h [25]. NTSR mechanisms can typically rescue resistant plants before irreversible cellular damage has occurred and must have sufficient persistence to outlast glyphosate activity [25,26,27]. Moreover, stress-responsive genes have circadian regulation, and transcriptome sampling during an off-peak regulation period may thus lead to an incomplete representation of the plant molecular response to herbicide [26,27]. 

One hypothesis for why published transcriptomic analyses have failed to identify NTSR genes responsible for GR is that RNA has been isolated too soon after glyphosate treatment, which missed gene regulatory changes related to key metabolic steps in GR weeds. Our previous studies using the *C. bonariensis* biotypes from Brazil, which are also used in the present study, have shown that shikimic acid accumulation in GR hairy fleabane is transient, reaching its peak at 96 h after glyphosate treatment and then declining. Approximately 288 h after treatment, shikimic acid concentrations of GR plants are equivalent to those of untreated plants [28]. Another study reported similar transient shikimic acid accumulation in *C. canadensis* [29]. Thus, shikimic acid content may be an important indicator of weedy plant response to glyphosate treatment. The strategy of assaying gene expression relative to shikimic acid content, which is the first indicative of the EPSPS inhibition after glyphosate treatment, has the potential to help us to better understand the underlying genomics of NTSR biotypes vis-à-vis GR biotypes vs. GS biotypes. This paper describes the results of the *de novo* assembly of the transcriptome (RNA-Seq) of GR regarding the GS *C. bonariensis* over a long period (up to 288 h after glyphosate spray), and then the identification of candidate NTSR genes based on differential gene expression data.

## 2. Results

The present study aimed to study the molecular changes of *C. bonariensis* in response to glyphosate treatment in the long term after herbicide treatment. The timepoints of RNA extraction to RNA sequencing were determined based on shikimic acid content in the GR biotype from 24 h to 288 h after treatment. In the last timepoint, the shikimic acid content on treated GR plants did not differ regarding to untreated plants. Thus, the gene expression changes were evaluated in GR glyphosate treated and untreated plants in contrast to GS with the same treatments.

### 2.1. Illumina Sequencing and De Novo Assembly

The twelve cDNA libraries sequenced from *C. bonariensis* leaves produced a high-quality assembled dataset (Table S1). The Phred scores of the *de novo* assembled data were ≥Q30 level (error probability 0.1%) for more than 90% of all raw reads. Transcriptome assembling produced 203,054 (>200 bp) transcripts and 90,124 contigs at the “gene” level (N50 of 1118 bp and the average length of 777 bp) (Table S1). The sequence distribution of transcripts length threshold varied from 0 to 3000 bp (Figure S1). In this way, the large scale and high-quality results provided in the present study will serve as molecular reference for further *C. bonariensis* studies. 

### 2.2. Functional Annotation of Assembled Contigs

The BLASTx results of sequences indicated that 68.1% of the annotated contigs (90,124) had hits with nucleotide sequences from the NCBI database (Figure S2). Most of the annotated nucleotide sequences of *C. bonariensis* transcriptome corresponded to *Helianthus annuus* (36.9%) and *Cynara cardunculus* var. *scolymus* (18.8%). Further, the contig identity to other plant species was 12.4%, and the number of novel genes without interspecies identity was 31.9% (Figure S3).

The functions of *C. bonariensis* contigs were classified according to gene ontology assignments. A total of 11,877 contigs (13.2%) were attributed to at least one gene ontology term and classified in 30 functional categories using the complete set of GO terms in three main categories: molecular function (n = 3188, 26.9%), biological process (n = 7491, 63.1%), and cellular component (n = 1198, 10.1%) (Figure S4). The largest proportion of genes was represented by a cellular (3.7%) and metabolic process (3.2%) in the biological process; molecular function (4.7%) and binding (3.5%) in molecular function; and a cellular component (4.7%) and cell organelle (4.2%) in cellular component (Figure S4). 

The high number of novel genes (31.9%) provided in the present RNA sequencing results shows the importance of characterizing genomes of weeds and also serves to enable follow-on molecular studies in *C. bonariensis* and other weedy species. Further genome sequencing studies in *Conyza* spp. will be helpful to completely characterize the novel genes presented here. Further, the gene ontology indicates that the biological process represented the highest proportion of annotated genes regarding molecular function and cellular component.

### 2.3. Differentially Expressed Genes

**Figure 1:**
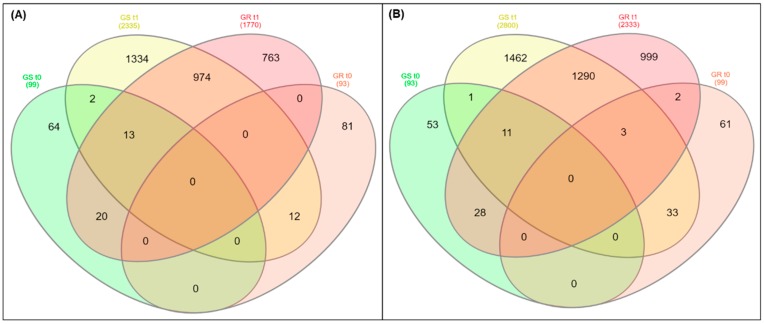
The differential expression results presented in a Venn diagram from transcriptome analysis in glyphosate-resistant (GR) and -sensitive (GS) biotypes of *Conyza bonariensis* in response to glyphosate treatment. (**A**) Up-regulated; (**B**) down-regulated. t0: without glyphosate treatment; t1: with glyphosate treatment—RNA was obtained from plants collected at several timepoints up to 288 h after treatment and pooled. Genes were filtered according to relative expression (count-number), *p-*value and false discovery rate (FDR) set at ≤0.001.

The total of differentially expressed genes (DEGs) (9622 “genes”; 39.9% of total) was found in response to glyphosate treatment in both biotypes: 4297 (44.6%) genes were up-regulated and 5325 (55.4%) were down-regulated (Figure 1 and Figure S5). Among the DEGs, 1770 and 2333 genes were up- and down-regulated in GR plants, while 2335 and 2800 genes were up- and down-regulated in GS plants, respectively. A total of 974 up- and 1290 down-regulated genes were co-expressed in both biotypes as a response to glyphosate treatment (Figure 1, Figure S5). In GR and GS biotypes without glyphosate treatment (t0), there were around 100 genes each in up- and down-regulation, with no apparent co-expression (Figure 1).

**Figure 2:**
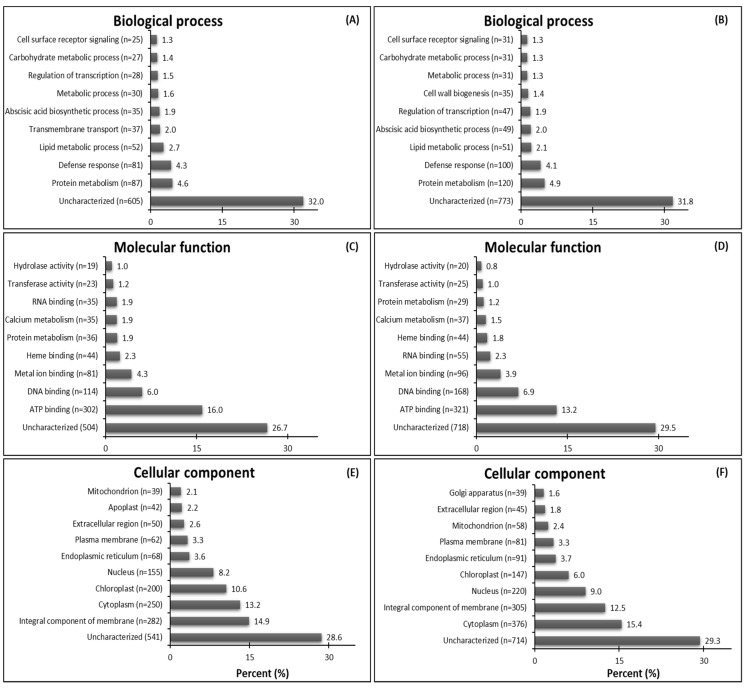
Summary of top ten gene ontology (GO) terms identified as up-regulated genes (DEGs-up) in glyphosate-resistant and -sensitive *C. bonariensis* biotypes. Annotated sequences were classified into the biological process, molecular function, and cellular component. (**A**,**C**,**E**) GR—up-regulated DEGs, n = 1770; (**B**,**D**,**F**) GS—up-regulated DEGs, n = 2335.

The most abundant GO terms for up-regulated genes were biological process—protein metabolism (87 DEGs from GR plants and 120 DEGs from GS plants) (Figure 2A,B); molecular function—ATP binding (302 DEGs from GR plants and 321 DEGs from GS plants) (Figure 2C,D); cellular component—integral component of membrane (282 DEGs from GR plants) and cytoplasm (376 DEGs from GS plants) (Figure 2E,F). It is interesting to note that GR plants have 45.9% more up-regulated genes in transmembrane transport than GS (37 vs. 17). However, in the GR biotype, this process was classified as the fifth-most prominent and was not included among the top ten up-regulated genes in GS (Figure 2A,B). On the other hand, the GS biotype had 126 (50%) more up-regulated genes than GR (376 vs. 250) in processes located in the cytoplasm (cellular component) (Figure 2E,F). Further, the resistant biotype had 53 (36%) more up-regulated genes that are chloroplast localized (200 vs. 147) than in GS plants (Figure 2E,F).

**Figure 3:**
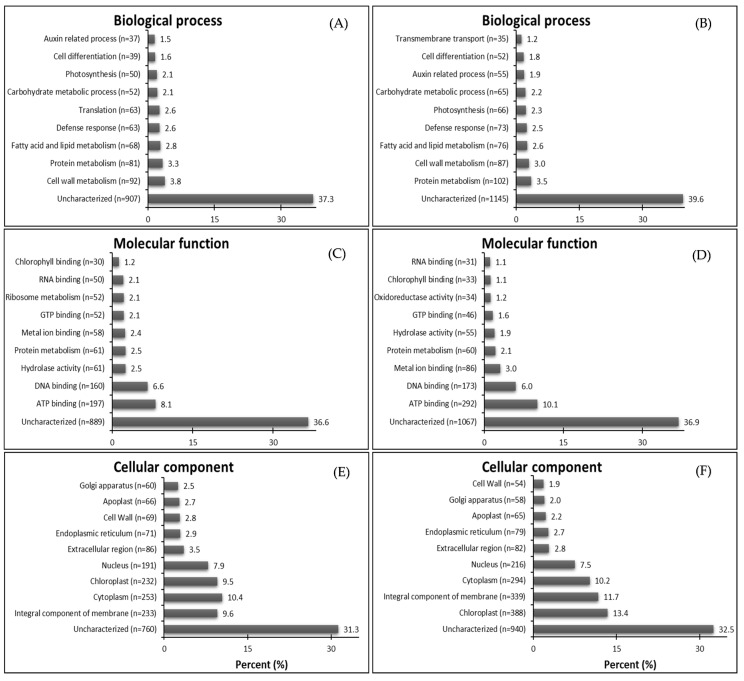
Summary of top ten gene ontology (GO) terms identified as down-regulated genes (DEGs-down) in glyphosate-resistant and -sensitive *C. bonariensis* biotypes. Annotated sequences were classified into the biological process, molecular function, and cellular component. (**A**,**C**,**E**) GR—down-regulated DEGs, n = 2333; (**B**,**D**,**F**) GS—down-regulated DEGs, n = 2800.

The attributed GO functions for down-regulated genes show the highest proportions of characterized genes respectively in GR and GS related to biological process—cell wall metabolism (3.8%) and protein metabolism (3.5%) (Figure 3A,B); molecular function—ATP binding for both biotypes (8.1% and 10.1%) (Figure 3C,D); and cellular component—integral component of membrane (9,6%) and chloroplast (13.4%) (Figure 3E,F). In the biological process, 63 genes related to translation were down-regulated in GR while just 16 in GS, which do not figure in the top ten in the sensitive biotype. Further, the transmembrane transport-related contigs were not among the top ten down-regulated DEGs in GR but were so in GS (Figure 3A,B). In molecular function, there were 52 down-regulated genes related to ribosome metabolism in GR while just one in GS (Figure 3C,D). Further, GR presented 40% and 31% less down-regulated genes related to chloroplast and integral component of the membrane than GS (Figure 3E,F).

The high number of DEGs represents the high molecular changes that glyphosate action causes in the *C. bonariensis* transcriptome. Further, the differences on expressed genes according to each category show differences between GR and GS biotypes and might indicate the processes related with resistance to glyphosate.

### 2.4. RNA-Seq Validation by qRT-PCR Analysis

**Figure 4:**
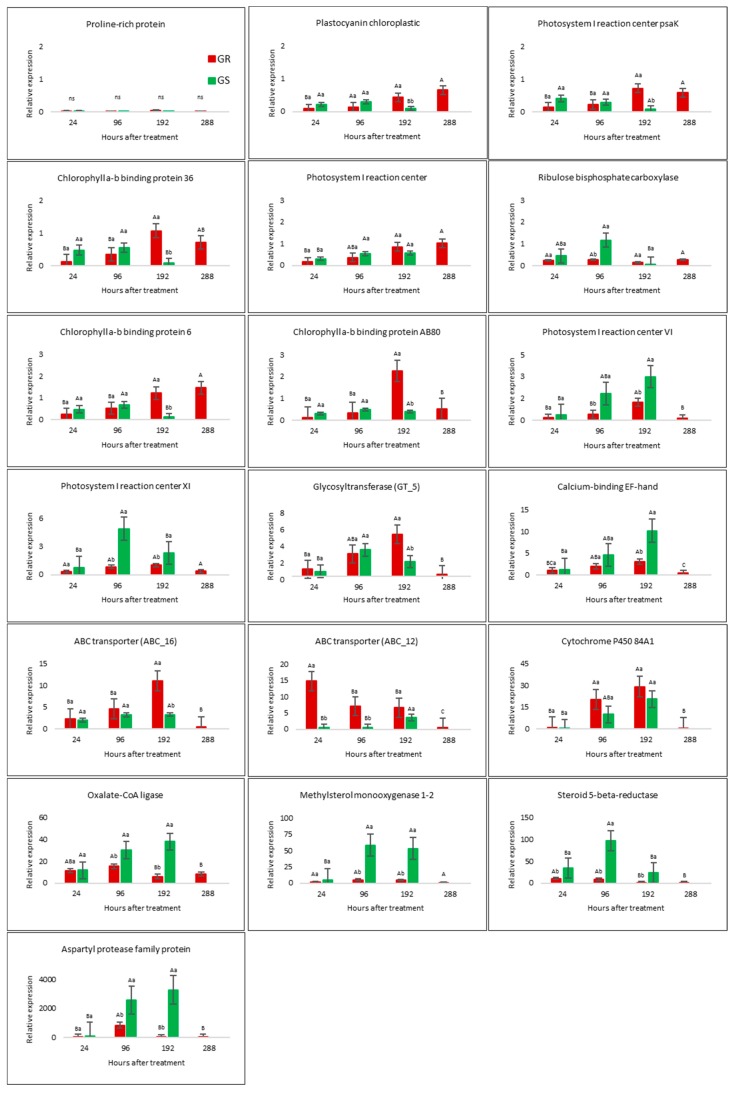
Relative expression levels of 19 selected genes in leaves of *C. bonariensis* glyphosate-resistant (GR—red bars) and -sensitive (GS—green bars) biotypes in a time-course experiment after glyphosate treatment relative to an internal control hexosyltransferase gene expression in the untreated plants using real-time RT-PCR (2^−^^△△Ct^). Results were compared by contrasts (*p* ≤ 0.05), and upper-case letters indicate comparisons of each biotype in different times and lower-case letters between biotypes in each respective time.

The average expression for 19 contigs with relative expression performed in a time course indicates an amplitude of (log fold change—logFC) from 0.01 to 1990, while the transcriptome results for the same contigs ranged from logFC 0.1 to 3625 (Figure 4 and Figure S6). The transcriptome dataset and qRT-PCR presented a significant correlation (r) of 0.9 for transcriptional results of GR and GS biotypes (Figure S6). On the other hand, the results of the time course qRT-PCR experiments show that gene expression response to glyphosate treatment varies according to gene-related function, to biotype, and to time after herbicide exposure (Figure 4). In general, the results of the evaluated genes in a time course experiment suggest that the highest expression levels occur between 96 and 192 h after treatment (HAT) (Figure 4). The differences of gene expression in time might be a very good reference for further studies involving glyphosate resistance; between 96 and 192 HAT is the best time-window to capture molecular responses to glyphosate treatment in *C. bonariensis.*

### 2.5. EPSPS Sequence Analysis

The BLASTn analysis identified, with an E-value threshold of zero and score bits >600, one single copy of each the three EPSPS sequences assembled in each one of the individual *C. bonariensis* transcriptomes of GR and GS. There was no amino-acid substitution at Thr 102 and Pro 106 codons from EPSPS sequences alignment (Figure S7A–C). On the other hand, the basal EPSPS transcription varied among the three copies but did not between biotypes. In general, EPSPS2 presented the lowest level of expression among the three copies. In response to glyphosate treatment, the EPSPS2 and EPSPS3 had increased transcription; however, there were no differences between biotypes. EPSPS1 had low variation in expression (Figure S7D). These results indicate that the glyphosate-resistance process in GR plants is due to a NTSR.

### 2.6. Candidate NTSR Genes Related to Putative Functions of Glyphosate Transport and Metabolism

We identified a total of 41 candidates differentially expressed genes associated to the herbicide metabolism phases of oxidation (CYP450), conjugation (GST and GT), transport (ABC transporters), and protection/compensation (antioxidant enzyme—CAT, POD, and SOD) [20,30]. Two ABC transporters and one GT target gene were chosen for validation by qRT-PCR. From these results, the ABC transporters’ transcript abundance varied between biotypes in response to glyphosate treatment and in time after herbicide exposure. The expression of ABC_12 showed the highest expression in GR rather than GS at 24 h after treatment (HAT) (fold change 29.6), whereas ABC_16 at 192 HAT (fold change 3.4). The GT_5 highest expression in GR about GS occurred at 192 HAT with a fold change of 2.8 (Figure 4). Further, eight genes had their highest expression in GS plants, with a more significant increase in expression between 96 and 192 HAT. Among these eight genes, we highlighted the steroid 5-beta-reductase (fold change 11.1—96 HAT) and aspartyl protease (fold change 281.5—192 HAT) (Figure 4).

We identified two copies of each ABC transporters M10 and M11 gene (M10_g1 and M10_g2; M11_g1 and M11_g2) in the *C. bonariensis* transcriptome. Except for M10_g2, all other genes had increased transcript abundance from glyphosate treated plants. The GS biotype presented higher M10 and M11 expression levels than the resistant biotype (Figure S8). Because of that, M10 and M11 genes were not considered glyphosate-resistance candidate genes in the present work.

**Figure 5:**
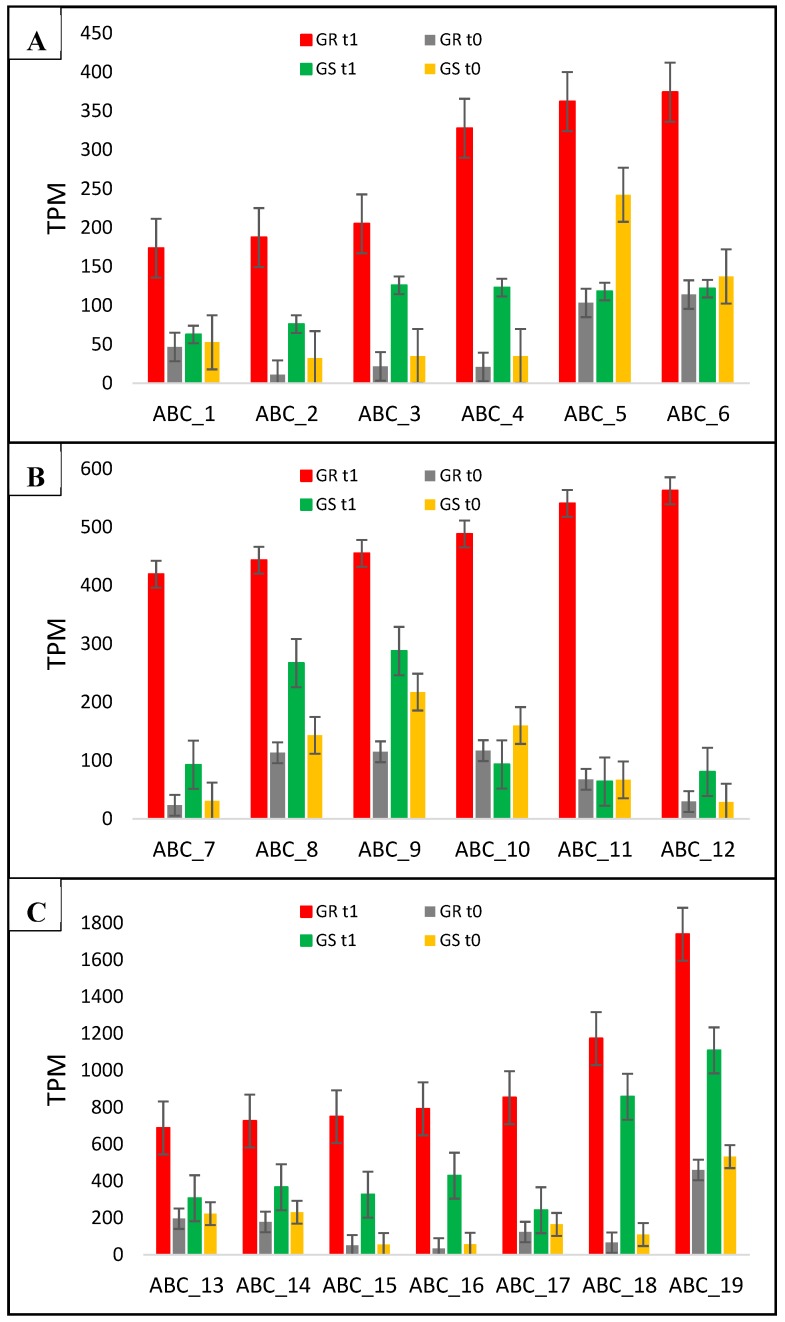
*C. bonariensis* transcriptome expression analysis (transcript reads per million mapped reads—TPM) of the 19 ABC transporters genes (1 to 19). GR: glyphosate-resistant biotype; GS: glyphosate-sensitive biotype. t0: without glyphosate treatment; t1: with glyphosate treatment—RNA was obtained from plants collected at several timepoints up to 288 h after treatment and pooled. Intervals indicate the standard error. In ABC_12 (ABC transporter C family member 14) and ABC_14 (ABC transporter C family member 9), the qRT-PCR analysis was performed (see Figure 4). The phylogenetic tree of these genes is presented in Figure S10.

Here, we report 19 new ABC transporter candidate genes associated with glyphosate resistance in *C. bonariensis*. In the differential expression analysis, these genes had a higher response to glyphosate treatment in GR than in GS with different levels of transcripts abundance (Table 1, Figure 5). Fourteen ABC transporter genes (ABC_2-4, ABC_7-9, ABC_12-19) responded to treatment in both biotypes, but with higher transcription in the resistant biotype. However, two genes (ABC_5 and ABC_10) appeared to have reduced expression in GS plants but increased expression in the GR biotype; and three genes (ABC_1, ABC_6, and ABC_11) had increased transcription only in GR plants with a fold change of 3.1, 3.7, and 8, respectively, by comparing GR- and GS treated biotypes (Figure 5, Table 1). In general, the ABC_2, ABC_5, ABC_7, and ABC_10-12 genes presented the highest expression ratio in the resistant biotype, which varied from 6- to 8-fold (Table 1).

**Figure 6:**
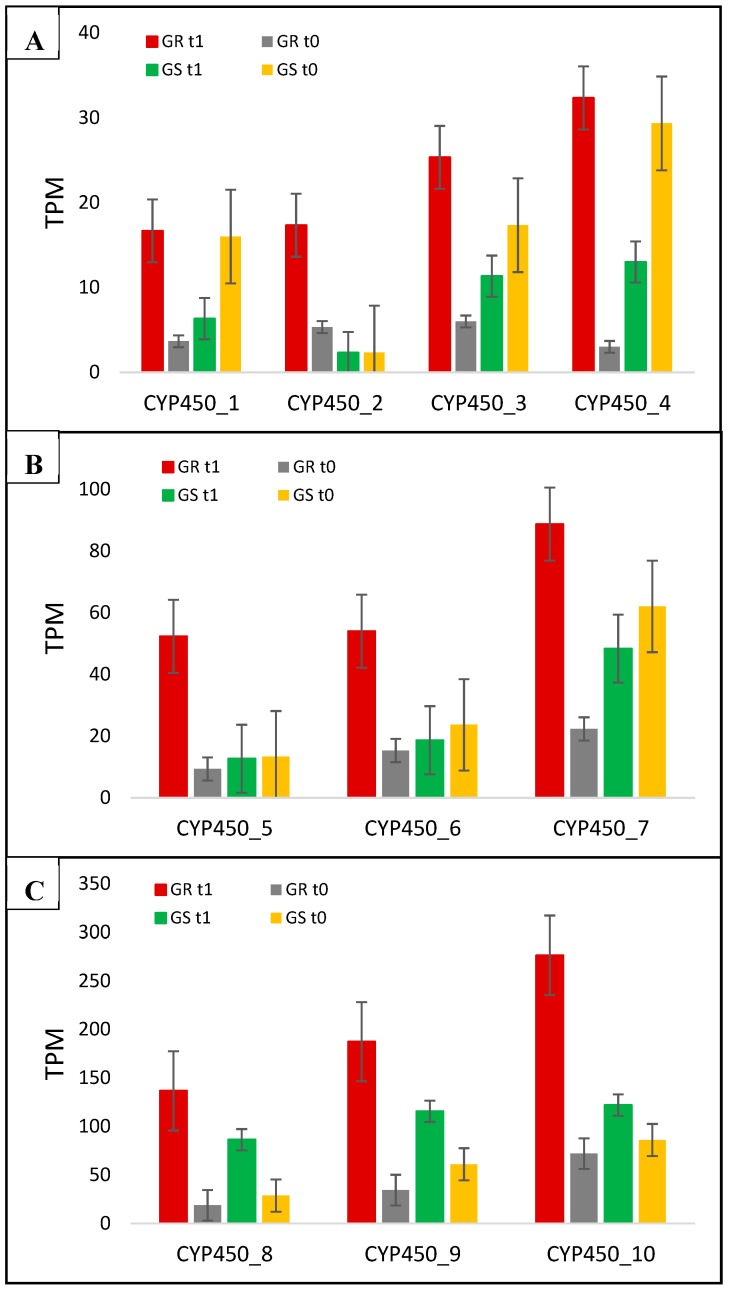
*C. bonariensis* transcriptome expression analysis (transcript reads per million mapped reads—TPM) of the 10 CYP450 genes (1 to 10). GR: glyphosate-resistant biotype; GS: glyphosate-sensitive biotype. t0: without glyphosate treatment; t1: with glyphosate treatment—RNA was obtained from plants collected at several timepoints up to 288 h after treatment and pooled. Intervals indicate the standard error. The phylogenetic tree of these genes is presented in Figure S10.

Among the 10 CYP450 genes we report in the present work in response to glyphosate treatment, four (CYP450_1, CYP450_3, CYP450_4, and CYP450_7) had expression suppressed in the GS biotype and increased in GR; three (CYP450_8-10) increased the transcription in both biotypes, although with higher levels in GR; and three (CYP450_2, CYP450_5, and CYP450_6) showed higher expression only in the resistant biotype with a fold change of 3.3, 5.9, and 4.5, respectively, in comparison between biotypes with treatment (Table 2; Figure 6). The CYP450_1 and CYP450_4 presented the highest differences in GR rather than GS in response to treatment, which was 11.5- and 27-fold, respectively (Table 2).

**Figure 7:**
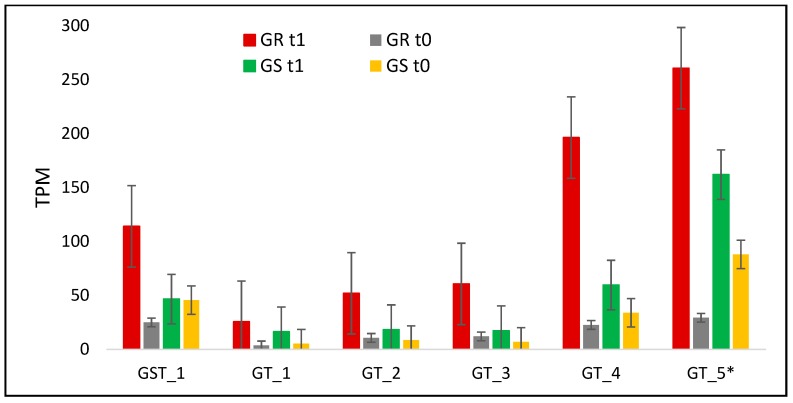
*C. bonariensis* transcriptome expression analysis (transcript reads per million mapped reads—TPM) of a glutathione (GST) and five glycosyltransferase (GT) genes. GR: glyphosate-resistant biotype; GS: glyphosate-sensitive biotype. t0: without glyphosate treatment; t1: with glyphosate treatment—RNA was obtained from plants collected at several timepoints up to 288 h after treatment and pooled; * indicates that was performed qRT-PCR analysis in that specific contig (See Figure 4). Intervals indicate the standard error.

The transcription level of glutathione-related gene increased only in the GR biotype in response to treatment with a ratio between biotypes of 4.6 (Table 3, Figure 7). The basal GST transcription level in the GR biotype was around 50% less than in GS (Figure 7). The expression levels for the five glycosyltransferase genes increased in both biotypes as a response to glyphosate treatment, although with a higher response in the GR biotype. In this way, the ratio between biotypes was almost doubled in GR rather than GS, except GT_4, which was of 4.8 (Figure 7, Table 3). GT_5 was evaluated in qRT-PCR in a time-course experiment and presented the highest response to treatment at 192 HAT in the GR biotype, while in GS, the expression levels had low variations (Figure 4).

**Figure 8:**
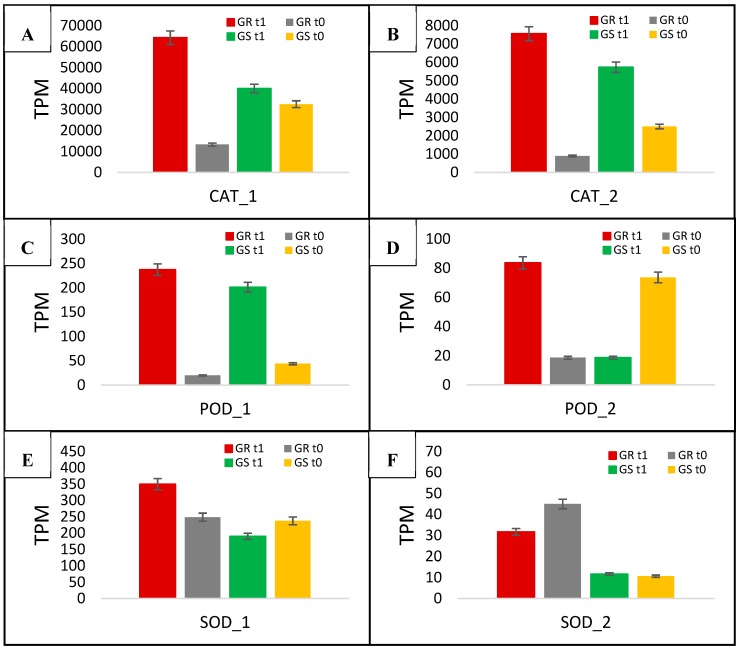
*C. bonariensis* transcriptome expression analysis (transcript reads per million mapped reads—TPM) of a two catalase (CAT—**A** and **B**), Peroxidase (POD—**C** and **D**), and superoxide dismutase (SOD—**E** and **F**) genes. GR: glyphosate-resistant biotype; GS: glyphosate-sensitive biotype. t0: without glyphosate treatment; t1: with glyphosate treatment—RNA was obtained from plants collected at several timepoints up to 288 h after treatment and pooled. Intervals indicate the standard error.

The two catalases had the highest expression after glyphosate treatment in both biotypes among all 41 candidate genes. The CAT_1 gene had >60,000 TPM, while CAT_2 was >7000. Glyphosate-treated GR plants had CAT_1, and CAT_2 increased transcription by 4.8- and 8.5-fold, whereas the GS biotype had increases of 1.2- and 2.3-fold, respectively (Figure 8A,B, Table 4). The comparison between treated biotypes indicates the higher expression levels of CAT_1 and CAT_2 in the resistant biotype at a ratio of 4- and 3.7-fold, respectively (Table 4). With regard to the two differentially expressed peroxidase genes, POD_1 presented a high response to glyphosate treatment in both biotypes, whereas POD_2 had increased expression in the GR biotype and was suppressed in GS plants (Figure 8C,D, Table 4). After glyphosate treatment, POD_1 increased the expression in GR biotype at 11.9-fold, while 4.6-fold in GS (ratio of 2.6). POD_2 expression increased 4.5-fold in GR and reduced at 0.2-fold in GS, presenting a ratio of 22.5 (Table 4). The gene expression related to SOD was higher in the GR biotype than in GS (Figure 8E,F, Table 4). SOD_2 presented high basal expression in the GR biotype, which reduced ~28% after glyphosate treatment; however, there was 2.7 times higher expression than in GS plants (Figure 8F).

In summary, the candidate gene results indicate that glyphosate resistance is a very complex process of plant defense against glyphosate action involving several molecular changes that allow GR plant survival. The new 41 candidate genes presented in the present study comprise a four-phase of a well-known process of herbicide resistance (oxidation; conjugation; transport; and degradation, detoxification, and protection). 

## 3. Discussion

### 3.1. The C. Bonariensis Transcriptome

The transcriptomic profiles performed up to 288 h after treatment (HAT) will assist in untangling the molecular processes underlying the glyphosate NTSR mechanisms in *C. bonariensis*. The current transcriptome assembly produced a large dataset, which likely includes the major candidate genes involved in glyphosate resistance in *Conyza*. The number of assembled contigs at the gene level in our study is similar in scope to those obtained in another *C. bonariensis* transcriptome study (~81,000) [16].

The gene ontology assignments of the differentially expressed genes (DEGs) showed interesting differences between biotypes. The GR biotype had a higher number of up-regulated genes related to transmembrane transport (biological process) and chloroplast (cellular component) than GS. On the other hand, the GS biotype presented a higher number of down-regulated genes related to these processes than GR (Figure 2; Figure 3). In our study, we focus on up-regulated transcripts in HR hairy fleabane, which indicate the putative importance of the transmembrane transport process mediated by ABC transporters in conferring glyphosate resistance. Upon application, glyphosate first impacts chloroplast biology [31]; therefore, the increase in transcription of genes related to chloroplast function in the GR biotype suggests the plant may be mounting a potential defense to protect plastids against glyphosate’s action. These results are in accordance with a study with proteomic analysis that identified chloroplast proteins differentially expressed as the primary sites of GR in *C. canadensis* [32]. Thus, the results of the present study suggest that transmembrane transport and chloroplast proteins might act in association to reduce damages caused by glyphosate action and allow the GR biotype to survive.

The substantially higher number of down-regulated genes related to translation (63 DEGs in GR vs. 16 in GS) and ribosome metabolism (52 DEGs in GR vs. 1 in GS) in GR suggest that in response to glyphosate action, the protein production was reduced in GR (Figure 3). Thus, one coping mechanism evolved in the GR biotype may be induced metabolic slowdown, which ameliorates the plant´ defense against glyphosate movement and damage. 

The EPSPS sequence analysis revealed no nucleotide substitutions at Thr 102 and Pro 106 codons and no increase in expression and number of copies in GR plants (Figure S7). Therefore, we conclude glyphosate resistance in the GR biotype is NTSR. Currently, few mutations that confer resistance to glyphosate have been observed in weeds [19]; ergo, glyphosate resistance across weedy plants is also NTSR. Few peptide changes in EPSPS are tolerated without loss of function because of most mutations are lethal [14,16,19]. Our results are in accordance with those related by Hereward et al. (2018) [16].

### 3.2. Candidate Genes Involved in Glyphosate Resistance in C. Bonariensis

A group of well-established NTSR gene families is the cytochrome (CYP450) monooxygenases, glutathione S-transferases (GSTs), and glycosyltransferases (GTs) [20,25,31,33]. These enzyme classes have versatile and multifunctional activities and might catalyze the oxidation and herbicide conjugation to variable substrates [20]. Further, a piece of emerging knowledge about the antioxidant system complementing the glyphosate resistance process has been described [34]. Metabolic-based herbicide resistance in general follows a four-phase process: I—oxidation; II—conjugation; III—transport; and IV—degradation, detoxification, and protection [14,20,30,35]. In the present work, we report transcriptional increases of the genes belonging to these four processes in a GR biotype. This increase in transcription of the enzyme-coding gene indicates a potential enhancement of activity, and subsequently glyphosate metabolization to allow GR plants to survive after herbicide exposure. To our knowledge, this is the first report that associated the CYP450, ABC transporters, GT, GST, and antioxidant enzyme differential gene expression to glyphosate resistance in *C. bonariensis*.

In phase I, CYP450 acts to oxidize the herbicide molecules and expose certain functional groups to phase II enzymes [20,25,36]. The P450s catalyze several reactions in plant metabolism, and their role in herbicide conversion is through hydroxylation or dealkylation [14,37,38]. P450s use electrons from NADPH (NADPH-P450 reductase) to insert an oxygen atom in the herbicide molecule, producing a more suitable product to be conjugated to glucose and transported to the vacuole [36]. The CYP450 enzyme has been reported to be involved in herbicide resistance to herbicides belonging to groups of acetanilide, aryloxyphenoxy-propanoate, imidazolinone, phenoxyalkanoic-acid, phenylurea, sulfonamide, sulfonylurea, bentazon, and clomazone herbicides [14,33,37,38]. However, to our knowledge, there is no report associating CYP450 to glyphosate resistance. In the present work, we report the up-regulation of 10 CYP450 genes in the GR biotype followed by glyphosate treatment showing the potential to be involved with GR in *C. bonariensis* (Figure 6, Table 2). Further, the annotated families of the 10 CYP450 contigs in the present work (Table 2) were not described by other authors conferring resistance to herbicide [33,36,37]. According to Powles and Yu (2010) [14], identifying the P450s conferring herbicide resistance in weeds is a vast research frontier, and this group of enzymes may indeed play significant roles in resistance owing to their ability to affect several herbicides modes of action.

In phase II, GSTs and GTs detoxify herbicides through direct conjugation or conjugate it to a wide range of substrates [20,35,39]. GSTs are found in the cytoplasm at high concentrations and work by catalyzing the glutathione conjugation to a several substrates, producing a polar product [20,39,40]. In this way, the up-regulation of GST and GT genes in the GR biotype followed by glyphosate treatment in the *C. bonariensis* might indicate the increase in activity of these enzymes to cope with glyphosate (Figure 7). GTs are a huge gene family in which proteins conjugate a sugar to a large variety of lipophilic molecules and herbicides [20,41]. GTs are in the cytoplasm and act by transferring sugar to lipophilic molecules enabling access to membrane transporters, including ABC transporters [41]. 

In phase III, ABC transporters may direct herbicides to be compartmentalized in the vacuoles or extracellular spaces after the action of CYP450, GSTs, and GTs and thus contribute to herbicide resistance process. ABC transporters are membrane-targeted proteins that require ATP for active transport of substrates across membranes [20,30]. Studies have been reported that ABC transporters are involved in glyphosate resistance in horseweed [23,24,42]. After analysis of the M10 and M11 ABC transporters genes previously reported in the literature as being involved in GR in *C. canadensis* [23], the results of the present study indicate that M10 and M11 genes do not appear to be differentially regulated in hairy fleabane and do not play a role in glyphosate resistance, which is consistent with other studies [16,21,32].

On the other hand, we present 19 new candidate target genes that might be involved in NTSR GR (Figure 5, Table 1). The relatively high number of ABC transporter genes with higher expression in the resistant biotype relative to the sensitive biotype indicate that this gene family may play an important role in the glyphosate-resistance process. Some of them were responsive to treatment in both biotypes, although with higher transcription in the GR biotype. The most interesting ABC genes are ABC_1, ABC_6, and ABC_11, which were only responsive to glyphosate treatment in the resistant biotype with a fold change ratio between biotypes of 3.1, 3.7, and 8, respectively (Figure 5, Table 1). Further, the time-course qRT-PCR results for two ABC transporter contigs indicates that the stimulus for an increase in expression levels might vary among members of this family of the gene at different times after herbicide exposure. For example, the ABC_12 had the highest expression at 24 HAT, while that for ABC_16 occurred at 192 HAT (Figure 4). These results clearly show that the gene transcription responses to glyphosate treatment do not occur simultaneously. A recent study involving ABC transporters genes in horseweed showed the importance of the timing of gene overexpression initiation for the resistance mechanism itself [42]. The same authors described that early overexpression of ABC transporters genes in a short time followed by glyphosate treatment could be more useful to sequester the herbicide to vacuoles or extracellular space. In this case, the delayed overexpression could not offer enough protection. Another transcriptional study with ABC transporter genes showed the influence of the environmental conditions such as glyphosate dosage and time after treatment, light, and temperature on gene induction [43]. Thus, several ABC transporters contigs seem to be gene stress-responsive because of the increase in the gene expression in both GR and GS biotypes. However, some of them presented higher regulation in the GR biotype (ABC_1, ABC_6, and ABC_11) and can be considered as related to the glyphosate-resistance mechanism in *C. bonariensis*.

If HR-associated ABC transporters sequester glyphosate into vacuoles or apoplasts [44], glyphosate would have mitigated early damage to sprayed tissues than in non-sequestered cells, and even more important, this would prevent effective transport to non-sprayed tissues. Indeed, this is likely the major basis of NTSR to glyphosate. In addition, if the transporters can also act in different secondary metabolites produced after glyphosate treatment, their early and late activity might help to ameliorate secondary stress. 

In phase IV, the oxidized herbicides by the CYP450 action and subsequently conjugated molecule are degraded in the vacuole or extracellular space, resulting in less phytotoxic molecules [20]. Further, in this stage, the antioxidant system works to scavenge reactive oxygen species (ROS) produced by the glyphosate action.

It is well known that glyphosate inhibits EPSPS to short-circuit the shikimic acid pathway, which results in decreased aromatic amino acid (phenylalanine, tyrosine, and tryptophan) biosynthesis. In so doing, shikimic acid accumulates, which ultimately leads to oxidative stress via ROS production [45]. ROS are highly reactive toxic molecules causing damage to cellular structures and cell death [46]. Further, the lack of tyrosine inhibits the synthesis of plastoquinone, which is an electron acceptor in the photosynthetic electron transport chain in photosystem two (PSII). The non-regeneration of plastoquinone interrupts the electron transport in PSII. Therefore, GS plants die from numerous factors beyond amino acid shortages [31]. 

Plants have an efficient enzymatic and non-enzymatic antioxidant system in response to ROS production [46,47]. The antioxidant enzyme-coding genes with differential expression in the present study were SOD, CAT, and POD (Figure 8). SOD removes the superoxide (O_2_^•−^) through catalyzing its dismutation and providing the first line of defense against ROS [46]. CAT can directly dismutase H_2_O_2_ into H_2_O and O_2_ and is indispensable for ROS detoxification during stressed conditions [47,48]. PODs play an essential role in scavenging ROS and protecting cells in higher plants through scavenging H_2_O_2_ in water–water and ascorbate-reduced glutathione cycles and utilize ascorbate as the electron donor [47]. The increase in the transcription of the antioxidant enzyme-coding genes SOD, CAT, and POD in resistant plants are in accordance with the previous study we reported for these enzymes’ activities; that study was performed using the same *C. bonariensis* as that used in this study [28]. In that study, we demonstrated the higher antioxidant enzyme activity in GR than in GS and less cellular damage after glyphosate treatment. Further, other studies of glyphosate-resistance mechanisms performed in *A. trifida* and *A. palmeri* concluded that antioxidant enzymes have the potential to play a role in the resistance processes [34,49]. Indeed, in the described antioxidant enzyme action, GSTs can also cope with ROS. GSTs are isozymes known to protect the cells against chemical-induced toxicity. These enzymes catalyze the conjugation of GSH to a variety of electrophilic and hydrophobic substrate [50]. Accordingly, the action of the cellular antioxidant machinery is essential to control excess ROS to protect plant cells from oxidative damage and to restore the redox homeostasis.

## 4. Materials and Methods 

### 4.1. Plant Accessions, Experimental Treatments, and RNA Isolation

Experiments were performed on GR and GS *C. bonariensis* biotypes from Pelotas, the Rio Grande do Sul State, Brazil, 32°04′05.91″ S, 52°52′59.14″ W. The resistance factor was twice determined to be 18.4 [28]. Seeds of each biotype were germinated in plastic trays containing sterilized soil and substrate (Mac plant—Mec Prec, Brazil) 3:1 and watered daily in a greenhouse at 30 °C (day) /20 °C (night) (±4 °C) with a 12-h photoperiod. Thirty days after emergence (30 DAE), seedlings of each biotype were transplanted to plastic pots of 3 L containing soil–substrate mix and kept in the same greenhouse and conditions. Thirty days after transplant (60 DAE; rosette stage—plants 6 to 8 cm in diameter), plants were treated with glyphosate (1480 g ae ha^−1^—Roundup Original DI 370 g ae L^−1^; Monsanto) with a CO_2_ sprayer and 150 L ha^−1^ of spray volume.

**Figure 9:**
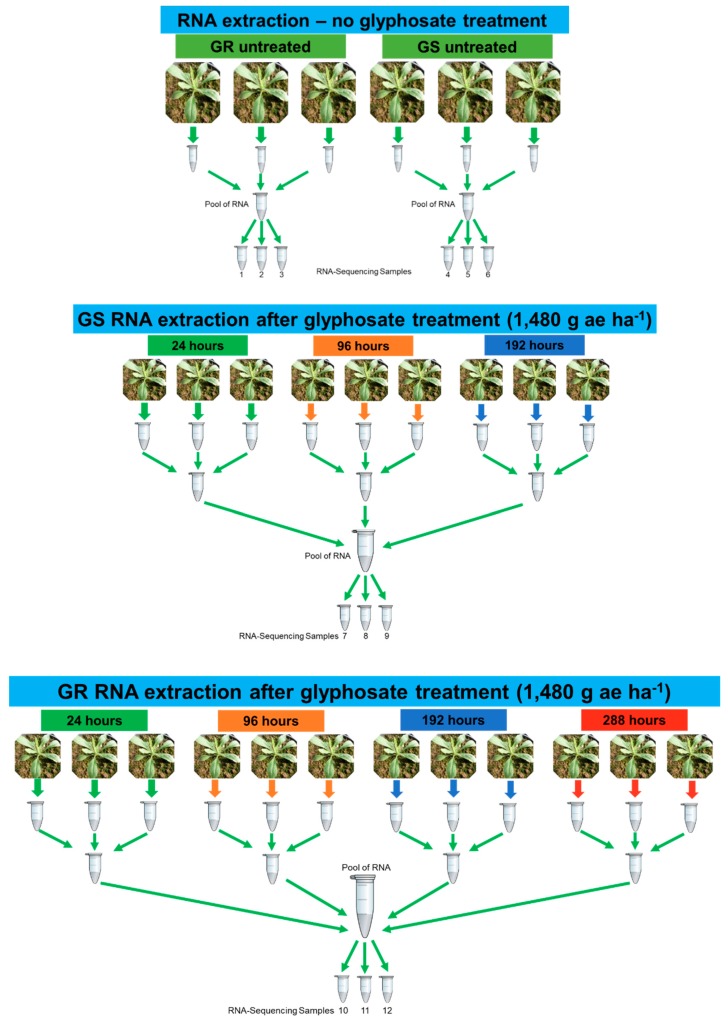
Experimental design for RNA extraction and RNA-Seq experiment in *Conyza bonariensis* glyphosate-resistant (GR) and -sensitive (GS) biotypes with (t1) and without (t0) glyphosate treatment. RNA samples 1–3: GR t0—untreated; RNA samples 4–6: GS t0—untreated; RNA samples 7–9: GS t1—RNA samples extracted at 24, 96, and 192 h after glyphosate treatment; RNA samples 10–12: GR t1—RNA extracted at 24, 96, 192, and 288 h after glyphosate treatment. RNA was extracted from a total of 27 plants and pooled according to each treatment. Three technical replicates were sequenced from each treatment (GR t0, GS t0, GS t1, and GR t1—a total of 12 libraries). Glyphosate rate of 1480 g ae ha^−1^.

The second and third leaves (from apex) from untreated and glyphosate-treated plants were collected in a range of timepoints: GR—untreated, 24, 96, 192, and 288 h after treatment; GS—untreated, 24, 96, and 192 h after treatment (Figure 9). Leaves from three biological replicates were collected at each time from a total of 27 plants (Figure 9). After collection, leaves were immediately frozen in liquid nitrogen and stored at −80 °C. The times to leaf collection and RNA extraction were determined based on a shikimic acid transient accumulation in the GR biotype, which increased substantially at 24 h after treatment, peaked at 96 h, and at 288 h presented a similar content to that of the untreated plants [28] (Figure S9). By contrast, the GS biotype had similar initial shikimic acid levels as GR plants at 24 h and 96 h, but it peaked at 192 h after treatment and died after that timepoint.

Total RNA was extracted from leaves from 27 plant samples using Trizol reagent (Invitrogen, Carlsbad, Calif, USA) in accordance with the manufacturer’s protocol (Figure 9). In the total RNA, an additional treatment with RNase-free DNase I (Invitrogen) was performed to remove residual genomic DNA. The RNA samples from each respective biotype and treatment (untreated and glyphosate treated) were pooled to form three technical replicates per treatment totaling 12 samples, i.e., GR untreated (GR t0), GS untreated (GS t0), GR treated (GR t1), and GS treated (GS t1) (Figure 9).

### 4.2. cDNA Library Construction and Illumina Sequencing

Before cDNA construction, the RNA integrity number (RIN) was measured in Bioanalyzer (Agilent Bioanalyzer 2100 system, Agilent Technologies, Santa Clara, Calif, USA). The library preparation of 12 cDNA samples and Illumina sequencing were performed at the Laboratory of Functional Genomics Applied to Agriculture and Agri-Energy, University of São Paulo (USP), São Paulo, SP, Brazil. The Illumina TruSeq Stranded mRNA LT Sample Prep Protocol was used to cDNA libraries construction. The 12 libraries were sequenced using the HiSeq Flow Cell v4, with the Illumina HiSeq 2500, producing 125 bp paired-end reads (2×).

### 4.3. De Novo Assembly and Functional Annotation

Raw data were filtered using FastQC (https://www.bioinformatics.babraham.ac.uk/) and trimmed adapter by Trimmomatic [51]. A single *de novo* assembly was generated using the reads from all treatments (12 libraries) with Trinity for further differential expression analysis [52]. For differential expression analysis, it is essential that all treatments be included in the transcriptome assembly [53,54]. Further, a single *de novo* assembly for each biotype (GR and GS) was generated for EPSPS sequence alignment. Trinity was executed using default settings in all assemblies and additional assembly analysis performed using the BioPython package [55]. The assembled transcripts generated by Trinity were aligned to the UniProt-trEMBL [56] database using Diamond [57], and only those with hits on plants (E-value threshold = 1 × 10^−10^) were selected for further analysis. 

The protein prediction was performed based on amino acid sequence using the Trinotate pipeline (https://trinotate.github.io/), which included the identification of protein families from the Pfam [57] and UniProt-SwissProt database, the identification of signal peptides and transmembrane proteins by SignalP and TMHMM, respectively [58], and rRNA prediction by RNAmmer [59]. The gene ontology functional categorization was performed by the BLASTx hits [60,61] with an E-value threshold of <10^−5^ from the non-redundant database according to molecular function, biological process, and cellular component ontologies.

### 4.4. Differential Expression Analysis

For differential expression analysis sections, we termed the assembled contig as “gene”. After data normalization, gene expression levels were calculated using transcript reads per million mapped reads (TPM) ≥1. For each treatment, gene-level expression was estimated using the Kallisto method [62] implemented in the Trinity accessory, which was used to generate an expression matrix. The matrix was processed in the edgeR mode [63] to perform the differential gene expression analysis and GO binning and enrichment was performed [63]. 

Differentially expressed genes (DEGs) were analyzed within each biotype with (t1) and without (t0) glyphosate treatment. DEGs were also filtered based on the false discovery rate (FDR) and *p-*value threshold at ≤0.001. After that, lists of all filtered DEGs were exported for each comparison and MA plots produced. In that case, DEGs with a log fold change ≥2 was considered up-regulated and ≤–2 as down-regulated. The up- and down-regulated genes of both biotypes for t1 and t0 treatments were categorized according to their GO functions using the same methods as those described above.

### 4.5. Quantitative Reverse Transcriptase Polymerase Chain Reaction (qRT-PCR) Analysis to Validate RNA-Seq Results

The same RNA samples from above were as converted into cDNA using RevertAid First Strand cDNA Synthesis (Thermo Fisher Scientific™), following the manufacturer’s protocol. The cDNA was pooled according to each biotype and time of collection after glyphosate treatment, resulting in 9 cDNA samples, and the qRT-PCR experiments performed using three technical replicates in a QuantStudio6^TM^ (Applied Biosystems^TM^). The reaction mixture of 15 µL contained 7.5 µL SYBR Green/ROX qPCR Master Mix (2×) (Thermo Fisher Scientific™), 1 µL of 1:3 diluted cDNA, 0.3 µL of each primer (forward and reverse) (10 µM), and the final volume was adjusted with appropriate amounts of RNAse free water. The QuantStudio6^TM^ thermal method was a multi-step of 10 min at 95 °C for pre-denaturation, 40 cycles of 15 s at 95 °C, and 1 min at 60 °C (2×). Data were collected during the extension step, and threshold-cycle (CT) values were calculated for each reaction using the Second Derivative Maximum method in the QuantStudio Real-Time PCR software (Applied Biosystems^TM^). 

For control reactions, no cDNA sample was added to the reaction mix of each studied primer. The sequence annotated as hexosyltransferase was used as an internal control (Contig: Trinity_DN_28781—F: CAATGGCCAGTCAAAACCAT; R: CCAGGCTCCATCCTATCGT A). The hexosyltransferase presented the best stability results among eleven candidate internal control genes tested, which included heat shock–protein and actin previously reported for *C. bonariensis* and *C. canadensis*, respectively [21,23].

Nineteen contigs with relative expression were selected from a transcriptome dataset based on the results of TPM of treated rather than untreated biotypes (GR—t1/t0; GS—t1/t0). In this case, in each respective replicate, TPM values >2 were considered as relative expression and ≤2 as absolute. Contigs with absolute expression were not considered for further analysis. Contigs with a low, medium, and high expression based on log fold change (logFC—t1/t0) results were selected from differential expression analysis to further analysis in qRT-PCR. The relative expression was calculated using the equation QR = 2^−(∆∆CT)^ [64]. The fold change results from transcriptome dataset were correlated to the time-course average of those from qRT-PCR using the Pearson model (r). The real-time results were subjected to ANOVA and averages compared by orthogonal contrasts at *p* ≤ 0.05 [64].

### 4.6. EPSPS Transcript Sequence Analysis

The sequences of the three EPSPS gene copies for *C. bonariensis* were obtained from GenBank (EPSPS1–EF200070; EPSPS2–EF200069; EPSPS3–EF200074) and mapped into the individual assembled transcriptomes of GR and GS using BLASTn command-line to identify their respective contigs. Trinity-assembled and GenBank sequences of EPSPS copies were translated to amino acids and aligned into BioEdit (http://www.mbio.ncsu.edu/BioEdit/) using the ClustalW multiple alignment functions with default settings. The alignment was performed to verify the occurrence of amino acid substitution at Threonine 102 and Proline 106, commonly altered reported regions to confer resistance to glyphosate [16,19]. Hence, the assembled EPSPS contigs were analyzed for transcription levels (TPM) to verify the overexpression occurrence in GR and GS in response to glyphosate treatment and copy number.

### 4.7. Selection of Similar Contig to Transport and Metabolic Glyphosate-Resistance Coding Gene

DEGs were selected by their UniProt/SwissProt assignment to gene families related to known roles in transportation and metabolic herbicide resistance [20,30]. We selected gene families of ABC transporters that were hypothesized to be involved in NTSR in *C. canadensis* [23], and cytochrome P450 (CYP450), glutathione (GST), glycosyltransferase (GT), related to be involved in herbicide metabolism [20,30,33]. Further, we selected DEGs annotated as antioxidant enzymes catalase (CAT), peroxidase (POD) and superoxide dismutase (SOD) reported with differential activities and to be involved in the reactive oxygen species (ROS) scavenging after glyphosate exposure in the same biotypes of *C. bonariensis* of the present study [28]. The antioxidant enzymes were also described as being involved in glyphosate resistance in *Amaranthus palmeri* and *Ambrosia trifida* [34,49]. 

The candidate genes were chosen according to the Venn diagram results from up-regulated genes in GR t0 (81 genes) and t1 (763 genes) and co-expressed in GR and GS plants (974 genes) in response to glyphosate treatment (Figure 1). The query DEGs were selected based on expression differences between GR (t1/t0) and GS (t1/t0) biotypes in response to glyphosate treatment (fold change ratio GR/GS). In this case, DEGs with a GR (t1/t0)/GS (t1/t0) ratio ≥2 were selected, except for the two SOD genes because they had a much higher basal expression in GR relative to GS plants. Finally, each gene sequence was searched against the UniProt/SwissProt database (www.uniprot.org/blast/) to assign a putative function. 

We obtained from Peng et al. (2010) [23] the sequences of ABC transporters M10 and M11 that were hypothesized to be implicated in NTSR to glyphosate in horseweed. These sequences were mapped in our whole *C. bonariensis* transcriptome using BLASTn, and after, their expression levels were evaluated using the TPM results for GR and GS plants in response to glyphosate treatment.

## 5. Conclusions

The present transcriptomic study revealed 41 new candidate NTSR genes that are annotated to be related to transport and metabolism in herbicide resistance. Among these candidates, there were 19 ABC transporters, 10 CYP450, one glutathione, and five glycosyltransferases. In addition, we also report the transcription results of two genes coding for antioxidant enzyme catalase, peroxidase, and superoxide dismutase. Thus, all target genes from different groups with a transcriptional increase in the glyphosate-resistant biotype might be acting in association to confer resistance to glyphosate in *C. bonariensis*. Further, these results indicate that gene expression in *C. bonariensis* varies among gene groups and within the same group, between biotypes, in response to glyphosate treatment and is dependent on the time after herbicide exposure. The present transcriptome study is the first report that associates various CYP450, ABC transporters, GT, GST, and antioxidant enzyme differential gene expression to glyphosate resistance in *C. bonariensis.* The results of the present work will serve as a data resource for further studies on the molecular mechanisms of glyphosate resistance in *C. bonariensis.* Further studies will involve functional genomic analysis using the protoplasts and gene editing approaches.

## Figures and Tables

**Table 1:** Differential expression of ABC transporter genes via RNA-Seq analysis of glyphosate-resistant (GR) and -sensitive (GS) *C. bonariensis* biotypes in response to glyphosate treatment up to 288 h after treatment. The transcriptional fold change data for the GR and GS biotypes are each expressed as the ratio of the ABC transporter gene expression from glyphosate treated to untreated individuals. Therefore, a value of 1 means the gene was neither induced nor repressed. The rightmost column represents the ratio of ratios; i.e., the magnitude of effect of being glyphosate-resistant.

N^o^	Contig ID	UniProt ID	Functional Category	Putative Localization	Transcription Fold Change		Ratio GR/GS
					GR	GS	
ABC_1	DN38339_c0_g1	Q9SKX0	ABC trans. C family 13	Integral component of membrane	3.7	1.2	3.1
ABC_2	DN41040_c1_g2	Q8LGU1	ABC trans. C family 8	Vacuolar membrane	17	2.4	7.1
ABC_3	DN27993_c1_g1	Q7DM58	ABC trans. C family 4	Plasma membrane/Vacuole	9.5	3.6	2.6
ABC_4	DN37018_c0_g1	Q9C8H0	ABC trans. C family 12	Integral component of membrane	15.6	3.5	4.5
ABC_5	DN25809_c0_g1	Q8LGU1	ABC trans. C family 8	Vacuole	3.5	0.5	7
ABC_6	DN38603_c0_g1	Q9ZR72	ABC trans. B family 1	Plasma membrane	3.3	0.9	3.7
ABC_7	DN41040_c1_g1	Q8LGU1	ABC trans. C family 8	Vacuole	18	3	6
ABC_8	DN42180_c1_g3	Q7DM58	ABC trans. C family 4	Golgi apparatus/Plasma and vacuole membrane	3.9	1.9	2
ABC_9	DN41654_c6_g1	Q9ZU35	ABC trans. G family 7	Chloroplast	4	1.3	3.1
ABC_10	DN23566_c0_g1	Q8LGU1	ABC trans. C family 8	Vacuole	4.2	0.6	7
ABC_11	DN42180_c1_g1	Q7DM58	ABC trans. C family 4	Golgi apparatus/Plasma and vacuole membrane	8	1	8
ABC_12	DN32993_c0_g1	Q7DM58	ABC trans. C family 14**	Golgi apparatus/Plasma and vacuole membrane	19	2.8	6.8
ABC_13	DN41851_c0_g1	Q9ZR72	ABC trans. B family 1	Plasma membrane	3.5	1.4	2.5
ABC_14	DN37243_c0_g1	Q9C9W0	ABC trans. I family 17	Plasma membrane and Vacuole	4.1	1.6	2.6
ABC_15	DN37746_c1_g1	Q9FNU2	ABC trans. B family 27	Vacuole	14.6	5.9	2.5
ABC_16	DN41980_c0_g1	Q9M1C7	ABC trans. C family 9**	Vacuole	23.3	7.6	3.1
ABC_17	DN29514_c0_g1	Q9FNU2	ABC trans. B family 25	Vacuole	6.9	1.5	4.6
ABC_18	DN41405_c0_g1	Q9LYS2	ABC trans. C family 10	Vacuole	17.9	7.8	2.3
ABC_19	DN42119_c0_g1	Q42093	ABC trans. C family 2	Vacuole	3.8	1	3.8

Differentially expressed genes were selected using a *p*-value and FDR threshold set at ≤0.001; t0 = without glyphosate treatment; t1 = with glyphosate treatment—RNA was obtained from plants collected at several timepoints up to 288 h after treatment and pooled; GR: glyphosate-resistant biotype; GS: glyphosate-sensitive biotype. ** indicates that the gene was subjected to qRT-PCR analysis (See Figure 4). The phylogenetic tree of these genes is presented in Figure S10.

**Table 2:** Differential expression of cytochrome P450 (CYP450) genes via RNA-Seq analysis of glyphosate-resistant (GR) and -sensitive (GS) *C. bonariensis* biotypes in response to glyphosate treatment up to 288 h after treatment. The transcriptional fold change data for the GR and GS biotypes are each expressed as the ratio of the ABC transporter gene expression from glyphosate treated to untreated individuals. Therefore, a value of 1 means the gene was neither induced nor repressed. The rightmost column represents the ratio of ratios; i.e., the magnitude of effect of being glyphosate-resistant.

N^o^	Contig ID	UniProt ID	Functional Annotation	Putative Localization	Transcription Fold Change		Ratio GR/GS
					GR	GS	
CYP450_1	DN41388_c1_g1	O49858	CYP450 82A3	Membrane	4.5	0.4	11.5
CYP450_2	DN27559_c0_g1	Q9STK7	CYP450 71A26	Integral component of membrane	3.3	1.0	3.3
CYP450_3	DN22805_c0_g1	A0A103YCT3	CYP450	Uncharacterized	4.2	0.7	6.0
CYP450_4	DN35527_c1_g1	A0A103XYM0	CYP450 78A5	Integral component of membrane	10.8	0.4	27
CYP450_5	DN15853_c0_g1	A0A251TZE7	CYP450 71B7	Endoplasmic reticulum	5.6	1.0	5.6
CYP450_6	DN34846_c0_g1	Q9LMX7	CYP450 78A5	Endoplasmic reticulum	3.5	0.8	4.5
CYP450_7	DN35866_c0_g1	A0A251SVC5	CYP450 76C2	Uncharacterized	4.0	0.8	5.0
CYP450_8	DN25141_c0_g1	O81973	CYP450 93A3	Integral component of membrane	7.3	3.0	2.4
CYP450_9	DN31873_c0_g1	A0A103YMI2	CYP450 94A1	Integral component of membrane	5.5	1.9	2.9
CYP450_10	DN73328_c0_g1	A0A251RP29	CYP450 82A4	Integral component of membrane	3.8	1.4	2.7

Differentially expressed genes were selected using a *p-*value and FDR threshold set at ≤0.001; t0 = without glyphosate treatment; t1 = with glyphosate treatment—RNA was obtained from plants collected at several timepoints up to 288 h after treatment and pooled; GR: glyphosate-resistant biotype; GS: glyphosate-sensitive biotype. The phylogenetic tree of these genes is presented in Figure S10.

**Table 3:** Differential expression of glutathione (GST) and glycosyltransferase (GT) genes via RNA-Seq analysis of glyphosate-resistant (GR) and -sensitive (GS) *C. bonariensis* biotypes in response to glyphosate treatment up to 288 h after treatment. The transcriptional fold change data for the GR and GS biotypes are each expressed as the ratio of the ABC transporter gene expression from glyphosate treated to untreated individuals. Therefore, a value of 1 means the gene was neither induced nor repressed. The rightmost column represents the ratio of ratios; i.e., the magnitude of effect of being glyphosate-resistant.

N^o^	Contig ID	UniProt ID	Functional Annotation	Putative Localization	Transcription Fold Change		Ratio GR/GS
					GR	GS	
GST_1	DN34237_c0_g1	Q9M0G0	Glutathione hydrolase 3	Vacuole	4.6	1	4.6
GT_1	DN32482_c0_g1	Q6VAA9	Glycosyltransferase 73E1 UDP	Uncharacterized	7	3.1	2.2
GT_2	DN30716_c0_g1	Q9C768	Glycosyltransferase 76B1 UDP	Intracellular membrane-bounded organelle	4.9	2.1	2.3
GT_3	DN44583_c0_g1	Q6VAB0	Glycosyltransferase 85C2 UDP	Uncharacterized	5.1	2.5	2
GT_4	DN30626_c0_g1	Q9LZD8	Glycosyltransferase 89A UDP	Intracellular membrane-bounded organelle	8.7	1.8	4.8
GT_5	DN23238_c0_g1	Q6VAA4	Glycosyltransferase 85C1 UDP**	Uncharacterized	13.4	6.8	2

Differentially expressed genes were selected using a *p*-value and FDR threshold set at ≤0.001; t0 = without glyphosate treatment; t1 = with glyphosate treatment—RNA was obtained from plants collected at several timepoints up to 288 h after treatment and pooled; GR: glyphosate-resistant biotype; GS: glyphosate-sensitive biotype. ** indicates that the gene was subjected to qRT-PCR analysis (See Figure 4).

**Table 4:** Differential expression of catalase (CAT), peroxidase (POD), and superoxide dismutase (SOD) genes via RNA-Seq analysis of glyphosate-resistant (GR) and -sensitive (GS) *C. bonariensis* biotypes in response to glyphosate treatment up to 288 h after treatment. The transcriptional fold change data for the GR and GS biotypes are each expressed as the ratio of the ABC transporter gene expression from glyphosate treated to untreated individuals. Therefore, a value of 1 means the gene was neither induced nor repressed. The rightmost column represents the ratio of ratios; i.e., the magnitude of effect of being glyphosate-resistant.

N^o^	Contig ID	UniProt ID	Functional Annotation	Putative Localization	Transcription Fold Change		Ratio GR/GS
					GR	GS	
CAT_1	DN41650_c0_g1	P45739	Catalase	Peroxisome	4.8	1.2	4
CAT_2	DN32938_c3_g1	P29756	Catalase-1/2	Peroxisome	8.5	2.3	3.7
POD_1	DN35984_c0_g1	Q9SZB9	Peroxidase 47	Extracellular region or secreted	11.9	4.6	2.6
POD_2	DN34083_c0_g1	Q9SJZ2	Peroxidase 17	Extracellular region or secreted	4.5	0.2	22.5
SOD_1	DN36049_c1_g1	Q9FMX0	Superoxide dismutase 3	Chloroplast	1.4	0.8	1.7
SOD_2	DN32389_c0_g1	O04996	Superoxide dismutase	Cytoplasm	0.7	1.1	0.6

Differentially expressed genes were selected using a *p*-value and FDR threshold set at ≤0.001; t0 = without glyphosate treatment; t1 = with glyphosate treatment—RNA was obtained from plants collected at several timepoints up to 288 h after treatment and pooled; GR: glyphosate-resistant biotype; GS: glyphosate-sensitive biotype.

## Data Availability

The RNA-Seq data generated in this study have been uploaded into the NCBI-SRA database under bio project PRJNA436902 and the accession numbers SRA: SRS3018834-SRS3018845. Available at: https://www.ncbi.nlm.nih.gov/bioproject/PRJNA436902/.

## Supplementary Materials

The following are available online at https://www.mdpi.com/2223-7747/8/6/157/s1, Figure S1. Length distribution of transcripts assembled from transcriptome libraries of *C. bonariensis*. Figure S2. Sequence comparison to other organisms from the distribution of BLASTx hits (e-value < 1 × 10^−10^) against the non-redundant protein database of the National Center for Biotechnology Information. Figure S3. Sequence comparison to other plants (hit ≥1%) from the distribution of BLASTx hits (e-value < 1 × 10^−10^) against the non-redundant protein database of the National Center for Biotechnology Information (NCBI). Figure S4. Top ten Gene Ontology (GO) terms identified in the *C. bonariensis* transcriptome assembly summarized in three main categories: (A) biological process, (B) molecular function, and (C) cellular component. Figure S5. MA plot of differential expression analysis generated by EdgeR from transcriptome study performed in *C. bonariensis* glyphosate-resistant (GR) and -sensitive (GS) biotypes in response to glyphosate treatment. Dots above zero are up-regulated, and dots below are down-regulated. Red dots indicate significant expression at an adjusted *p-*value and false discovery rate (FDR) threshold set at ≤0.001, and log2FC ≥ 2 (up-regulated) or ≤log2FC (down-regulated). Plots for each contig its log2FC (fold change) (A, Y-axis) vs. its counts (mean of normalized counts) (M, X-axis). t0 = without glyphosate treatment; t1 = with glyphosate treatment. GR t1: RNA sampled at 24, 96, 192, and 288 h after treatment and pooled; GS t1: RNA sampled at 24, 96, and 192 h after treatment and pooled. Figure S6. Correlation of transcriptomic and qRT-PCR (average of time-course) expression levels results of 19 genes of glyphosate-resistant (GR) and -sensitive (GS) *C. bonariensis* biotypes. Figure S7. Partial sequence alignment of the EPSPS transcripts and amino acid sequence assembled of glyphosate-resistant (GR) and -sensitive (GS), and a sensitive *C. bonariensis* sequence from GenBank. (A) EPSPS1 (GenBank—accession number EF200070); (B) EPSPS2 (GenBank—accession number EF200069); (C) EPSPS3 (GenBank—accession number EF200074). The red boxed amino acids show no substitution at positions Threonine 102 and Proline 106. (D) Transcriptome expression levels (transcript reads per million mapped reads—TPM) of the three EPSPS copies in GR and GS in response to glyphosate treatment and expression difference (Fold change). GR: glyphosate-resistant biotype; GS: glyphosate-sensitive biotype. t0: without glyphosate treatment; t1: with glyphosate treatment. Figure S8. *C. bonariensis* transcriptome expression analysis (transcript reads per million mapped reads—TPM) of the M10 and M11 ABC transporters reported to be involved in glyphosate resistance in *C. canadensis* by Peng et al. (2010). M10_c1: Score (Bits) 2682, *E-*value zero; M10_c2: Score 608, *E-*value 1 × 10^−72^; M11_c1: Score 2669, *E-*value zero; M11_c2:1232, *E-*value zero. GR: glyphosate-resistant biotype; GS: glyphosate-sensitive biotype. t0: without glyphosate treatment; t1: with glyphosate treatment. Contigs were filtered according to the *p-*value and false discovery rate (FDR) threshold set at ≤0.001. Intervals indicate the standard error. Figure S9. Responses of *C. bonariensis* glyphosate-resistant (GR) and -sensitive (GS) biotypes to glyphosate treatment (1480 g ae ha^−1^). (A) The shikimic acid content in a time-course experiment; and (B) plants status at 288 h after glyphosate treatment (12 days). GS plants died 192 h after treatment, whereas GR plants survived. Leaf samples were collected for RNA extraction based on shikimic acid content curve in GR biotype. Then RNA was extracted from untreated plants in GR and GS biotypes, and treated GR plants at 24, 96, 192, and 288 h after treatment, and at 24, 96, and 192 h in the GS plants, which died 192 h after treatment. Figure S10. Evolutionary analysis of the candidate contigs sequences similarity to be related to glyphosate-resistance in *C. bonariensis* obtained from a transcriptome. (A) ABC transporters; (B) cytochrome P450 (CYP450). The number in front of the contig description indicate the order presented in each respective table 1 and 2. ABCC5A *Arabidopsis*: ABC Transporter full sequence obtained from GenBank *Arabidopsis thaliana*; M10 and M11 ABC: sequences of ABC transporters obtained from Peng et al. (2010). The evolutionary history was inferred by using the Maximum Likelihood method and the Tamura-Nei model. The bootstrap consensus tree inferred from 500 replicates is taken to represent the evolutionary history of the taxa analyzed. Branches corresponding to partitions reproduced in less than 50% bootstrap replicates are collapsed. The percentage of replicate trees in which the associated taxa clustered together in the bootstrap test (500 replicates) are shown next to the branches. Supplementary Sequences: .docx file.
