# Supplementary material for: Transcriptomic Analysis Identifies New Non-Target Site Glyphosate-Resistance Genes in Conyza bonariensis
Source: Plants (Basel). 2019 Jun 7;8(6):157. doi: 10.3390/plants8060157 (PMC6630842; doi:10.3390/plants8060157)
Supplement: Supplementary file 1 [file plants-08-00157-s001.pdf]

Supplementary Materials

Transcriptomic Analysis Identifies New Non-Target Site Glyphosate-Resistance Genes in *Conyza bonariensis*

Cristiano Piasecki [1,2,*], Yongil Yang [2], Daiane P. Benemann [1], Frederico S. Kremer [3], Vanessa Galli [3], Reginald J. Millwood [2], Joanei Cechin [1], Dirceu Agostinetto [1], Luciano C. Maia [4], Leandro Vargas [5] and C. Neal Stewart Jr. [2,*]

Table 1. Summary of the Illumina sequencing and *de novo* assembly statistics of *Conyza bonariensis* transcriptome.

Description	Assembly Stats*
Total assembled bases	157,784,873
Total of paired-end reads	23,383,488
Total of transcripts	203,054
Total of contigs "gene" level	90,124
Contig N50	1,118
Average contig length (bp)	777.06
GC (%)	40.23

* Assembled from all biotypes and treatments, a total of 12 libraries.

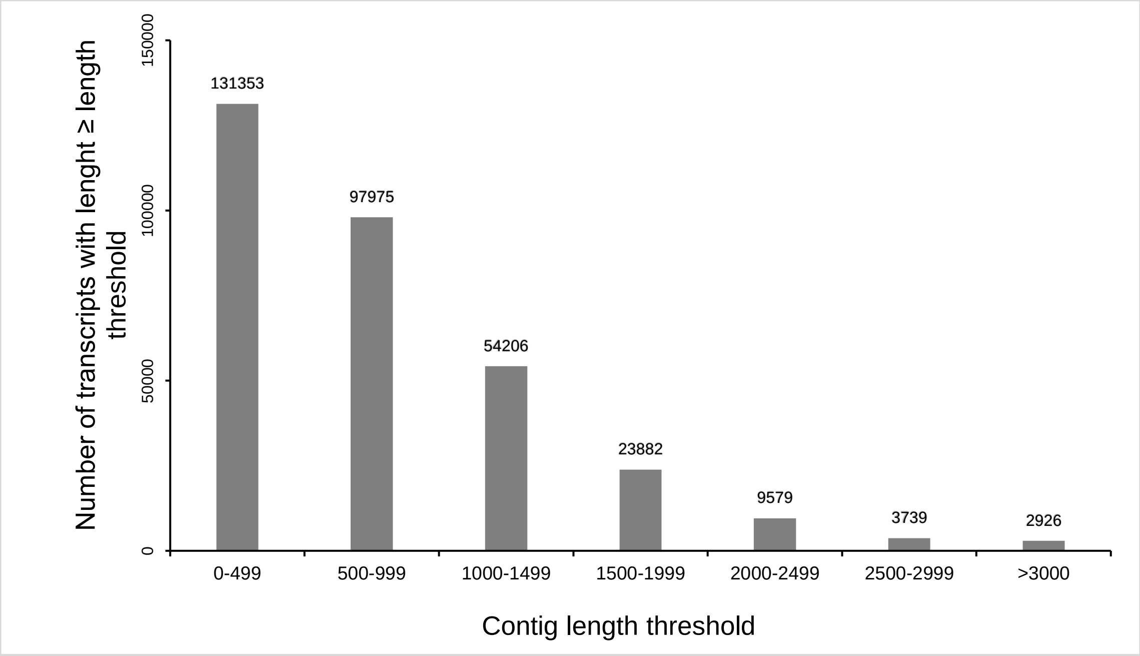

Figure 1. Length distribution of transcripts assembled from transcriptome libraries of hairy fleabane.

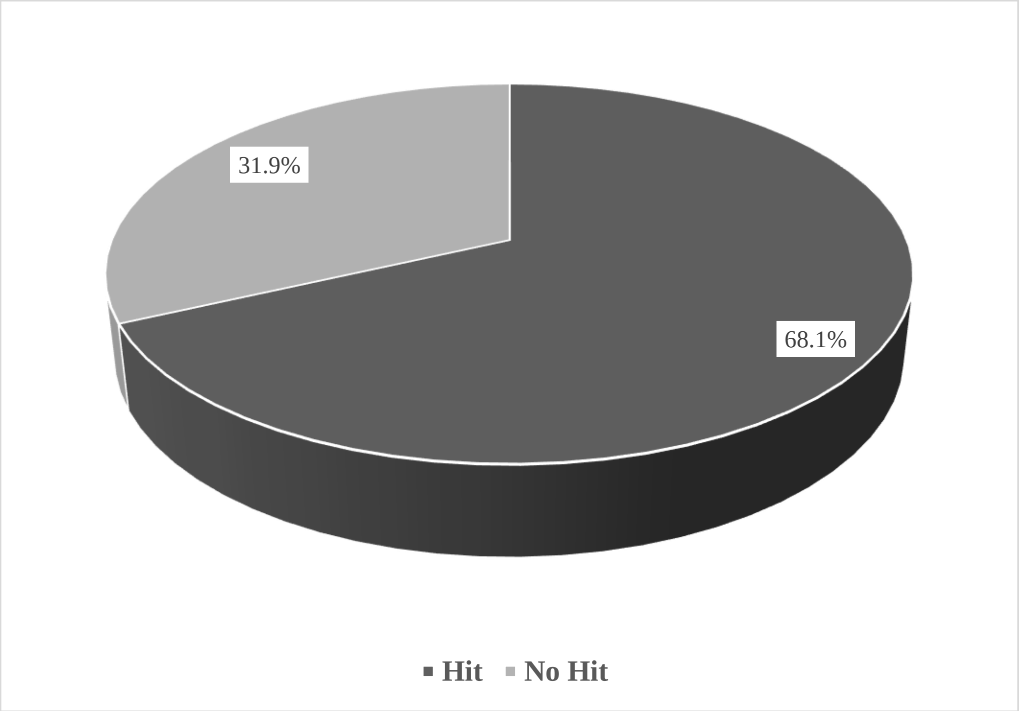

Figure 2. Sequence comparison to other organisms from the distribution of BLASTx hits (e-value < 1e−10) against the non-redundant protein database of the National Center for Biotechnology Information.

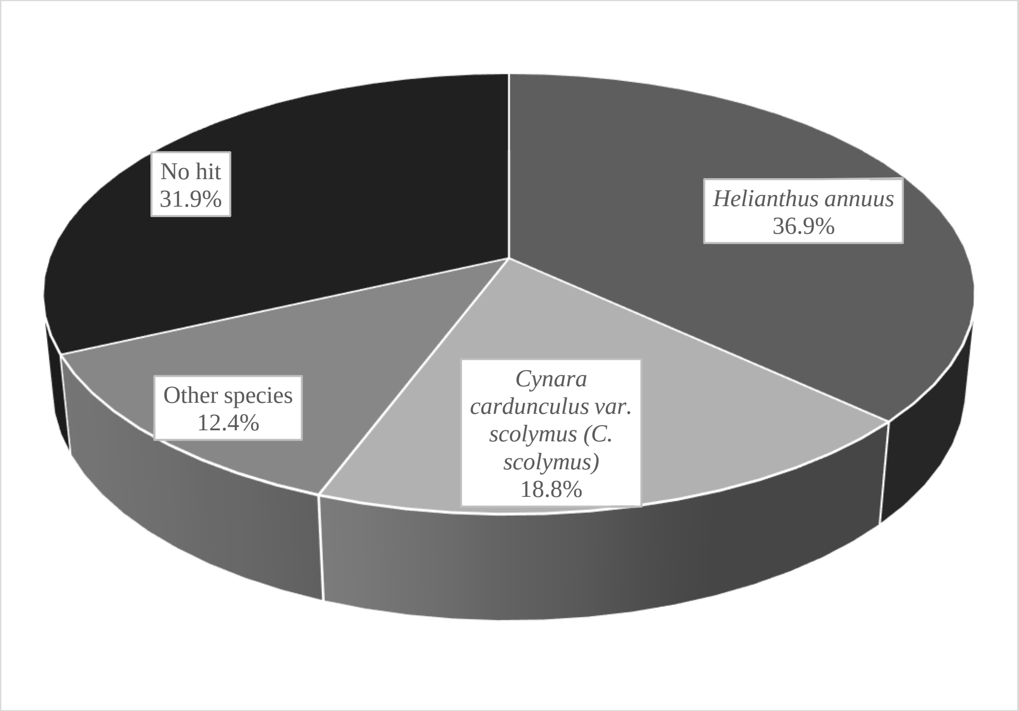

Figure 3. Sequence comparison to other plants (hit ≥ 1%) from the distribution of BLASTx hits (e-value < 1e−10) against the non-redundant protein database of the National Center for Biotechnology Information (NCBI).

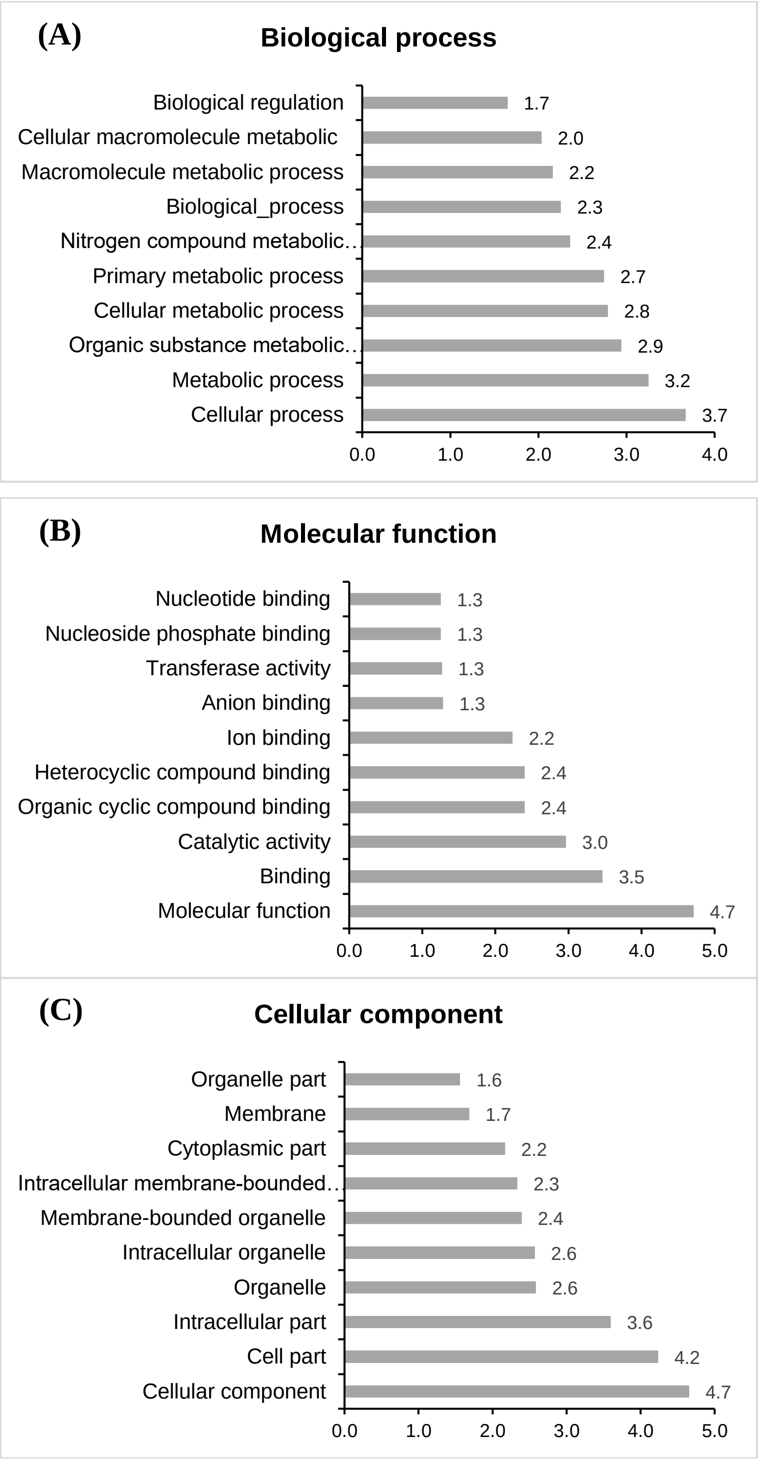

Figure 4. Top ten Gene Ontology (GO) terms identified in the hairy fleabane transcriptome assembly summarized in three main categories: (A) biological process, (B) molecular function, and (C) cellular component.

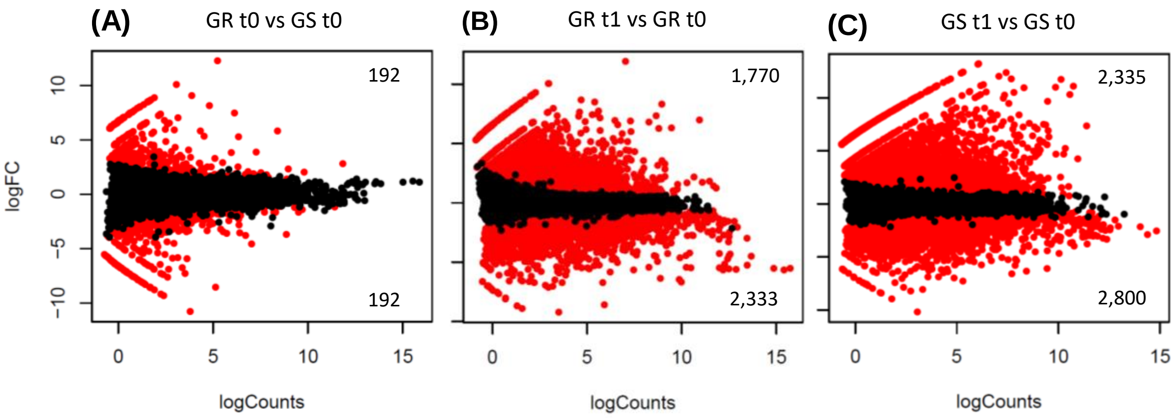

Figure 5. MA plot of differential expression analysis generated by EdgeR from transcriptome study performed in *Conyza bonariensis* glyphosate-resistant (GR) and -sensitive (GS) biotypes in response to glyphosate treatment. Dots above zero are up-regulated, and dots below are down-regulated. Red dots indicate significant expression at an adjusted *p-value* and false discovery rate (FDR) threshold set at ≤ 0.001, and log2FC ≥ 2 (up-regulated) or ≤ log2FC (down-regulated). Plots for each contig its log2FC (fold change) (A, Y-axis) vs. its counts (mean of normalized counts) (M, X-axis). t0 = without glyphosate treatment; t1 = with glyphosate treatment – RNA was obtained from plants collected at several timepoints up to 288 h after treatment and pooled. GR t1: RNA sampled at 24, 96, 192, and 288 hours after treatment and pooled; GS t1: RNA sampled at 24, 96, and 192 hours after treatment and pooled.

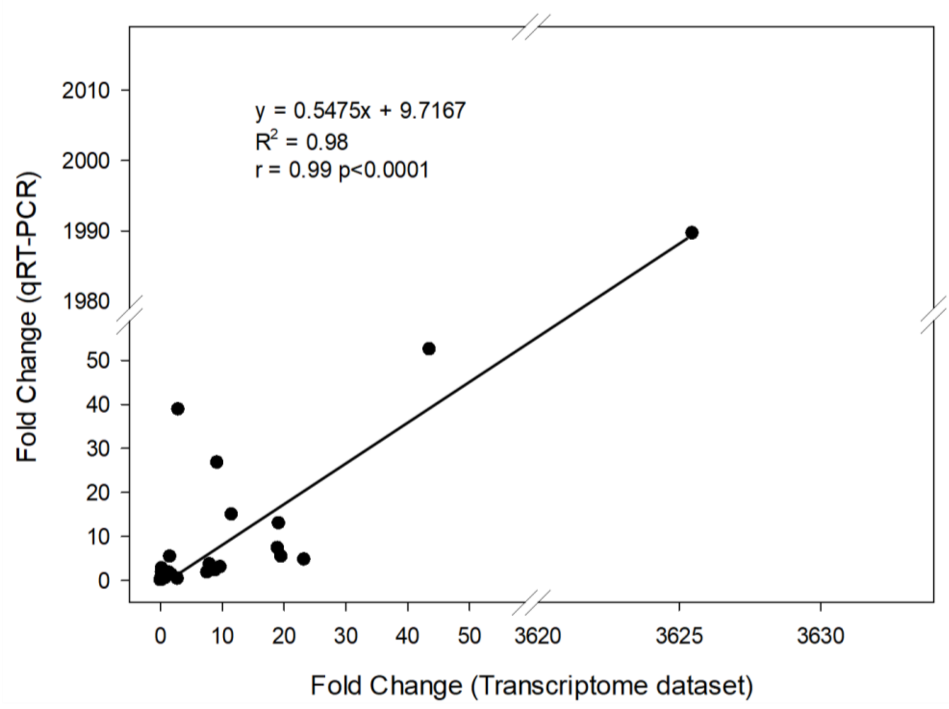

Figure 6. Correlation of transcriptomic and qRT-PCR (average of time-course) expression levels results of 19 genes of glyphosate-resistant (GR) and -sensitive (GS) *Conyza bonariensis* biotypes.

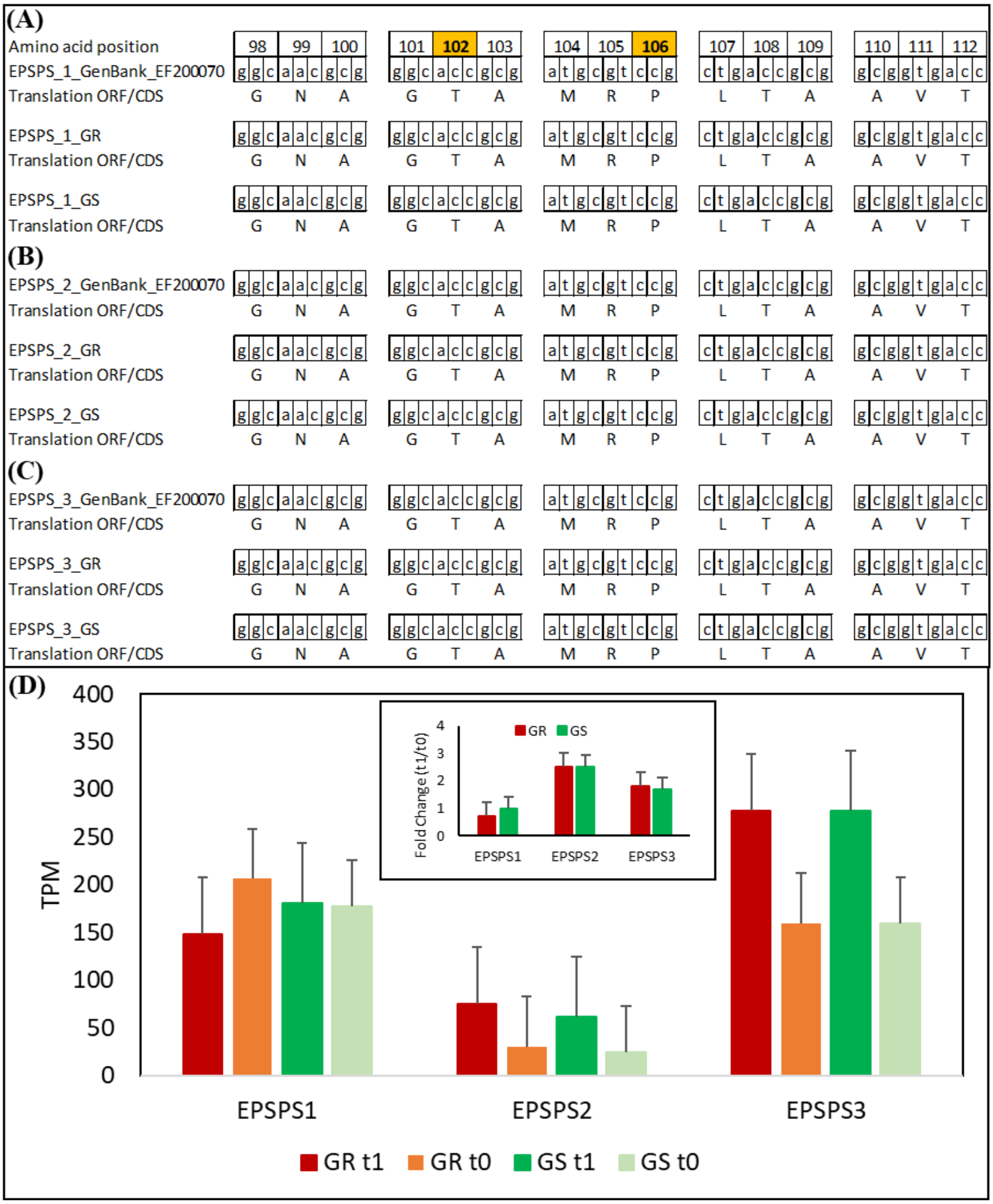

Figure 7. Partial sequence alignment of the EPSPS transcripts and amino acid sequence assembled of glyphosate-resistant (GR) and -sensitive (GS), and a sensitive *C. bonariensis* sequence from GenBank. (A) EPSPS1 (GenBank - accession number EF200070); (B) EPSPS2 (GenBank accession number EF200069); (C) EPSPS3 (GenBank accession number EF200074). The red boxed amino acids show no substitution at positions Threonine 102 and Proline 106. (D) Transcriptome expression levels (transcript reads per million mapped reads - TPM) of the three EPSPS copies in GR and GS in response to glyphosate treatment and expression difference (Fold change). GR: glyphosate-resistant biotype; GS: glyphosate-sensitive biotype. t0: without glyphosate treatment; t1: with glyphosate treatment – RNA was obtained from plants collected at several timepoints up to 288 h after treatment and pooled.

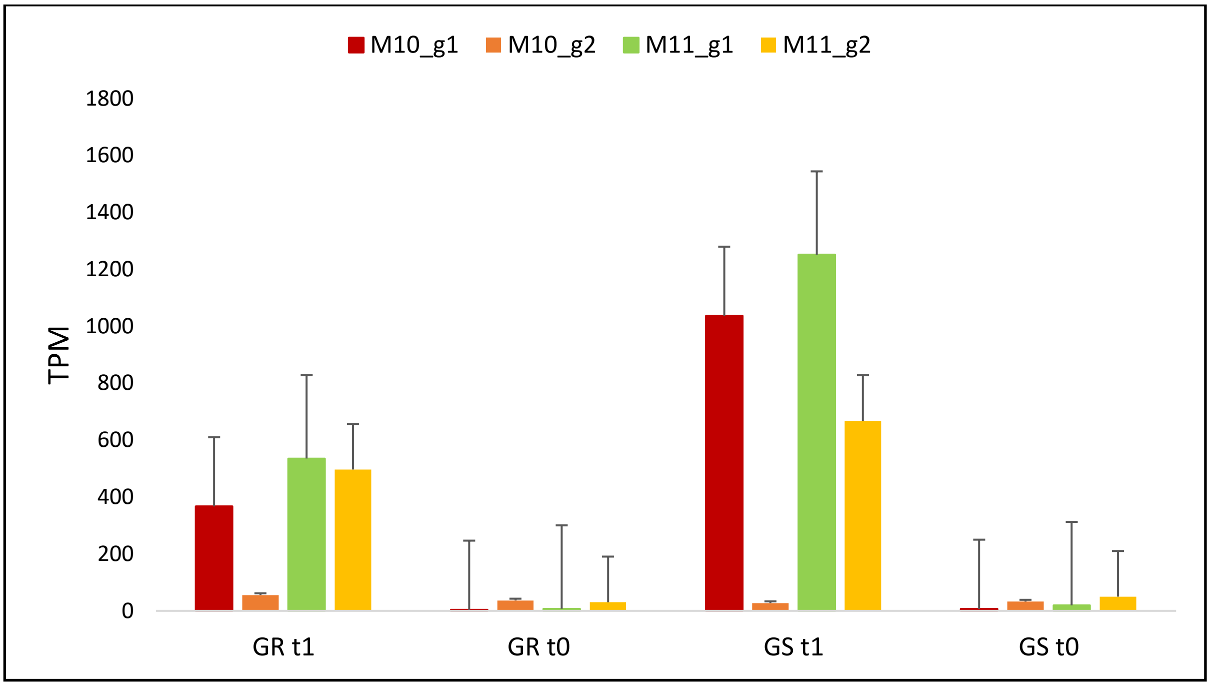

Figure 8. *Conyza bonariensis* transcriptome expression analysis (transcript reads per million mapped reads - TPM) of the M10 and M11 ABC Transporters reported being involved in glyphosate resistance in *C. canadensis* by Peng et al. (2010). M10_c1: Score (Bits) 2,682, *E-value* zero; M10_c2: Score 608, *E-value* 1e-72; M11_c1: Score 2,669, *E-value* zero; M11_c2:1,232, *E-value* zero. GR: glyphosate-resistant biotype; GS: glyphosate-sensitive biotype. t0: without glyphosate treatment; t1: with glyphosate treatment – RNA was obtained from plants collected at several timepoints up to 288 h after treatment and pooled. Contigs were filtered according to the *p-value* and false discovery rate (FDR) threshold set at ≤ 0.001. Intervals indicate the standard error. Adapted from Piasecki et al. (2019).[27]

a)

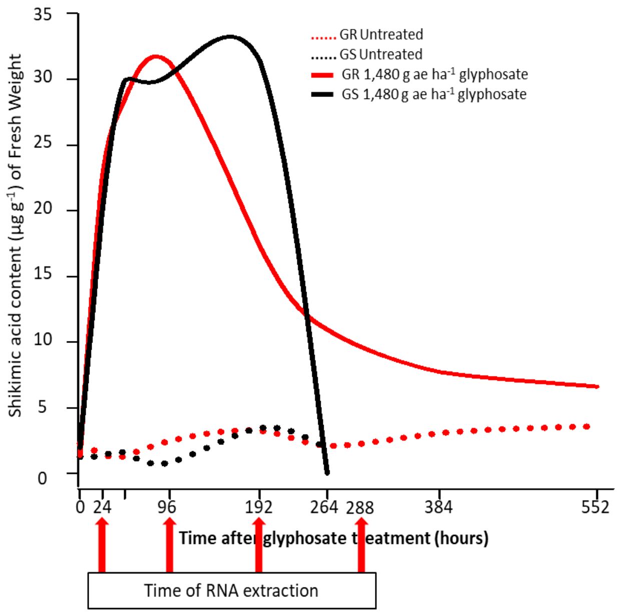

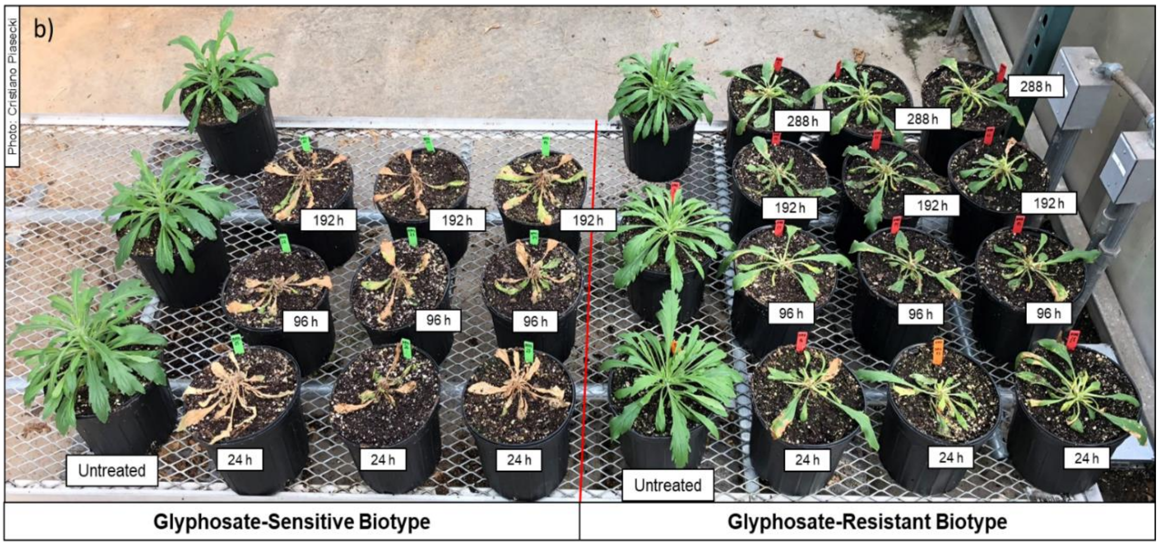

Figure 9. Responses of *Conyza bonariensis* glyphosate-resistant (GR) and -sensitive (GS) biotypes to glyphosate treatment (1,480 g ae ha^{-1}). a) The shikimic acid content in a time-course experiment - Adapted from Piasecki et al. (2019) [28]; and b) Plants status at 288 hours after glyphosate treatment (12 days). 24 h, 96 h, 192 h, and 288 h illustrated the time after glyphosate treatment that leaves were collected for RNA extraction. GS plants died after 192 h after treatment whereas GR plants survived. Leaf samples were collected for RNA extraction based on shikimic acid content curve in GR biotype. Then RNA was extracted from untreated plants in GR and GS biotypes, and treated GR plants at 24, 96, 192, and 288 h after treatment, and at 24, 96, and 192 h in the GS plants which died after 192 h after treatment.

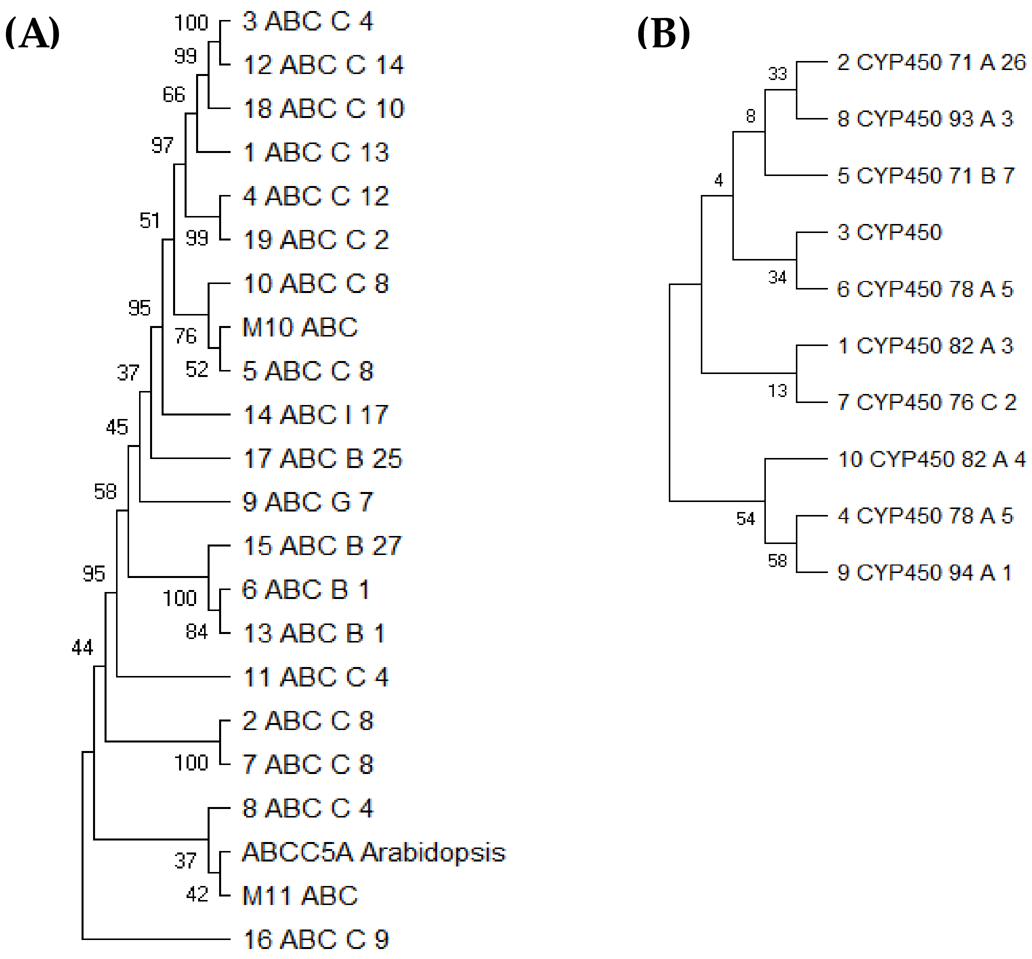

Figure 10. Evolutionary analysis of the candidate contigs sequences similarity to be related to glyphosate-resistance in *C. bonariensis* obtained from a transcriptome. A) ABC transporters; B) cytochrome P450 (CYP450). The number in front of the contig description indicate the order presented in each respective table 1 and 2. ABCC5A *Arabidopsis*: ABC Transporter full sequence obtained from GenBank *Arabidopsis thaliana;* M10 and M11 ABC: sequences of ABC transporters obtained from Peng et al. (2010). The evolutionary history was inferred by using the Maximum Likelihood method and the Tamura-Nei model. The bootstrap consensus tree inferred from 500 replicates is taken to represent the evolutionary history of the taxa analyzed. Branches corresponding to partitions reproduced in less than 50% bootstrap replicates are collapsed. The percentage of replicate trees in which the associated taxa clustered together in the bootstrap test (500 replicates) are shown next to the branches.

Supplementary Sequences

>Hexosyltransferase_TRINITY_DN28781_c0_g1 len=854
GCTGGGGTTAGGGTACCAGAAGAATACAAATATCGAGAGTGTAAAGAAGGCTGCTGTAATACATTACAATG
GCCAGTCAAAACCATGGTTACAGATAGGTTATGAGCATCTACGACCTTTTTGGTCAAAATACGTCAACTACT
CCAATGAATTTGTCAAGAATTGTCACATCCTGGAATAGTGATTTTTCAATGTACAAGTGCATATACGATAGG
ATGGAGCCTGGATATGGAGCGAAGTTATATGAGGTCCAAGTATTTTTGTCGATACAAATCATCAATATAGG
AGCAAGAAATCAGCCAGATGGAAATACAACTCTTCGAATGGCTCATCTTCTGATTAATGCGTCCCTGGCACC
TGTGTAACAAGAGTCATTCTTTTGGTATGTAACACAATACTTTCTTTAGACGATTTTCAGCTTACCCCATAAG
GGGATCCCTATAGTTTAGCCAATAGAAATCATGGGCAGGATGAAGAATGCCACCTGTCGCTTGTACGAGCC
TAAATGCACCAGAGAAGAAAGAGACCCTCTAAACTCTAAAGGCTCATGTTTAGGCTAATTAGCTTAAGAAC
TATATGATTCATTCAGATTTTGGACTTGATCAGTCATTAAAGCTCTACCCATTTTGTTTTCCTTTATAAGGTTT
TACACAAGTATGTCCTAGTTTGGGCAACAGATGGGTGGTATTTTTTGCTACAGGAATTTTATTCTTTGATAAC
ATTTGCCAAGTATTTGTATTCATGAGAAACATTATAATGTGCAGTAAATTCTTGAAAATTCAGTTAAAAAAA
AAAGAAGGATGTGTTTGTGGGTTGGCCCAATCAGGCCTAACCCATTTAAGACCAGTTTAAAG

>M10_ABC_hairy_fleabane len=1,505
ACGAAGCCTAATGAAGTCAATATATTACCGTTCAAAAAACAAGAACTAACTTGCATATATATGATACATAC
ATACATCTTGCAACTGCTGCTGTATAAAACAACACAAAACATACTTACATTATCAAGCAAATAGTAGAAAC
CATGATTAAATCAAGTGTGATCATCTATCTCTTCTGGAAATTGAAGGCTCTAATGCAATATGCAAATATACA
TGCAAAAAGTATAACTAGGCCGATATTAACTCCAGCGACAACGCCAAGGAAATCATGTTTGTAACCATAAT
ACCGATCCAAGTAACCTCTAACGGTCTCTCCATTCACTAGCTCATCTTCGAATTCCCCAAACTGTGATGCAA
CCATGCCGTAAATGCTCCAAGCTAGAGGGTTTCCCCAGAAGTACCATCTCCACCAAACTGGAATTCTGGGTC
GTGGGATAAGGAATCCTGAAAAGACGTTGAAAATTCCATAGAAAAAGGCAGCAACAATGGCGCCAAAGTT
GGCATTTGGTGTAATGGCGACGGTCATCATGCCATAATATGTCATGTAGAGTAGAGAGCAGAACTGGAAGA
AACAGTACCATAGGAACTTGGCTGCTGTCCAATCAAACCCGATCATAGCATAAACTATTACACTATATGCTC
CAGTTTGGGCCATAACGTAAGGAACTTCGACTAGAACCTGAGCCAAAGCATATGGCAAAGCAGAATACATT
CCTGCAGCCCGTTCTCTATAAAACACGGTGCGTTCTATATCCACAACTGGCTGCACTGATGATGCGTTTTGG
ATAGCGAGAAAGAGGACTGCAGCATACATTGACCCCATTGCATTGGTCAAATCACGTTGGGTTTTCATTTTA
CCACCGAGATCCCAAAACATAGTACCAAAGATAATGCCGATGAAGGTGGTGAAGACAAAACGGACTGCTG
TGTATGGAGGGTTTCTCCAATATGAATAACGTTGTTTCCATAGGCAAGCTAAGCATTGTACCATAAATGATT
GTGAGTATTGTGTCGGGAAATAGAGATCTTGCGTGCCAGCACGTGGTACACTCAGTTCAGCAATCAGTGCTT
TGTTTCTCCTGTAAAGATCTGAATTCCTGTAGATTTCAGTGAAATCTACCCCTAAAGCAATTTCTTGCGACGA
CGTACTAACTTCCAACATCCATGTTGCAGGGTTGTATCCGTCTTTGATCTTACTTATCCCATGAATATCCTCA
AAATACTTGATCAGATCGCAGGAATTGCGACCAACAGGTCCAACATACAACTCTTGCCCTCCTCGTTTCATC
AAGAACAACTCATCAAAAGCTTCAAAGATATCAATACTAGGTTGATGAATGGTGCATACAACTGTTCTTCCA
GTGTCCACAGTGTTCCGAACCGTTCTCATTACAATAGCAGCAGCTCGAGCATCAAGCCCTGAAGTTGGCTCA
TCCATGAAGATTATGGATGGATTGGCTACAAGCTCCCGGCCGTAATGGCCACTCGCGTTGATACCACTGCTT

>M11_ABC_hairy_fleabane len=2,120
GCTCTTGCACCATTAATAGTTTTGGCACAAGTATTGTTCCAAGTACTTCAAATTGGAAGTAATTATTGGATG
GCTTGGGCATCTCCAGTGTCTGCAAGTGATCCAGCCCCGGTTACAGGCTCAACCCTGATCCTGGTTTATGTA
GTTTTAGCAGCTGGATGTGCATTGTGTATACTCGCAAGAGGTCTGCTTCTTGCAACTGTTGCATATAAAGCA
GCCACTATTCTCTTCCACAAAATGCACTTATCCATTTTCCGTTCGCCCATGTCTTTCTTTGACTCTACTCCAAG
TGGACGAATACTAAATAGAGCCTCTACAGACCAAAGTGCGGTGGACATGCAAATTCCATACCAAGTTGGAT
CATTTGTATTCGCTATCATTCAACTTCTTGGCATCATTGCAGTTATGTCACAATGTGCTTGGCAAGTGATCAT
AATATTTATTCCTGTCGGTGGAATGTGCATCTGGTTGCAGCAATATTACCTGCCTTCAGCACGAGAAATGGC
ACGGCTAGTTGGCGTTTGTAAAGGTCCAGTGATACAGAATTTTGCTGAAACAATATCAGGGTCAACAACCA
TTAGAAGTTTTGATCAACAAGGCAGATTCCAGGACACAAACCTGAAATTGAATGATGATTTCGCAAGGCCA
AAATTTCATGCTGCTGCGGCTATGGAATGGTTAGGCATACGTTTGGATATGCTGTCTTCTTTTACCTTTGCTG
CATTTCTAATTTTCTTAATTTCTATCCCAGAAGGAACTATAGATCCAAGTATCGCGGGCTTGGCTGCTACTTA
CGGTCTTACTTTGAACATGTTACAAGGATGGGTAGTATGGACTTTAACCAACCTTGAAAACAAAATTATTTC
TGTTGAAAGGATATTTCAGTATTCATCTATCCCCAGCGAACCTCCTCTAGTTATAGAATCTAATAGGCCTGA
TGATCAGTGGCCGTCACAGGGAGAAGTTGATATCCGTAACCTGCAGGTTCGGTATGCACCACATATGCCACT
TGTGTTGCGAGGCCTTACGTGCAATTTCAAAGGGGGAAAGAAAACTGGGATTGTGGGAAGAACTGGTAGTG
GGAAGTCGACTCTGATACAAACACTCTTCCGCCTTGTTGAGCCAGCTTCTGGACAAATTTCAATAGATGGAA
TTAATATATCCACAATAGGACTTCATGATCTTCGTTCAAGATTGAGTATAATTCCTCAAGATCCAACCATGTT
TGAAGGAACTATTCGAAGCAATCTGGATCCACTTGAAGAGTATACAGATGATAAAATTTGGGAGGCTCTCG
ATAAGTGTCAACTTGGAGATGAAGTAAGGAGCAAGAAAGGGAAGCTCGATTCACCAGTTACCGAGAATGG
AGAAAACTGGAGCGTGGGTCAAAGGCAGCTGGTGTGTCTCGGGCGTGTACTACTCAAAAAAAGCAAAGTCC
TGGTACTTGATGAAGCCACTGCATCAGTCGACACGGCAACTGATGGAATGATTCAGCAAACGCTCACGAAA
CATTTTACAGATTCAACTGTGATAATGATTGCGCATCGTATCACCTCTGTACTTGATAGTGACATGGTTTTAG
TTCTAGAACAAGGTCTGATTGATGAATATGATTCTCCAACAAAGTTGCTGGAAGACAAATCATCTTCATTTG
CTAAGCTTGTTGCCGAGTATAGTATGAGATCGAGTTCCAGTTATGAAAACTTAGCAACAGCTTAGTATGTTG
GTGTTAAGATTGGTGCTTGATGATCTGATCTTGATTGCTCAAATGAGAATATAGACATAGAAAGGTAAGTTT
GTTTTGATTGTCGGCAGTTTGAGCTCTCATGGACTGCATCTAGCTTGTCGCAAGGAGCTTGGTGCCACAGGA
TCGTACTCTATGTACTACCATAACCTTGGCATATCTCCACTGTTTTCTAAATTTTTGATGTGTGTAAGCATAG
CTATTTTCCTCTTGTAAGTTGTCTAAAACTATCTAAAACACTGCTTAAACTTAAAGATCATTTGTTGATCAAG
CTTCTAGTTATTGATTATGTTGTATTCCTCTTTGCGTATCATATATATCATGCGGTTTTAGGTGGATTATGAG
AAAATGAATCAAGAAAGTTCTTCATTCCATC

>ABC_1_TRINITY_DN38339_c0_g1 len=2,750
ATACATAGTTCCATGCTAGATTAAAGATGATTAGTCGTTATTATTAGAAGAAAAATCGGACATCTCAAGTTC
ATCATTATACTAACAACGGCTGCCGCGCATATGTCCTCTTTTGTGTTTTTTAAGGTTAAATTTGCTTGTTGGC
AGGTATCTTGATTGCATCAATAGCCCAAGGATAATCTAGGTAGAATGGCGCTCCGGAATTCTTAATGGCAG
GCGACTTGAAGATAGCATTAGCAAACTTCCCATCAATCATAGCATCAAGCACCAGGTTCATGAGGCTTTTGA
TCGACAATTGGTTAGCAGCATTACATTGAAGATGCTTTAGCAAAACTTGAAAATCTGGAAGACTCTTCTTGG
ACTAGAATTTGAGGGTTCCCTTGCTCCACCAAGACTCCTTGATCAAGAATCAGTACATTGTCCATGTCTAGA
ATTGTAGAGATGCGATGAGCAATTGTGATCACCGTCAGCCCTCTGCATTCACTAGATATGGCATCCTTCAGT
TTCGAAGCAGTTTGAGTGTCTATATTTGCTGTGCATTCGTCCAAACATAACACCATAGTAGACTTAAGAAAG
GCACGTGCAAGACAGATAAGTTGTCGTTGGCCGACTGAAAAAGAAGTTCCAGACTCTTTCATTTGCATATCC
AATCCCCCTGCAGCTCGAACCTCCTCTTCAATATGACATTTCTCTAGAGCTTTCCACATTATATGGTCATCGC
TCAAGTTTAAGGGGTCTAAATTTTCCCTTAAAGTTCCTTTAAAAAGGAATGGAGTCTGAGGAACGACCGCTA
AACGTGACCTAAGATCTCTTAGAGAAATGTCCGCTATGTTTATGCCATCAACTATAATATGTCCTTGACATA
TTGGATTAAGGCGGAATAAAGCATTCAAAATGCTAGATTTTCCAGCACCCGTCCTTCCAACAATTCCTACCT

GTGTTCCTCCAGCTATAGTCAAACTTAGATTACGCAATGCAGGAGGCAAAGATGGCATATATCTGAGTGCTA
CATTTTGAAATTCAATCTGCCCATGAGATGGCCAAGCTGCATCCAAAATCTTGTTCCCTTGAAATTTTTCTTG
TGGAATGTCCATGTACTGCAGAACCCTCTCAACCGAGACCAATTCCTTCTCAGTCTCGGTGAAACTTGTCAA
AAAACTCCCCAACAATGATACAACTGGAGCTGCATATGACAGGGCTAATCCAACCAGCCCTGGGGTACCTA
AATTGACAGGAAGATATCCGTGAGATCCAACAACCGCCATCACCGCGACATATGAGACAACAAAAGCTGCT
AAGAACTGCAGGCGTAAAGATAGCCACAGGCTTGCAATTAACTCTGAATACGAAGTTCTCTGATACATCTT
GACATGGTCCATGAATACAGTCATGAAAAAGTACTCGGATTTGAACGCCCTTATAGTTGATGACCCGTCAA
GTATCTCGGAGAATGATGCATATATGGGTGATCGTGATACGCTGTCTAGCCTTCTCAGTTCTCTTGATGTTGA
CCTGTAGTAGAACTGCACTTTGCTATAGATATACCAAAAAGGCAATATGAGAAGCAAGAACATGACCTGCA
CAAAGGACAGAATTAAAGCAATTCCTAAGAGGCCGACAAAATTGGCAAGTAGAATATTGAGAATGAAAGG
TAGAGAATCATCAATTGTGTAGAGATCAGACGACAGCCTGTTCAGTATTCTTCCACTTGGGGTCTGGTCAAA
AAATGCAACGGGTGCATCAACAAGCTTTGTCAGTAGGTCACTGTGCACCTGAACTGCTGCGCATAGGCCGC
CATATGCGAATGAGAATGCCCTAACTAGAGTAAGAAAAGAGTTCGCCACACAAAAGATACAGAGTATAAC
CAGATAAAAGGATGTTGGATGCTGTTCTAATGCTGATGTATCAACCCAATAAGACAGCCATAGATCATTTCC
ATTTCGAGAAGCTTGCATTAGAATTGCCGACAGGCATGTTATGGCTGTAATATACCAACCAGAGAATGCAG
CATAATTCATGTACACAGTTGTTTCAACTCTGCCTTCTTTTCTCTCTTCAACCCCGATAATATTGTCAGTGGT
ATCCATAATAACTATACCTTCACTCTTTAACATGACACTCTCCTCAACTTCCATACAAGTATCTCCACTTTCT
CCTTGTGATTTGGTCTCAGAAGATGCGTTAAACTCTTCAGTTCTAAATAATGATGCGTATGAAGAAGTTGAA
CATTCAGTTGGACTCCCTACCCATTGCACTTGTCCTTTGTCCATTATAACAACCATATCTGCAGCGAATATTG
CCTGAATATTATGGGTGCAAAGAATGCATGTTTTCTGATTCATAAGAGGACTAAGGAGTGCATGTGATAATA
TCCAAGAAGCCACTTGTGCATCAACTGCACTAAGGACATCATCCAGCATGACTAAGTCAGATCCATGGTAA
ACAGCCCTGGCAAGTGCAAGACGAGCTCTCTGTCCACCTGAAAGATTAATCCCTTTCTCTCCAATATAAGCC
ATATCACCTCCAACCATGAGGGACACATCTGTGTCAAGAGCACATGCCTGTAGAACAGCAGAATATCTTGT
CTGATCATAGTCTTTTCCAAATATGACATTATCACGGATAGTGCCAGAAAGAATCCACGGAATCTGTGGGAC
ATAGGCCACAGACCCACTTGAATATATAGATCCCTTTATAAGATTCAATTCTCCAAGGATAGAATTCAATAA
GGATGATTTACCTGAACCAAC

>ABC_2_TRINITY_DN41040_c1_g2 len=2,435
CATGAAGGTTATTAAGTTACAATCTTGGGAAGAAAAGTTCCAAAGAATTGTAGAATCTTATAGAGAAACCG
AATTCCATTGGTTACATGAAGTTCAGTTTAAGAAGGTTTACAGCACAATATTGTATTGGACGTCACCAACAC
TCGTTTCTTCAGCCATACTTTTTGGATGTGTGTTTTTGTCAAGTGCTCCTTTAGATGCTGCCACTATATTTACC
ATTTTAGCAACACTAAGAACTATGTCTGAACCTGTGAGGTTTTTCCCTGATGCACTCTCCGCATTAATCCAG
GTTAAAGTATCATTGGATAGGATTAATTCTATCTTAGTTGATGATGAACTACAAGATAACAGTATTAAAGAT
CAAGAACTTGAAAAGTCTCATGGTTCTATCAGAATACAGGATGGAACTTTCGCTTGGGATCCTGAATCGCCT
GCTCCAACACTGAAAAATGTAAATCTTGAAGTAAAATGTGGGCATAAAGTTGCAGTTTGCGGGTCTGTGGG
TGCTGGAAAATCATCTCTGCTATATGCTATACTCGGAGAAATATCCAAAACTTCAGGAACTGCGGGTGTTTC
TGGAACAATTGCCTATGTAAGTCAAACATCTTGGATTCAGAGTGGGACGATTCAAGATAACATACTGTATG
GAAAGCCGATGGAGAGAACTAAGTATGAAAAGGCCATCATGGCTTGTGCTTTAGATAAGGATATCGAAACT
TTCAACTATGGAGACCTAACAGAAATAGGCCAAAGAGGGCTTAACTTGAGTGGGGGACAGAAGCAGAGGA
TTCAGCTCGCTAGAGCAGTTTATAGCGACGCTGATATATATCTTCTTGATGACCCTTTCAGTGCTGTTGATGT
GCATACTGCAGCAACTCTTTTTAATGACTGTGTTATGACTTGTCTTGAGGGGAAAACAGTCATTCTTGTAACT
CATCAAGTGGAATTTCTCCCATTGGT
TGATGACATTCTGTTTTCCATCTTTAGGTTATGAAAGATGGTCAAATAATACAATCAGGGAGCTATGAGGAT
CTCATGAAGGTTGGGGCAGCTTTTGAACAGCTAGTGAATGCTCATAAAGATGCCATTACAGGTTTAGAGCG
ATCATCTCATGAAAATACAAGCAGACTCCATAAACAAGTAAATATGCATCAAAGTGTGGAAGACAAAAATA
TAAGTTCTATCAGTAAAGAAGACAATGTGAAGATATCAGGCGTGCAGTTGACCGAAGAAGAAGAGAAGGA
GATTGGGAATGTTGGATGGAAGAGTTTCTTAGATTATGTCATCGTCTCACAAGGAACATGGCACTTTTCGTT
GACTTTACTTGCTACTTTTGGTTTCGTTGGTCTTCAGGCAGCAGCAAGTTATTGGCTGGCATTCGGTATTCAA
ATCCCTGAAATAACCACCACCCTTTTGATTAGCGTTTACACCTTGGCTGCAGCTACAAGTATCTTCTTTGTGT
ATCTTAGATCAGTTTCTGTCACCCTTTTGGGATTAAAAGCGTCCAAATCATTCTTCAATAAATTTACCGCTTC
AATTTTCAGCGCTCCCATGGTCTTCTTTGACTCTACTCCAGTTGGGAGAATTCTGACAAGGGCCTCCTCAGAT
CTTAATGTAATAGATTTTGACATACCTTTTTCATTTGCCTTTGTGGTTGCTGGTAGTATTGAAGTGCTTGGCA
CAATTGGTGTTATGGCCTCAGTGACCTGGCAAGTTCTCATTGTAGTCATCTTAGCCACTATCGCTACAAAAT
ATGCTCAGGGATATTATCAGCCGACTGCAAGGGAACTAATGAGAATCAACGGGACCACGAAAGCACCTGTA
ATGAACTACACATCTGAAACATCACTTGGAGTAGCTACGATACGAGCGTTCAAGATGCAAGACAGATTCTT
CAAGAACTATCTTAAGCTGGTGGATACAGATGCGAGTACTTTCATTTTTTCAAATGCAACATTGGAATGGTT
GATTTTGAGGACAGAAGCATTTTCGAACCTCATCCTTTTCACAGCTGGTCTTCTCTTGGTTTTGATACCAAAA
GGCTTTGTCTCTCCTGGAATGGTTGGCCTCTCTCTTTCTTATGCACTAGTGCTAACAAGTGTTCAAGTGTTCT
TAATTAGATGGTATTGTAGCTTGGCTAATTACATCGTTTCAGTAGAACGGATCAAACAATTTATGCACCTTC
CAACAGAACCACTGGCTATTGTTGACAACAATAGA
CCACCATCGTCTTGGCCTTCCCAGGGAAGGATAGAATTCGAGGAGCTAAAGCTGAGGTATCGTCCATATGC
ACCACTAGTTCTCAAAGGGATCACTTGTACATTCAAAGAAGGAACTAGAGTAGGAATAGTTGGAAGGACAG
GAAGTGGAAAGACAACACTGATAACAGCCTTGTTTCGTTTGGTAGAACCTGATAGTGGAAGAATACTTGTA
GA

>ABC_3_TRINITY_DN27993_c1_g1 len=349
CCGTGACCTTAGATCATGTACCCCTAGAGTAGATATGTTGATGCCATCAATGACTATACTGCCACCAGATGG
CTCCACCAATCGGAACAGAACCTGGATCAAAGTTGACTTCCCACCTCCGGTTCTACCAACTACACCGATCTT
TTCACCTCCGTTAATGCTTAGGGTAATCCCTTTGATCACCAGAGGGGTGTTTGAACGGTATCTAACCTGTAA

ATCCTTGAGCTCTACATTGCCTTGAGAAGGCCAATTTGAAGGAGGGGGGTTATCTTTTTAACCCATTCCGC
TTCTGAAGGTATGTTGGTGAACTGTTTGATCCTTTCAACAGAAACCATTCGGTTTTCCACA

>ABC_4_TRINITY_DN37018_c0_g1 len=4,060
CTGTAGTTGTGAATTTGAGTTATTATACATAAAAGATAATAAAAAACTAACAAAAGTCACCATTCAGCTTGT
GACATGTGGCATGGGTTGGAAATAAGTCCAGGTCGTGGGCAGGGGAGGTTATGCTGCCTGAATATAAGTTG
CAATTGCATCATACGTAACAAATAGTGAGATACAAAGAAAATCTGTTAACCTTATTTGTTTTAGACATTTTT
GTGCTCTACTAAAAAGTATAGCTATTACGAGCTATGATCATATAATCGTCACATAGTACACGTAGGTCTGGT
GTAGACTTTGAGCCAAGTAACCACTGTCAAAACCAAAAAGTGACGATACTTTGTTGATTTTCAGAATCAAG
ATAACGAGAAGGTGAATAGTTGTGTGTATTCACATAAGTTACAATCAATTTCCCTCTGAAAGTTATCCTTTC
TTTTCGTGTTGCACATTGTTACCATCTTTAGTTTCAATAGCTTCGGGATTTATATCAAGAACAATTTGCCCTG
CAGGCGTTGATTCAGATGATGTCAGCAATCCAATTTGTTTCTGCAGGCTCCCTGTGAACTTGTTAATTCCTTC
TCCACCAAGTGCAATGTCTTGTAGATACTGAGCATTTGCAGCGCCTGTACTTTGAACCATCTTCAAAAATGC
CCCTTCATTTGAAATAAGTCTTTCTGGGGTATCATATTCAAGAATTTGACCGTTCTCAAGCACAAGAATTCG
GTTGCAATCAATAATGGTATTGAGTCGATGAGCAATAATGAGCATTGTGCATGATTTGAATTCTTTTCTTATT
GTTTTTTGTATCAGGTCATCCGTTCTAACATCGACGGCTGCAGTTGCTTCGTCAAGAACAAGAATCTTTGATT
TCCTCAGCAATGCCCTGGCAAGACTTAGTAGTTGTCTCTGTCCAACACTAAAATTCTCCCCTCCCTCTGAGAC
CTCAGCATCCAAACCCAAGGGATTCCGCGCGATCACGTTCTTTAAATATGAGCGTTCAAGAGCCTCCCAAAG
ATCAACATCACTATGTTCGTTAAACGGGTCAAGATTAAAACGCACCGTACCTGAAAAGAGAACCGGCGATT
GTGGTATAATCCCAAGAACTCTGCGCAAATCAGTCAATCCAAAACTTGAAATATCATAATTATCAATCAAA
ATTCTTCCTTTTTCTAGCTCAACCATCCTGAACATGCATTGATCATAGTAGATTTTCCTGCACCCGTCCTTCC
AACTATTCCAACTTTTTCGGTAGGAGAAATGCTGAAAGATAAGCCATGCAAAACTGGTGGAAGACCTGGTC
TATACCGAAACTCTATGTTTTCAAATTCTACTAAACCCAACGAGGGCCATCCAGGTGGTGGACGGTTATTCT
CAATTATAGACGGAGCCTCAGATGGTAAATCTATATATATGCCCACTCGCTCAACTGCATTAAAACTATTTT
CTGCTGTACTTGCTTGTCTCAAAACATTACTCATAAGATTTGTGATATTTAGAGAATAAGTGAGGAGCAACC
CAATTGTGGATGCAAAAGCTACTTGATCATTGGTTCTAGAAAATTGCATAACAGCGAAGGTAGCAGTTAAC
CAAATCATTAGTCCTCCTAATGTTTCTAGTCTTATGGTTAGCCAACGGTTAGAACTGAGGTTCAAAAGGGTG
AACCGAATATTATTATCCATAGATTTCCCATTAATTTTTGCCATACGATCATATGCTTTGTATGCACGAATAC
TTGATAAACCATTCAATGCCTCACCAAATTGAGCGTACACAGGAGATCGGGTAATGGAGTCCAAACGCTTG
ACTTCCCGTGATGTGCTCTGATAATAAAGGTAGGCTGTGTAAAACACAATAAGAAGAGGCATTATGGCCCA
CAAAGATATTGTGCTTACTATTCCAATTAGTACAAAAGTCGAAAGTAATTGCCAAACTTGTGTCATAAGATT
ATTGACAAGATTAGCAACATTACGATCAATATCACCAAGATCATTTGCAAACCTATTGATCATCCGTCCAAG
TGGATTTGTTTGAAAAAAAGATGTAGGAGCTCTAAGAACTGAATGCAGCATTGTATCATGGAGTCTTTTAGC
TGCACGGAGACTAGCCGATATCAACCAGAATGAATTTAGAAAAGTTACAAGTACTTGTCCGACTGACAAGA
GGGTGTATATAAGAAGGTAGAACCCAGGTCCTGGAGTTGTTGTATTGCTTTGTTCTGTCCA
GTAACTTAACCATGTGCTACTCGAAATTCGTAGAACTTCAGTTGAAATATAGCACATAAATAATACCAGGAC
AACCCACAGGCCTCCTAAAGCATCCGCGTACCTTGCTAAAACATTCCAGCTAACAATGCCCGTTTGCCTATC
TTCTTGGTTGACAAGAACAGATTTACGCTTCTTTTTTGTTGTGTTGCTTTGATCATTTATAGACTCACTGATCA
TCTCATCAACATACTGTTTTGAGGATCCATTGTTGCTATTGGATTTGGATTCATTTCCATCCACATGATCATC
CATTCCACCCACCTTTTCCATTAATTTTTTAAATAAAAACCCATTTTCTGATAGTTCCTCATAGGTTCCCTCCT
CTTTTATCATACCTTCTGAGAGTAAAATAATCTTATCCACCTGATGGAGAAAATGTAGCTGGTTTGTAACAA
GCACTCTTGTTTTACCTCGCAACTCTTCCTTGATGCAGTTTTCAAAAACCTCTCGACCAACATGAGTATCTAG
AGCACTCAACGGGTCATCAAATATGTAGACATCTGAGTTTGAGTAGACTGCTCTAGCCATAGAGACTCTTTG
CTTTTGGCCACCACTGATATTCACACCTCTTTCACCGATCTCAGTGAGATCACGACCTGGCAAAATATCAAA
ATCGCGCTGCAAAGCAGTTACTTCCACAGTTTTCCAATATTTTGAAGCTTCAAACTTTGATCCAAATAATAT
GTTTTCACGTACTGTAGCATTGAAAATCCATGAAATCTGAGGGACATATGCAGTGGTTCCTCTGATAATAGC
ACTGGAATTTTCCAAAGGAGGTAGCTCACCGAGCATTGCTGAAATAAGGGATGTTTTTCCTTCTCCTGTACC
ACCTACAATTGCAACTAAGCTGCCCACTGGTATATCTATGTTTATATCTGTTAAAGTTGGTTTTGAGGCCTTT
GGATCCCATGAAAAGTAGCCATTTTTTATTGAGATTGCTGGAATCCCTGGTTCAAGAGGTGGATTGGGTACG
AGGATCCTCTCTTCAGCTAAGAATAGTTCTTCCAAGCGCTGTAAGGACACATATGCATTTGCTGCCTGAGTT
ATTATATTAGGAAGTCTGCTTAAAGGAGTTCGCAGCACGGTAAATAAAGAAAGTGATGTGAAAGCCCTTGA
AGGTGTCAAATCTCCACCAAGCAATGTGAAGAATCCAAATGATATCACCGCCACAAGAACTGGAAGACTGT
TCAGTATGAAATTGTTGCATGCTTGCAAGAA
CTGGGCCTTCCATAACCATAGAAGCTCATCTTTTCTGATAGTTTGAATTTTAGATTGGAAACTTTGCTCCCAA
GCATAACATTTCACAGTATCTACCGCAACCAAAATTTCAGTCATTAGTCCAACTCTTTTGTCAGTGTACTGTA
AGCCTGCCTTCGATAATTTCCGCATTTTGCTGACAATCATTGTCTGTAGAGGAAACATGAGAACCAAAATCA
GAGAACCCACAAGTGAAGCAACCCCTAGTTGTTGGTAAAGAAGAATCATGGAAAGCACAATTCTGAATGGA
GCTGACCATAAGCCATGAAGTTGATTGCATATTTGCTGGAGTGCATTAGCATCTGTTGTTATCATATTTGTAA
TTTTCCCCGATTGGAAATTCTTTCGTGCTTCATGGGTTAGCCTTAAGGATTTCCGAAATATAGCAGCCACCAA
AATTGACCTTATCCTGAACCCAACCCTTGCAACATTCTGAAAGTATTGAGCTTCACAGAGAACTCCTAATGA
CACACCAACAAATATTGAGAAGGCATATATGTATCCAATCCATGAAGGATCCCCTCGTTGCAATGACTGCA
GG

>ABC_5_TRINITY_DN25809_c0_g1 len=707
TCCATCAATCATATTATTGAAACATATGTTATGCCAGTATTAGTTGCTTATATTGATCATGTAATCACTGAAC
TGACTTCCTTTCACAACCAGACCAATATTCGGCTACAAGCTTGGAGAAGAAAGAATCGGTCTCCATTAGCTT
TGAAGGCTCATCGTACTCCACCATTTCCCCTGCAACTCAACGACACGAGCTTATTTTTTACTTCATTTTAACA
TGAATGAAAATGTTTAATTATAACAAAATAATGTAGCCGGGGTACATACCATAAGACAGAACCATAACTTT
GTCGTTGTCGATAACCGTTGGGACTCTGTGAGCTACGGTTATGACAGTACAACTAGAAAATTCTGCTCTTAT

GATTCTTTGTAGTATGGCATCTGTGGTTGAATCAATCGATGCAGTTGCTTCATCGAGTACTAGGATTTTGTTT
TTCCTAAGCAATACTCGTCCAAGACAAAACAATTGTCTTTGCCCGGCACTCCAGTTATCCCCTTCATCGCTCA
CTGAAGAGTCTAGCAATTTTGGAAGACTTGCAATAGTACTTTTAAGCTGGCACTTTTCTAATGCCTTCCATAT
ATCGTCATCAGAATAAATTCCAAGAGGGTCCATGTTTGTTCGAATACTGCCCCTAAAAAGGGTTGGTTCTTG
GGGGATGACACTTAGTTTCATCCTGAGATCTTTAAGGCGTATAAGGCAAATGTCG

>ABC_6_TRINITY_DN38603_c0_g1 len=1,248
GCTGGGAGTGGGTTTGGGGTAGCTCAGTTTTTGCTCTATGCTTCATATGCACTCGGTCTTTGGTACGCGTCAT
GGCTAGTAAAACATGGGATCTCTGATTTCTCGAAGACTATTCGGGTGTTCATGGTTCTTATGGTCTCAGCCA
ATGGTGCAGCTGAAACCCTCACTCTAGCTCCTGATTTTATTAAGGGCGGTCGGGCCATGAGGTCCGTGTTCG
AGCTCCTTGATAGGAAAACCGAGATTGAGGCCGACGATGCTGACTCGGCCCCTGCACCTGACCGCCTCCGA
GGTGAAGTCGAACTCAAACATGTCGATTTCGCTTATCCCTCTCGTCCTGACACCCCGGTCTTCTTTGACCTTT
GTCTACGAGCCCGGGCTGGAAAGACACTAGCCCTAGTGGGCCCAAGTGGGTGCGGGAAGAGCTCTGTAATC
GCACTCATACAAAGATTCTATGAACCGTCATCAGGACGTGTGATGATTGATGGAAAAGACATCAAAAAGTA
CAACCTTAAGTCACTACGACGTCACATTGCCATAGTCCCACAAGAGCCCTGCCTTTTTGCTACCACAATCTA
CGAAAACATAGCATATGGACACGAGTCAGCAACTGAGGCCGAGATCATAGAAGCCGCCACCCTTGCAAAC
GCTCACAAGTTCATATCATCCTTACCAGAGGGATACAAAACATTTGTAGGCGAAAGAGGGGTGCAGCTCTC
AGGAGGGCAAAAGCAACGGGTTGCCATAGCACGAGCATTTTTAAGAAAAGCCGAGATCATGCTACTGGAC
GAAGCAACAAGTGCACTAGATGCAGAGTCAGAGAAGTGCATTCAAGAGGGGCTTGAACGCGTTTGCTCAGG
AAAAACAACAATAGTAGTTGCACACAGGCTATCAACGATACGAAATGCTCATAGCATTGCAGTCATTGATG
ATGGCAAAGTCGTCGAACAAGGATCACACTCGCATCTATTGAAAAACAATGTAGATGGTTGTTACGCAAAG
ATGATTCAATTACAAAGATTCTCGCACGGGCATGAAGTTGTAAACCTTGCATCGATTGGCTCAAGTTCTTCA
ACCGTGTGATGTAATTCGAGTATTTTTCTTGTTTATTTTTCGATTTTTTGGTATAACTTCAGTATATGTTGCTA
AATCTTAGTTGTCTAGTTTATGCTGTTTGAGGTAAAGTTAAAAAATGACTATTGTACTTATATCAATATATGA
CAAAATTTACAATTCTCTACAAGTATCAAG

>ABC_7_TRINITY_DN41040_c1_g1 len=3,601
CACACATTATTCGAGCCCTTGTTGGTTAATGAAACACAGTATTGTAATACAAGTCAACTAGAAGAGCCTAGT
TTTCTAAGTAAATTAACCTTCTCTTGGGTAAACCAATTGCTTTCCTTAGGATATAGAAAACCATTGGTTCTTG
GAGATATCCCATCTCTAGCACCCGTAGACCAAGCAGCCATCGCCCATGAGAAATTCACGAAGGCATGGCAT
TCCCTTCAAACAGGAAAAACCTCTAATAATACTAACATAGTTCCTAAAGCATTAGCAAAAGTTTATTTTAAA
GAGATGCTTTTATCAGGATTGTGTGTCTTTCTTAGGACCATATCTGTCATCATTAGCCCGTTGCTACTCTATG
CCTTTGTTGATTACTCCAACCGTGAGACTAATGATTTACATCAAGGACTCTTATTAGTTGGTTGCTTAATAGT
AGTCAAGATAATGGAGTCATTGTCCCACAGACAGTTTTTCTTCAACGTAAGAAGAACCGGCATGAGGATGA
GATCGGCTTTGATGGTGGCAGTGTATGAAAAGCAGCTTAAACTTTCAAGTTTAGGAAAGAAAAGGCATTCT
ACAGGGGAAGTTGTAAATTACATAGCGGTAGATGCATACAGAATGGGCGACTTCCCTATGTGGTTTCATGTC
GGTTGGTCATCTTTTGTTCAGTTATTTCTAGCTATCGGTGTCCTGTTCTCCATAGTAGGACTAGGTGTTCTTCC
TGGTCTAGTCCCTCTAATCATATGTGCACTTCTTAACATACCGTTTGCAAAGTCTATGCAAAAGTGTCAGTTA
GAATTCATGGTGGCTCAAGACAAGAGACTAAGATCCACATCTGAGATCCTAAACAACATGAAGGTTATTAA
GTTACAATCTTGGGAAGAAAAGTTCCAAAAACTAGTTGAATCTTGTAGAGACATTGAATTCCATTGGCTGTG
TGAATCTCAGTTCAAGAGGGCTTTTGGCACGGTTTTGTACTGGATGTCACCAACGCTCATTTCTTCATTTGTC
TTTTTTGGATGTGTGCTTCTGAAAAGTGCTCCACTGAATGCAGCCACTATCTTTAGCATATTAGCATCACTAC
GAAGTATGGCTGAACCCGTAAGAGTGTTGCCAGATGCACTTTCTGCATTAATCCAGGTCAAGGTATCCTTCG
ACAGGATCGATTCTTTTTTGGTTAATGATGAACTTAAAGGTCACAGGATGATAAGGAACCAAGAATTTAAA
ACTTCAAACGCCCATATCACAATACAGGATGGA
AATTTTGCCTGGGATCCTGACTCACCCAATCCAACCCTTAAGAACGTAAATCTTGAAGTAAAATGCGGGCAG
AAAGTTGCAGTCTGTGGGTCTGTTGGTGCTGGAAAGTCTTCTATGCTATATGCACTCCTAGGAGAAATATCC
AAAACTTCAGGAACGGTGGATATTTTTGGATCAATTGCGTATGTTAGCCAAACATCTTGGATTCAAAGTGGG
ACGGTTCAAGATAACATACTGTATGGCAAGCCAATGGACAGAACTAAATATGAAATGGCTATTAAGGCGTG
TGCTTTGGATAAGGATATCGAAACTTTTAACCATGGTGATCTAACTGAAATAGGACAAAGAGGGCTTAACA
TGAGTGGTGGACAAAAGCAGAGGATTCAACTCGCTCGAGCAGTCTATAATGATGCTGACATTTATCTTCTAG
ACGACCCTTTCAGTGCGGTAGATGCACATACAGCAGCTACCCTCTTTAATGATTGTGTCATGAGTTGTTTAG
AGAGTAAAACCGTCATTCTTGTCACTCATCAAGTGGAATTTCTCTCGTTGGTTGACAATATTCTGGTTATGCA
AGATGGTCAAGTTACACAATCAGGAAGCTACAAGGATCTTTTGATGGCAGGAACAGCTTTTGAACAACTTG
TGAATGCTCATAAAGATGCCATCACTGGTTTAGAACCTACTGGAAATGTGGATGAAAGCAAAGAAGATACG
GATAAAAGTAACCTAAGTAAAGCAAACAGTGATGGGCAGTTAATGAAGGGCCTACCAGGAGTTCAGCTGA
CTGAAGAAGAAGAGAAAGAGCTTGGAAATGTTGGATGGAAGCCTTTCTTTGATTACGTTGTTATCTCACAA
GGAATATGGTTCTTGTCTTCTTGTGTTCTAAGTCAAAGCGGTTTTGTTGCTCTTCAGGCAGCAGCAAGTTATT
GGCTAGCATTTGGTATTCAAATCCCCCAAATCACCACCATCAAGTTGATTTTTGTTTACACTTTTCTCTCAAC
AATGAGTACTTTATTCGTCTTTGGGAGATCATTTTTTGCTACCCTTTTGGGATTAAGAGCCTCCAAGTCATTC
TTTAATAAGTTCACCAATTCCATTTTTCGTGCACCCATGGTCTTCTTTGACTCTACTCCGGTCGGACGAATTC
TGACAAGGGCCTCATCTGATCTTAGTGTCATTGATTTTGACATACCCTTTTCATTCGCCTATGTATGTGGATC
TGGTATTGAACTACTAGCCATGATTTGCATCATGGCCTCAGTGACCTGGCAAGTTCTCATTGTAGCTATCTTT
GGAATGGTGGCTTCAAAATATGTTCAGGGATATTACCAACCAACTGCAAGGGAACTAATGAGAATCAATGG
AACCACAAAAGCACCTGTGATGAGTTATGCATCTGAGACGTCACTTGGAGTGTCAACAATACGGGCATTCA
AGATGCAAGACAGGTTCTTTAAAGGCTACCTAAGACTGGTGGACACAGATGCAAGCACTTTTCTTTTTTCAA
ATGCAACTTTGGAATGGTTGGTTATGAGAATAGAAGCATTTTCAAACCTGACCTTGTTCACAGCTAGTTTTC
TCCTCGTTTTGTTACCAAAGGGTTCTGTACCATCAGGGTTGGTTGGACTATCTCTTTCTTATGCACTGACGTT
GACGGGTACCCAAGTGTTTATGACTAGATGGTATTGTAGCTTAGCGAATTACATCATTTCGGTAGAACGGAT

CAAACAATTCATGCACATTCCACCAGAGCCACCGGCAATTATAGAGAAAAATAGACCACCATCATGTTGGC
CTTCCAAGGGATGGATACAGTTCCAGAATTTCAAGTTAAGGTATCGTCCAAATGCGCCACTGGTTCTTAAAG
GTATCAGCTGTACATTCAAAGAAGGGACTAGAGTTGGGATCGTTGGAAGGACAGGAAGTGGAAAGACAAC
ACTGATAACAGCCTTGTTTCGTTTGGTAGAACCTGATAGTGGAAGAATACTTGTAGATGGACTAGACATTTG
CTCTATAGGGCTTAAAGATCTTAGGATGAAACTAAGTGTCATCCCCCAAGAACCGACCCTATTTAAGGGTAG
CATTCGAACAAACTTGGACCCTCTTGGACTACATTCCGATGATGAGATATGGAAGGCATTGGAGAAATGTC
AGCTTAAAAGTACAATTACAAGTCTTCCAAATTCTCTAGATTCATCAGTGAGTGACGAAGGAGAGAACTGG
AGTGCCGGACAAAGACAGTTATTCTGTCTTGGAAGAGTATTGCTTAGGAGAAATAAAATCCTGATACTTGAT
GAAGCCACTGCATCCATTGATTCAGCCACTGATGCCATTCTAC

>ABC_8_TRINITY_DN42180_c1_g3 len=312
TGGGAAGTCAACTTTGATCCAGGTTCTGTTCCGATTGGTGGAGCCATCTGGTGGAAGTATAGTCATTGATGG
CATCAACATATCTACTCTAGGGGTACATGATCTAAGGTCACGGTTTGGTATCATTCCTCAAGAGCCAATCCT
TTTTGAAGGCACTGTTAGAAGTAATATCGATCCGGTTGGTGAACATTCTGATGAGGAGATCTGGAGGAGTCT
TGAGCGGTGCCAACTTAATGATGTTGTAGCTGCAAAACCTGGTAAACTAGATTCTGCAGTTGTTGATAATGG
CGATAATTGGAGTGTGGGACAAAG

>ABC_9_TRINITY_DN41654_c6_g1 len=2,466
CCAGATACCACAAAAAAGATAAACACATTTATTGGTGGCGGGAAACATTCCTTCCAATAACAATTAAAAAT
GCTTCAAATCTCCGGCAAAGGCGGTGGCTTTGGCCAGCTCCTAGCTGCCGTCGCGGCCGCTTTATTACTCCG
TCTATTCTCCGGCCCCGGTCCGGCGTACTTGCCGGAAACCGAAAATGAAGACGACGGAGAGATTTCCGATT
CCGGAGCTTCTGTTTCCGATAAAGTTTTTCCGGTGACAATCCGGTGGACTAACATCACGTGCTCTCTCTCCGA
TAAACCTGCAAAATCTGTGAGGTTCTTGCTTAAGAATGTATCCGGAGAGGCGAAACCTGGAAGGCTGCTCG
CAATAATGGGGCCATCAGGATCAGGGAAGACAACTCTTCTTAATGTGTTAGCAGGTCAGACTACGGCATCA
CCTCGGTTACATTTATCAGGCCTGTTGGAGGTTAATGGTCAATCAATGTCCAACAAGTCCTTCAAGTTTGCTT
ATGTCAGACAGGAGGATCTTTTCTTTTCGCAGTTGACTGTTCGAGAGACTTTGGCTCTTGCAGCTGAACTCC
AGTTAAAGGGAACATATTCAAAGGAAGATAGAGATGAATATATAAACAACCTCTTGTTCAAACTAGGCTTG
GTCAGTTGTGCTGATACACGAGTTGGGGATGCAAAAGTTCGTGGAGTAAGCGGCGGCGAGAAGAAACGCTT
ATCAATGGCTTGTGAACTCATTGCTAGTCCATCAGTGATATTTGCTGATGAACCCACAACTGGACTTGATGC
TTTTCAAGCAGAAAAGGTGATGGAAACCCTTCGACAACTTGCACAGGATGGGCATACTGTAATATGCTCAA
TACACCAACCCAGAGGTTCAGTCTATGCAAAGTTTGATGACATAGTGTTGCTGACAGAGGGTGAACTTGTTT
ATGCTGGTCCTGCAGGGGATGAAATATTGACCTACTTCTCTAAATTCGGGTATATTTGCCCAGACCATGTTA
ACCCTGCTGAGTTTCTAGCTGATATGATATCAATCGACTATAGCTCATCTGATAGTGTCGACTCTTCCCGCAA
AAGGATAGATAGTCTCGTGGAGTCATTTTCTCAACTAATTTCATCAATCTTTTATGCATCAACGCTCACCAA
GCCCTTGAATGATCAAACTAACTTAAGAAGAAAATTTACAGCCAAAGGAGTAGGTGGTTGGTGGAGGCAAT
TTTCATTACTTCTTAAACGTGCATGGATGCAGGCTTCCCGTGACGGGCCAACCAATAAAGTACGGACAAGG
ATGTCAATCGCATCAGCTTTGATTTTTGGCTCAGTTTTTTGGAGAATGGGAAGGTCTCAGACATCAATACAG
GATAGAATGGGCTTGCTTCAGGTTGCTGCTATAAACACCGCAATGGCTGCACTTACAAAGACTGTCGGTGTC
TTTCCAAAGGAACGTGCAATAGTTGATAGAGAGCGGGCAAAGGGTTCTTATATGCTAGGACCATATTTGTTA
TCTAAGTTGATTGCGGAGGTCCCTATTGGAGCTGCATTCCCATTATTATTTGGCACTATTCTTTATCCTATGG
CACGTCTTCATCCAACCCTATCAAGATTCGGTAAGTTCAGTGGAATAGTAACTGTGGAGTCATTTGCTGCAT
CTGCTATGGGTTTGACTGTGGGGGCTATGGTTCCAACCACTGAAGCTGCTTTGGCACTGGGGCCTTCTCTCA
TGACAGTTTTCATAGTATTTGGTGGATATTATGTCAATGCAGACAACACCCCAATCATCTTTCGCTGGATTCC
CCGAGCTTCTCTCATAAGATGGGCATTCCAAGGGCTTTGCATCAATGAATTTAAAGGTCTTCAGTTTGATCA
CCAAAATTCCTTTGATATACAGTCAGGAGAACAGGCTCTGGAAAGGCTTTCTTTCGGGGGTAGCAACATATC
CGAGACAGTGATGGCTCAAAGTAGAATTCTTGCGTTTTGGTACTTCACCACATACCTCCTCCTAGAGAAAAA
CAAACCTAAATATCAACCCCTTGAACAACCACCAGTCGACCAAACTGAACCACAATTAGAGCCCGATCAGC
CTGTAGAAAGTGATGACTCCCAGTCCCCGGGCCAATCATTTGAAATCCTCGAGACTCCAACTGTTGACCAAG
GTAAACCCGATCCTCTGCAGCTTGAATCTCCTCCTGCTACTGCCCAACTTAGTCTGTTTGACTTAGACGGCTT
CTGATTGTACATGGGCATACCAAAATATATTGTTTAATTGATCTCATACAGCAGGTTCTATCTAAAGGATAT
GCATGCTGATGGTGTGGAAAAAAATAAGCAACATTGCACCTCATGCAATTACCTATTACCTGTGTTCTAGGT
AAAGCTCAGTAGCAGTTTTAATAAAACGTGTCAGCTTAGAATTTACATGTAAAAAAGATAGAAACAAAGAA
AAAACTTACAATTGTGGCTTCTT

>ABC_10_TRINITY_DN23566_c0_g1 len=648
GGAAAATATAGGTTTGAAAAGAACAAATGCTTGTATATTATATATGTGAAGAGCAATTAAAATATAATTTCT
TTCGCTAACCGAGCTCCTTCAAACTAATGCGTGGCTCAGATTTATTAGTCTAATATCCGTGCTATTGTTCTAA
AAACTCAGACATGGTGAAAGCAATAGTTTATTTACTCACACTGAATATTTGATTAATAAGCCAATAGTGCTC
GAGCTCAATGTTGCTTCCCGAACTGCACCCGAAGTTTTCTACAACTTGACGCATTTCCCACCTTACCTCAGG
AAGCCTCTATTGTGGCATAGTAACATTGTAAAAGCTTTTGCTATCACTGGACTTCATTAACTTTTTGCATGGA
GTTCCCTTTGCAACTCGACCAGTATTCAGCTACAAGCTTTGAGAAGAAAGAATCAGCCTCCATTAGTTTTGA
TGGCTCATCATACTCCACCATTTTTCCAAAAGATAGAACCATGACCATGTCACTATCTATAACAGTTGGAAC
TCTGTGAGCAACGGTTATGACAGTACAACTAGAGAATTCTTCTCTGATGATTCTTTGTAGAATGGCATCAGT
GGCTGAATCAATGGATGCAGTGGCTTCATCAAGTATCAGGATTTTATTTCTCCTAAGCAATACTCTTCCA

>ABC_11_TRINITY_DN42180_c1_g1 len=916
ATGTTGTATCAGCCAAACCTGGGAAACTTGATTCCACAGTTGTTGATAATGGCGATAATTGGAGTGTGGGAC
AAAGGCAACTTCTTTGTCTGGGAAGGGTAATGCTGAAGCACAGTAGACTATTGTTCATGGATGAAGCAACA
GCATCTGTTGACTCACAAACAGATGCTATAATTCAGAAAATCATAAGAGAGGACTTTGCTGACTGTACCATC

GTTAGCATAGCTCATAGGATACCTACTGTCATGGACTGTGATCGGGTTTTAGTCATTGATGCAGGGTATGCA
AAAGAATTTGACAAGCCTTCACGTTTGATTGAGAAGCCCTCACTCTTTGGGGCATTGGTTCAGGAATATGCA
AACAGATCATCAGGTTTATAAACATCCAATGTATGACAACAATGTCACTCTAATAAGTGATCTAAACGTAAT
TTTTTCTAGGTATCGATAGAAGTATGCATTACAAAAGAAAGCTACAAATTATTTAAACTTCTTACTTGATTGT
CAAATCTCTATATATACAAGATTCCCACCAGCATGCTGGTAGAAATGAGAGAAGTCCATTCAGATCCCCAA
AAATGCACGCGATTGTGATTCCCAGTGCTCCTTTGTCAGGCGAATCCTTTCTGCTCTCTCGGTTTTAAGCCTT
TCTTCCCAATATTCAGGACAACTGCACAAGTTTAATAGAAAATCAGGCCCTTTCTGCTCTAGAAGTGACAAA
CTGGGCAAGTCGGGCGGGTTGACTAATGGGTCGATATTTCTCTTTTATGGGTGATGTTCATTCAGGTCAAAT
AGATAATTTAGTTCAGGTCAAACGATAATTTCAATCCAGGTCAGAATAAATCGAGTTTGCTTGACCAGCCAA
CACTTATACTCCATTTGTTATGTTCAAAAATAATTACTGTCTTGATTATCGA

>ABC_12_TRINITY_DN32993_c0_g1 len=3,004
TCCCAGACAAAGAAGTTGCCTTTGTCCCACACTCCAATTATCGCCATTATCAACAACTGTGGAATCAAGTTT
CCCAGGTTTGGCTGATACAACATCATTAAGTTGGCATCGTTCGAGACTCCTCCATATCTCCTCATCTGTATGT
TGACCGATAGGGTCAATGTTGCTTCTCACCGTGCCTTCAAAGAGGATTGGTTCTTGGGGAATGATGCCAAAT
CGGGACCTAAGATCATGAAGTCCAAGTGATGATATATCGATACCATCAATGATAATACTTCCACCAGATGG
CTCGACCAGCCTAAACAGAACCTGAATCAAAGTTGATTTCCCACCACCTGTTCTACCAACAACTCCGATCTT
TTGCCCCCCTTGAATGTTTAGAGTAATCCCTTTAATCACGAGAGGCGTGTTTGGCCGATATCTAACCTGCAA
GTTTCTGAGCTCCAAACTGCCATGGTTGGGCCAATTTGGAGGAGGGGGACTGTCTTTTCTCACCCATTCTGC
TTCTGATGGTATGTTGGTAAATTGTTTTATCCTTTCAACAGAAACCATTCGGTTTTCAACAAAGCAGCTCGTA
TATAAAGCCCAAAACAGCAGGCCGTTAAGTGACAATCCATATGAAAGTGACAATCCAACGTCCTCTGGTTT
AATGACGCTGCTAGGTAATAGGATCATGAAAACGGTGGAAACACAAAGAAACAAGCTTCCAAGAAATTCC
AAGCGGAACCCTAACCACCCGTTCGATGCATTGTTGTGGAAATCCATCCGTAGATTTCCATCTACACGGTCA
ACATTTTCTTGAACAAACCTGTCCTGTTTTTTGAAACACCGGATGGTCATAACCCCTGAGATGCTCTCAGAG
AAGTGGTGAATGACGGGTGCTTTTGTAATTGAGTCGAGTCGCGTTATTTCTCGAGATGTTGCAAGGTAATAG
CCCCGATACCAGAAGTTGAGCCAACCGAGTGGTATTAGTAGGAAAATTGTTGGCCAAGCATATTGACAGGT
TATGATGATCACACTAATCACGGACATGTACAAAACAAGGCTCAGGTGCATCACGAAGGGTAGAAGAGTAT
CGATGTTGGTTTGATCACTTGATGCCCGACTTAGAATTCTTCCTGAAGGTGTGGTGTCAAAGAATGACATTG
GAGCATGTAGGATGCTATGAAGAATTTGTTTAAAGAACTTTTGTGCAGTCACAAGCCCTAAAAACATAGAG
AAAAAAACTCTTCCTATAATCAGGACAAATGCAATGCCTGCAATAAGTGCATACACTCCAATGAACATTGA
AGGGTTGAAAGAATCAGCTCGGTCCTCTGAGGTTTCATAAGCCAGCCAGTAATCACTGGCCATCTGTGTTGC
TTGCCACAAGAACGCCAACACTAAAACCAGAATCATGCCAGTCCACCCAAATGCCTCAGTCAAATACACTT
TGTAGACATGTAAACTGACTCTTCCTGTTTCTCGCTCTTCATCTTCAATCAGTTTTGATGTTCCAGCTATAGA
ATTCGATAGGGAGCGATCTAGAGATTTATTGTTTTCATTGTTGTCTTCCCTGGGATTATCCCTATGAGATTTT
TTCATGGTTTCTGGTTCAGTTGGTTCCATTTGAACTAGCTGCATTGAGGTCTCATGAGCAGACACTAAAGCTT
TAAAATCGAGTCCAGACTGACACAGTTCATCATACTTTCCTGATTGAACGATCATCCCATCGCGCATCACTA
GGATAAGGTCAACGTTATGGAGGAAATCGACTTGGTGAGTCACGAGTAAGACCGTCTTGTCTCTTAGGCCC
CCCCTCACACATTCCTTGAATATTTCTGAACCGGTGTGAGCATCAACAGCACTAAAGATGTCATCAAGAAGA
TAGATGTCGGAATCTTGGTATACAGCTCTTGCGAGCTGAATCCGTTGCTTTTGACCACCGCTTAGATTGATCC
CACGTTCACCGATCTCTGTTTGATCTCCAAATTCCATCATCTCTAGGTCTTTTTCCAAGCAACAGGTTCTTAT
TACATTCTTGTACTTGTGTCGGTCCATAGGCGAACCAAACAGAATGTTTTCTTGAATTGTCGAATTTTGAATC
CATGCTGTTTGTGCGACGTAAGCAGTGGTTCCAGAAACTTTCACCTTTCCGGAAATCTTGTTCATTTCACCGA
TAATAGACGCGAGTAGAGATGATTTCCCTGACCCTACAGTCCCCACAATGGCTGCAAGTTCGCCTTTTTTGA
TCTCAAAATTCAAATTTTTGATCAGATCCCCTTCTGAAGGGTCATCGTGCCAGCTAAATGACCCGTCTTGGA
TCTTGACCGCGGTCATAGTTTCACTATCACTAAATTCGCCCCTCTCGACTGAACCCTCCTCCAATTCCTTACT
GGTCATAAACTCATCTAATCTCCCCAACGAAATCATTGCTTGAGAAAGAGATATCATCGATTGAGGAAAAC
TCCAAATCGGTTCCTGCAAGTTCTTAAACAATGACGTGGCTGTGAACACCGTTCCAGCATCAAGTGGTACCC
CAAGCAAAACCCCGCTTCCGAATGTGACAGTCGAGATAAACAAAGGCGTGCTCCACAGAACAACCATGTTT
CCACCGATCGAGTACATGAATTTACAAAGCCAACCGTATTCTGACTCACGAAACGACTGAATCCTTTCGTTA
AAATGTTCTTCCCAAGCTTGAAACTTAATCACCCTCATATAATTCAGCATCTCATTAGTAGCTTTCATCCTCG
AGTCGCGACTTGTCATAATGTTAAACTGGAACCGATTGTTTCGTTTAGTCCCCACAAACACAAATGCGAGAA
TAGCCAAAACCGCAATCAGTGTAACCACGGCCGATGAGCCAAGGTACATGTAAAGAATGGCCAGTGCTACG
GCTACTTGAAGCGGCATTAGCCAAATGGCGTGTAACTGAAGCATTATATCGGATATTTGTTGAGCATCTACA
GCCATGTAGTTCACAATCTGTCCTACACCGTGAGATTGCCTGGCAGAACACGAA

>ABC_13_TRINITY_DN41851_c0_g1 len=2,853
TAAATTTGAAATGGTTAAGGCAACAAATTGGTTTGGTTAGTCAAGAGCCAGCACTCTTTGCTACAACAATTC
AAGAGAATATACTATTGGGTCGACCTGATGCATCCATTATCGAGATTGAGGAAGCAGCTCGTGTTTCCAACG
CTCACTCGTTCATTATTAAGTTACCCGACGCGTATGATACTCAGGTTGGGGAGAGGGGGTTACAATTATCGG
GTGGACAAAAGCAGAGAATAGCGATTGCGAGGGCAATGTTAAAGAATCCCGCAATCTTGCTTTTGGATGAA
GCAACTAGTGCTTTGGATTCTGAATCTGAAAAGTTGGTTCAAGAGGCGTTGGATCGATTCATGATTGGGAGG
ACAACTCTCGTGATTGCCCACCGTCTCTCCACGATAAGGAAGGCGGACCTGGTAGTGGTTTTGCAGCAGGGT
AGTGTTTTGGAAATGGGAACTCATGATGAGCTTTTTGCAAAGGGCGAAAATGGTGCCTATGCTAAGCTTATT
AAAATGCAAGAGATAGCTCATGAAACTGCCTTGCATAATGCCAGAAAGAGTAGTGCCAGACCATCTAGTGC
AAGGAATTCAGTGAGTTCGCCTATAATTACCCGGAACTCGTCTTATAGTAGATCGCCATATTCCCGAAGATT
ATCAGATTTTTCGACATCAGACTTTAGTCTATCAGTTGAAGGTGGTTACCTGAGTTACCGTCATGAAAAACT
TCAGTTTAAGCAGCAAGCAAGTTCCTTTTGGCGGCTAGCAAAGATGAACTCACCCGAATGGACTTACGCTTT
GATCGGCTCAGTGGGGTCCGTGATTTGTGGGTCACTCAGCGCTTTTTTTGCTTATGTCCTAAGTGCTGTCCTG
AGTGTGTACTACAATCCAGACCATGATTACATGATTAGAGAAATCGGGAAATACTGTTATCTGTTGATTGGG

GTTTCATCTGCTGCACTCATTTTCAACACTTTGCAGCATTTTTTCTGGGATATTGTTGGAGAAAATTTGACTA
AACGGGTGCGTGAGAAAATGCTGGCTGCAGTTTTGAAGAATGAAATGGCATGGTTTGATCAGGAGGAGAAT
GAGAGCTTGAGGGTTGCCGCCAGGCTGTCTCTCGATGCTAATAATGTTCGTTCAGCAATTGGTGACCGTATT
TCAGTGATTATGCAGAACTCTGCCCTCATGTTGGTGGCCTGCACAGCTGGTTTTGTTCTTGAGTGGCGGCTCG
CACTTGTTCTGGTTGCTGTTTTCCCTCTGGTGGTTGCAGCCACTGTCTTGCAGAAAATGTTCATGCAAGGATT
TTCTGGTGACTTAGAAGGTGCTCACTCGAAAGCAACACAGTTAGCGGGAGAAGCGGTGGCCAATATGAGAA
CGGTAGCTGCTTTCAACTCCGAGACAAAAATCGTCAACCTTTTCACACTAAGTCTCGAGAAGCCTCTTCGTA
GATGTTTCTGGAAGGGCCAGATAGCTGGGAGTGGATTTGGAATAGCTCAGTTTTTACTATATGCTTCTTATG
CACTTGGCCTTTGGTATGCGTCTTGGCTTGTTAAGCACGATATCTCTGATTTCTCAAAGACGATACGGGTTTT
CATGGTTCTCATGGTCTCGGCAAATGGCGCAGCTGAAACCCTGACTCTTGCTCCTGATTTCATTAAGGGTGG
CCGAGCAATGAAGTCTGTTTTTGATCTACTTGACCGGAAAACTGAGATCGAGCCCGATGAGGTAAACTCCGT
CCCTGTTCCGGACCGTCTCCGAGGCGAAGTAGAGCTCAAGCACGTTGATTTTTCTTACCCGTCTCGCCCTGA
CGTACCAGTTTTCCGTGACCTCTGTTTACGAGCCCGTGCTGGAAAAACGCTTGCCCTGGTTGGTCCTAGCGG
TTGCGGGAAGAGCTCAGTGGTCTCACTGATTCAAAGATTCTACGAACCATCTTCGGGACGCTTGATGATCGA
CGGAAAAGATATCAGGAAATACAACCTAAAGGCGTTAAGGCGTCATATTGCAGTCGTTCCACAAGAGCCAT
GTCTATTTGCCACCACTATCTATGAGAACATTGCTTATGGACACGAATCAGCAACTGAAGCTGATATAATCG
AAGCAGCCACCCTCGCCAACGCTCACAAGTTTATCTCTGCATTACCTGATGGATACAAAACATGTGTTGGGG
AAAGAGGAGTGCAGCTTTCAGGGGGACAAAAGCAGAGAGTCGCCATAGCAAGAGCGTTTTTAAGAAAAGC
TGAGGTGATGTTACTAGACGAAGCTACAAGCGCACTTGATGTAGAGTCTGAGAAGTGCATTCAAGAGGCTC
TCGAACGAGTCTGCTCAGGGAAAACGACCATTGTTGTAGCTCATAGGCTATCGACTATAAGAAATGCTCAT
GTGATTGCGGTGATAGATGATGGGAAAGTGGCAGAACAAGGTTCACACTCGCACCTGTTGAACAACTACCC
GGATGGGTGTTATGCCAAAATGATACAGTTGCAAAGATTCTCTCACGGGGAGGCTGCAAGTATGGTATCAG
GGGGTTCAGGTTCGACTTCAAAACGTCAGATAGATGGCCAGTGAATTGAAATGGTCACATTTTAATATACG
AAAACTTGAGTTTTGAATACTATTGGTATCATATATCATTTGGTATAGTAATATGAAATCATTAATAGAATA
GAATGGTACTTGTGTATTATTTGGTGCTTTCAACATGTTATATGTCTGGTTCAAAAGGATAACATGGTACATT
ACAATCCAAGTAATTATATATAGGCCTGAAATATGGCTTTAAGATGGTACTAATCGTTCAAATATATTGTAT
AGAATTGATAAGATTGGATCAATAGACACTAGTTTTTGGTCAAAAAAAAAA

>ABC_14_TRINITY_DN37243_c0_g1 len=2,118
GTGAGAAGTTGCACGGTCAATTTTCTCGAGTTCCAAGCTGCAATGGAACCAGGTCACTCCACATAGATGATC
TTGTGCCTGATTCCATGTCTAAAGTTTCTATAGCAAGTGGCGATGAACATCCAGTAGTTCAACAAAAAGGGC
GTTTCAAGGTTACCTCTGAGAGTCTTGACTTAGACAAGGCAGCTACACCTCCCCTGCTGCAGAAGAGTCACA
GTATGCAGGTAATATGCCAAAGTTCCACTGGTGTTACTTCCATGCCGCAATGCCAGTATAGCAATCTTCCTA
TGCCTGCCCTACAGTTTATTTTGCAGACAAATACTCTTCAAAGGGATTGTATTTTGAGTCTAATGAAGGCAA
ACTCCACCGGTGATTCTACAGCTCACGTTGATATAGATGGAGGGTGCTCATCAACAAACATTCCTGCACTAG
ATAAATCCATGATTGATACAAACAGCAACAGGGAAAAGGAATTACTTAATGAAATAATTTACTTACAATGG
AGGCTCACATGTGCACAAGAGGAGCTCCAGAAATGCCGAACTGAAAATTCACAGGTATGACGTTTGTTATA
CCTTCAGGTAACCCCCGCGGCATCAGAAACAGAGAAGTTCTTTTCCCTAAAATTGCCACCCGAGTAAAAGA
TCAAGTTATTCAGGTGTTGATATATAGTATTTTCCTGTTTAGAGATGAGGCATACTTGTAGGTAGGGGGTGA
GAACTAATGTTCCCCCTTCCACTCCTTTATTTTTAGGTGTTCTTTCTTTTGATTTCACTTCTTAACTTATGACT
AGTTAAACATAATTTTACACAGCTTAACTAATTTGTCCATACATTATTGCAATCACGAGTCTACAATTGTAC
ATGAACTGTCAAACCTATATAACCGTCTGCTTATTGACTTAAGCACTAGTGATGCTCAATTTGAAATTGACG
TTCAGGTATGTATGTGTCCCTATATAGTAAGCTCTCACAGCGTTAAGCACTAGGTAAGCTGTCTAAACAGAA
ATAGTAACGTTATGAGAAGACACATGCAAATACAAACAAACATTTATTCACTTTAAGTGGACATTTAGTCGT
GCACATGCATTGTATATTCTGATAACCACAATGATAGGAATTGTGGTGTTGAATCTTGATATAAGTGGACTC
TCAAAACATAAAACATTAAAAACAAATTAAGAGCTAAGTTCAAGAAATCTCCTTGCCATGGGATGTTTAGC
GTCCGAGAGCTCATCAGACCTTAGAACCTCAACAATATCACCATCCACGAGAAGGCAAACCACATCTGCAA
TCCTTTGAATTTGTTTAATGCTGTGAGAGACCATAACAACAGTCATTCCTCTACTCTTCAACTTCACCAACGC
ATCTTCTATGTTTTGGGTTGATATTGGGTCCAAGGCACTCGTTGGCTCATCTAACAACAGAACCTCTGGCTCA
TTGGCTAACGTTCTAGCAAGTGCAACCCGTTGAGCCTGACCAACTGATAATTCGCTGCCTGGCTTGTCGAGA
AAAGATGAATCAAGATCAGCAAAAGTCAACAAGTTGTACACTTCTTGATCTGTTAGTTTCTTTCCTTTCAAC
TGTGGCCCATATCTTATGTTGGTTGCAACTGTACCTTCAAACAAAACGGGAAGCTGAAACAACATTCCAACT
TTTCGCCTTAGATCAAGAACATCAAGCCCAGTGATATCTTTCCCATCCAAGAAAACCGTACCAGAAGGCGGT
TCCCATAAACGATTCAACGCTCTTAATAACGTCGACTTACCACTACCACTTGGCCCAATGATCCCCATGATT
TTGCCTCTGGGTATCTCTAAGTTCACATTTTGAAGGATTGTAATTCCTTTATCCAGTGTTCTTGTCAAACCCTT
TACTTGAATCTTTGCTTCTGATCCATTAATTACCTCATTATTTACTGACAGTAAATGTTCACGCGCACCATCT
TGAAAATTATCCATGAAATTGAAAGAAGTGATCTCAAAGGTGTGTAGAATAAAAGATTTATGGGGTTATGA
AGATGAGCAGAAAGCCTTCCTCAGTAAACAACTTTGTGTTCACTTTGTATGTGATGGATGCCACCTTTTTTTT
TTGTTCTGTCGCATCTAGTGTCTGAGTG

>ABC_15_TRINITY_DN37746_c1_g1 len=1,769
TATATATACATATATATATATATATATATCGAATTGTAAGTAAATTGTAATAATGTTTCACGGTGGTGGTGGTG
GTGGAGGTGGTGGTATGCATGGTTTCAGAGGTGAAAGAACTCCATTGCTCAATAGAAATGGTTTGGCTAAT
GGTGAGCAGGGGGATCTTGAGCTTGGAGATGCAATCCCGGCTGCTAATGTGAGCTATGTTAGAGTGCTTTCT
TTGGCTAAACCGGAGGCTGGAAGGTTGATTATTGGTACTATTGCTTTGTTGATTGCCTCCACGTCAAGTATA
TTGATTCCACGGTTTGGTGGGACGGTTATTGATATTGTTTCGGGAGAACTGGAAACACCTGAACAGAAAAGT
GAAGCGGTTAGTGAAGTCAACAGAACCATACTTGGAATCTTTCTGATTGTCATCATTGGTTCGATCTGTACA
GCACTTCGAGCATGGTTATTTTCTTCCGCAAGTGAAAGAATTGTTGCAGCCTTGAGAAAGAACCTATTCAGT
CACCTCGTTAATCAAGAGATAGCCTTTTTTGATGTTACACGAACAGGGGAGCTCCTTAGCAGGCTTTCAGAG

GATACTCAAATCATTAAAAATGCTGCAACTTCAAATGTTTCTGAGATTCTCAGGAATCTTTCCACTGCATTTA
TTGGTCTAGGTTTCATGTTTGCCACGTCATGGAAGCTAACATTACTGGCTTTGATTGTTGTTCCCGCCATTTC
AATAACCGTACGCCAAGTTGGACGATATCTTCGTGAAGTTTCCCACAAGACTCAAGCTGCTGCAGCCATTGC
TTCCTCAATCGCTGAGGAAACTTTTGGTGCAGTACGCACTGTCAGATCTTTTGCTCAGGAGAACTATGAGAT
TTCACGCTATTCTGAGAAAGTTGATGAGACATTAAAACTTGGACTCCAGTATGCCAAAGTGGTAGGTATCTT
CTTTGGAGGTCTGAACGCAGCATCAACATTATCAGTTATCATAGTAGTGATATATGGAGCAAATTTGACCAT
AGTAGGCTCTATGAGCCCTGGATCTCTTACGTCGTTCATTCTTTATAGCCTGACGGTTGGAACCTCTGTTTCT
GGACTGTCTAGCATTTACACAGTGGCAATGAAAGCTGCAGGAGCCAGTAGAAGAGTTTTTCAACTTCTGGA
CCGTGTTTCTTCGATGCCACAAGCCGGAAAGAAGTGTCCTATGGGGGATGGTGATGCTGAAGTAGAGTTGG
ATGATGTCTGGTTCGCCTATCCTTCACGTCCTCGTCATAATGTGCTCGAGGGAATAAATTTGAAACTGCGGC
CTGGTTCGAAGGTTGCACTTGTTGGTCCTAGTGGTGGTGGAAAGACCACTATAGCCAATCTGATTGAAAGAT
TTTATGATCCTATTAAAGGAAGAATATTGATTAATGGGGTTCCTTTGGTAGACATATCACATGAACATCTAC
ATAAAAGGATTAGCATAGTGAGCCAGGAACCTACACTTTTCAATTGTTCAATTGAAGAAAATATTGCTTATG
GACTCGATGGGAAAGCAAGCGAGGAAGAGGTGGAAAAAGCAGCTAAAATGGCTAATGCTCATGATTTTTAT
ATCTAAGTTCCCTGAAAAATACAAGACGTTTGTTGGTGAACGTGGAGTCAGGCTTTCAGGAGGTCAGAAAC
AGAGAGTAGCGATAGCACGAGCCCTATTGATGAACCCAAAGATTCTTCTTTTGGATGAAGCTACTAGTGCTT
TGGATGCAGAAAGCGAGTACTTGGTTCAGGATGCAATGGAATCT

>ABC_16_TRINITY_DN41980_c0_g1 len=3,082
CTTGTTAGCAGTTTCTAGGTTAACTCTAGATGTACGATTTCTAGTTTCTCATCATGATCACCCACGACTACCC
GATTACATTGACTTTATTGCACTTCTCGCCTCGGCTTCCTTGTTATTTCTTGCAATAAAAGGAAAAACAGACA
TAGCGCTGGCTGACCAAAATCCAATCACCCAACCATTTCTAGACAGAAACCCAAACGCAAAAGACCATTTA
GAACCAAAAAGAGAATGCCCATATGGAAAAGCTTCAGTTTTTCAACTTGTTACGTTTTCTTGGCTTAACCCG
TTGTTTGTGGCTGGAAACAAGAAACCACTTGACCAAGATGAAGTCCCGGATGTTGACACTATAGATTCTGGT
TGTTTTACATCAGAATCATTCGATGAATGCTTGAGGAAAGTTGGAACCGAAAAGCCTTCTATCTACAAAGCA
ATCTATCTTTTCACAAGAAAGAAAATGGCAATCAATGCCCTTTTCGCCATTACAAGTGCTGCAGCATCATAC
GTAGGGCCATATCTTATCAGTGATTTGGTGAATTTTCTGAATGAAAAGAAAAACAGAAGTTTAACAAGCGG
CTATCTTATTGCACTAGGATTCTTGGGTGCAAAAGTCGTGGAGACACTAACACAACGACAATGGATCTTTGG
TGCCCGTCAACTGGGGCTCAGACTTCGGGCAGCTTTGATCTCTCATATATACAAAAAGGGGCTCGTGTTATC
AAGCCGTTCATGCCAAACTCACACAAGTGGTGAAATTATCAACATTATGAGTGTTGATATCCAACGAATAA
CGGATTTCATGTGGTACATTAACACATTCTTTATGTTACCTATTCAAATCACATTAGCAATGGTTATTCTTCA
CATAAATCTTGGAATGGGATCGTTTGTGGGACTAGCAGCAACGATGATATTAATGTCTGGCAACATACCTTT
AACAAGAGTCCAGAAATGGTACCAATCGAAAATTATGGAGTCAAAGGATACACGAATGAAGTCAACGTCA
GAAGTTTTACGCAACATAAAGACACTTAAACTTCAAGCCTGGGATACTCACTATCTTAAAAAACTCGAAGG
TTTCAGGAAGGTTGAGTATGATTGGCTCTGGAGATCACTTAAACTGAACGCTTTTGTAGCATTTGTTTTCTGG
GGTGCACCAACGTTTATCTCTGTGCTGACTTTTGGAGGCTGTGTTTTGATGGGTATCCCACTCACAGCTGGG
AGAGTTCTTTCTGCTTTAGCTACATTTCGGATGTTACAAGATCCTATCTTTAATCTACCTGATTTACTAAACG
TAATTGCACAAGGGAAAGTGTCTGCAGATCGTGTGGCTTCTTACCTCCAAGAAGAAGAGATTCAGACAGAT
ATGGTTGAATTTATTCCTAGAAGTCACACAGAATTTGATGTGACGATTGAAAACGGAAGGTTCAGCTGGGA
CCCAGATTCAAGAACCCCAAATCTTGATCAGATAGAGCTACAAGTGAAAAGGGGAATGAAGGTAGCCATTT
GTGGAACTGTTGGGTCTGGAAAATCGAGTCTTTTATCATGCATTCTTGGTGCGATGCCCAAGTTATCAGGAA
CTGTGAAAATAAGCGGTTCAAAAGCTTATGTTCCTCAGTCTGCATGGATCTTGACAGGGAATATTAGAGAG
AATATATTGTTTGGAAATGCTTATGATCAAATGAGGTATGACAAGACAATTAAAGCATGTGCATTGAGTAA
AGATTTTGAGCTTTTCTCTACTGGAGACCTGACGGAAATTGGGGAAAGAGGGATAAACATGAGTGGAGGCC
AAAAGCAAAGGATACAGATTGCCCGTGCAGTGTATGATGACGCTGATATATATCTCCTCGATGATCCCTTTA
GTGCTGTAGACGCCCACACAGGAACTGAACTCTTTCAGGAATGCCTTCTGGGGATACTCAAAGAGAAGACC
GTACTCTACGTGACACATCAGGTCGAGTTTCTTCCAGCTGCAGACCTCATTCTGGTTATGCAAAATGGAAAA
ATTGCCCAATACGGAACATTTAAGGAACTTTTGAAACAAAACATAGGATTTGAAGTTCTTGTTGGAGCTCAT
AACCAAGCACTAGAGTCGGTGCAAGCAGTTGAGAGTTCAAGCAGAGAAACCGATCCAACAACAATAGAAG
AAGAACCAACTTTGATGACAGAACTAGCACAAAAGCAAGATTCTGAGCATAATCTATGTGTAGAAATCTCA
GGAAAAGAAGGAAAGTTGGTTCATGAAGAAGAAAGAGAAAAAGGAAGCATAGGGAAAGAAATTTATTGGT
CTTATTTGACTTTAGCGAAAGGCGGTGTCCTAGTACCTATTATACTTCTGGCTCAGTCTTCTTTCCAGCTTCT
ACAAATTGCTAGCAATTATTGGATGGCATGGGCGTGTCCTACCGATTCAACTGAGATTGTGACGGGGATGG
GATTCATTTTACTTGTTTATACACTTTTGGCATTTGGAAGTTCACTCTGCATATTAGTTCGTGCCTCACTTGTT
GCTATAGCAGGCCTTCTAACTTCAGAAAAGCTTTTCAGCAATATGTTGCGCAGCATTTTTCGTGCACCTATG
GCATTCTTTGACTCGACACCAACCGGAAGAATCTTGAATCGGGCATCCACGGATCAAAGTGTTATAGATTTA
GAAATGGCAAACCGAATAGGATGGTGTGCGTTTTCAATGATTCAGCTTCTTGGAACGATGGCAGTGATGTC
ACAGGTAGCATGGGAAGTATTCGCCATCCTTGTTCCGGTGACAACCATCTGCGTTTGGTATCAAAGGTATTA
CATACCAACTGCAAGAGAATTGGCACGGTTAGCAGGTATAGAGAGAGCTCCGATCCTTCACCATTTTGCTG
AATCACTTACGGGTGCAGAAACTATTCGAGCTTTCCAACAACAAGACAGATTTATTGAGAAGAATCTTTGTC
TCATTGACAATCACTCAAGACCATGGTTTCATAATGTGGCAGCAATCGAGTGGCTTTGTTTCAGGCATAGCA
GGATTAGCCGTAACTTATGGAATCAATTTGAATGTTCAGCAAGCTGCAGTGATATGGAATATATGCAATGC

>ABC_17_TRINITY_DN29514_c0_g1 len=741
GCGATAGCACGAGCCCTATTGATGAACCCAAAGATTCTTCTTTTGGATGAAGCTACTAGTGCTTTGGATGCA
GAAAGCGAGTACTTGGTTCAGGATGCAATGGAATCTCTAATGAAGGGGAGAACGGTATTAGTGATAGCACA
TAGGCTTTCAACAGTACAAAGTGCAGATACAGTGGCAGTTGTATCAGATGGAAAGATAGTTGAACAGGGGA
CTCATGATGATCTTCTAAGCAGGGACGGTATATACACCGCATTAGTAAAGAGGCAGCTACAGGGGCCTAAA
ACCGAGCTCTAACTATGCTGCAAAGTAATAAGAAATGCTTCGTGATTGGTTGGTTCTGATTTGGTAACTTAT

ATACATACTTTCGGTGTATAGGTGCTCTTGTTTAATCAAGCAAATGACAACACTAAAGCTCACATGCATTTC
TTCATCTTTGCATTTGCATCGTTAAGTACCCTTCTTTTCCGATTACTTAGACTTGGCCTCTTGGGTCTTAAGAA
GATCTAAATTAATCACAGAAGGGCCACAACTACTACGATAGTAGGGCCGTAGGGGTAGTTGGAGCTAGGCG
TCAACCCATATAGTGTAATTTTTTTTTTGAAAAACGTTGTGTTTCTTTTATAGAAGCTAAGGGTCAATGTAAC
TACTTCTAAAATTTTGAAAGATTTGCTTAACAATGAAACTTATAAAGTCTTATTTGTGTAATTGAATAGAAA
ATGGAAATTGTCAAAAAAAAAA

>ABC_18_TRINITY_DN41405_c0_g1 len=5,388
CGAATCACAAACATTTTTCCGTTTTTTTGGAATCTATATCTCTCTCTATGATTTTATTGTTGATGAGTGTGTAT
GAAGGTTGAATCTGGAAATTATGTTGACTAATAATAGTAATAATAATAATAATCTTACAAATATTATTGTGA
GATCTATATTTTGTTGGGTTTTTTACAATAACGTGCTGAGATGTGAGAGTAATATATTTACCAGGTGTAGAG
TTTTTTACCCATTCCCATTCCCATTACATATATTATCTTGGAACTTAAAATAAAAGACAAGCTGAATTGTCTA
CAACCCTGTCAATTTTCAGCCCGTTGGCTAGGTCTTGAAGTAGTTCCGTCCCCATGAGTTGACTTCTTCCCTG
TTATGGTTCAGTTTGCTCAATCCGAGTCCAAATGTCATTAAGTGAGCTACTATTGATACATCAAAAAATTCC
ATGTTTAGAAATGTACCATGAATTTCGCTATGTACATTTTTTTGACTTTTCCCAAGGAAAAGAATAATTGACC
TGGTAACACGAGTATGATATATATTGCTGATCCCGGCCCACAAAAAGGTGGCTGTTGACAGAATACACACG
TACCTTACCTGTACATCTCTCTAAAGCCTTATTTTTCGTATGATAAGATTATACGTAGTCAAATGGATAGCCA
TCTCAAACAAAGCTACATATCTTGAATGATCTTCAATTCAAAGAATGTCGGTAGATTGAGAATGTGACCAAT
ATTCCTTCACAAGCTGTCCAAACAAAGAATCATCTCTTCTCATCAGCTTCACCGGTTCATCATATTCTGCGAT
TTTTCCGTCACTCATAGTTAAAACCATTGTGCAATCCATCACGGTTGGTATCCGATGAGCCACTGTAATTAC
AGTGCAATCAGCAAACTCGGATCTGATGGTTTCTTGTAACATTGTGTCTGTAGCATTGTCGATTGATGCAGT
TGCCTCATCTAGTACCAAAATCTTGCTTCGCCTCAACAATGCACGCCCCAAGCAAAACAACTGGCGCTGCCC
CATGCTCCAATTTGTTCCATCTTCGACTACTATGGAATCCAGCCCTCCTATTTTATCCTGAACGGCCTCTCTG
AGTTGACACTTGCCTAGAACCTCCCATATTTCCTGATCTGCATGTTGACATAGGGGATCCAAATTGTATCTA
ACTGTTCCATTAAAAAGAGTAGGATCTTGAGGTATAACCCCGAAACGTGACCTTAAATCATGAAGTCCAAC
TGTAGAAATATTTATCCCATCAACAAGTATTTTCCCACCAGTGGGTTCCACTAGACGAAATAATGCACCAAT
GAGTGTAGTCTTTCCACTACCCGTCCTCCCAACAATACCAATCTTGTGCCCTCCTTGAAATGTACATGTAACC
CCTCGAAGAACTAGAGGTGCATCAGGTCGGTACCTGATTTGCAAATCCTGAATATCGACTTTACCCCTGCTC
GGCCAGTTAGCAGGGGGGCGATTCTCTTCTATAACTATAGGAGCCTCACTAGGTAGATGCATGTACTGGTTG
ACTCTTTCGACTGAGATGATATTGTTTACCAATGTGCACTGGTTTTGAATCGACATAACCAGTGACATGTTTA
GAGAAAGCCCATAAGATATCGCCATTCCAATAAAACCAGAACTGAAAGTTCCAGGAGGAAGCAAAACCAT
GCAGAGTCCTGAAGCTGATAAAACGGTGGCACTGATAGTTTCAAGTCGTTGGATCAACCAATTGTTTGCAGC
AAAACTGTGCAAATATGGACTAGCATTTATATCTACTACATCAAAATTCTTAGCAAAGAATCGATCTTCTTG
TTTAAAAGCTCTTATTGTCATGGCTCCTGCCACGGATTCCGCAAGATGGTTTGCCACCAAAGATTTGGTTGTC
CCATTGATCCGCATCAGCATTTTTGCAGACGAGAAGTAATACCTCTGTAAGCGAAGTGCCACATAAACTAGT
GGTACCGAGACAAACAAGACTTGCCAGGTGACAAAGATTAGCACCCCCATATTTGAAAAGAAATTTGTTGT
GGCAGCAAGAGCAAATATCAGGTTAAATGGAATATCAAGATCAATAATACTCAAATCAACCGACACCCTGC
TTAATACCCTACCCAAAGGGGTTGAATCGTAAAACGACATTGGTGCACGAAAAAGAGAGATTAATAGCTGT
GAAAATATCGACTTTGAAGATTGTAGCCCCAATGCAACTGTGAAGAGTGATCTGAGGAGCAAGAACCCAAT
TGCTATAACACCGATAACTAAGTAAACCACAATCAGTTTCAAAGTGCTGACATCGGGATTGTCCACATTTGC
AGCCATCCATGAATTTTGACATACATTACATGCCACGAAGGTCACTTGTGAGAGTACAGCTATGGAGAAGA
ACACGTAACCTTTATTCTGACTTAAATATTGTATGTAGGGTCTCAAACCCGTGTCACCTTCTTCCCTTTCTTCT
TTTTTGATCAATTGGTTGTTTTCCAGGTTTTCAGATAAATTGTTTGTGTGGCTGTTTTTAATGTCTTT
AATTGAAGGTATTTTGTTTGTGGAAGGGGTAACTTCTGCAAGCCTTTCGGAACCTGCGGTTTCTTTGTGTGCA
TTTACCAAGTCTTGAAACTCTTGGCTTGATGACATTAGTTGATTGTAAGGCGCAGCTGCTAGAATTTCCCCGT
CCGACATTAACAGAACAGAATCAAATGTCGGAAGGAAATCAACTTGGTGAGTTACAAGCAAAACTGTCTTC
CTGGAAAGCGCTTCCATAACAAATTCCTACCAATTAATAACCATGTTTATCAACTTTGCATTCAGAGACTAA
TAAATTATTAACTAAAGATACTCATTTCTGATCAAGTATTAGTTTACATTAAATAAGCTTGAAGCAGTATGT
GCATCCACAGCACTAAAGGGATCATCCAAAAGATAGATATCTGCATCCTGGTATAAAGCACGAGCGAGTTG
AATCCTTTGCTTCTGTCCACCACTGAGGTTAACACCTCTTTCTCCTATCTCAGTGAGATCACCATACGTTAGC
AACTCAAGATCTTTCACCAAAGAACACTTCTCAAGCGTTTCTTGGTATCTCTGACTATCCATTGCAGACCCG
AACAAAATATTATCCCGTATGCTACCTGTTTGAATCCAAGCTGACTGAGAAACATATGCAATGCTCCCATAT
ACTTGAAGAGATCCCTCGATGATCGGAACCTCTCCAAGAATTCCAGCTAAAAGTGTGGACTTGCCTGAACCC
ACCTCTCCACATATAGCAATTTTTTCACCCAATCGAACCTGTAAATGAATCTTTTGAAGTGTGGGCTTCACTA
GATTCCCTTCCCAAGAAAGAGTTGCTGAATCGATCAAAATGTTATATTGAAAACCCTCCTTATTCACCTTCT
GCCTAACATGAGCGCTCTCGAGCTCCGGTGCCTCTAGAAAATTCAAGATTCTTGAAAATGCTACTTTTGCTT
GAATAGTATTCCCAATAACATCAGGGATAGTTCTAATAGGATCCTGAACCAAACGTAAAGTAGCCACAAAT
GTGAAAACATTACTAGCATTTAACGGAATCCCAATAAAGTAACAAGCCCCAAACGTAGCAGTTGAGACCAG
AAGTGGAGACGACCAAAAAAGAAAGCTATTATAAGCTCTACGAAGCTGAACAGCCGATAACCATTTATGCT
CGACTGCCCTTAACTTTTCAATCACACCCTTAAAATGAATCTCCCATGCGTAAAGTTTCAAAACTTTCATATT
CACAAGAGCTTCTGAGATTGCTTTAAGGCGTTCATCCTGTGCAACCATTAGCTTCGACTGGAACTGGTGTTG
TAACTTAGCGAGTGGTGCATTGCAGATAACTGTAAAGATGATGACTGTTAAAGATGCAAATGTAGCCAAAC
CTACAGCCTGAAAAAGAATCGCAAGTGCAAAGATAAGCTGGAGAGTGGTTGTCCAGCTTTGATGGAGCCAA
TTAGGAAACTCCCCGATTCTATAAGCATCCACAGTTGCATAGTTCATGATCTCACCGGCCGAATGTGTAATC
TTGGCAGCATTAGACAAATTCAATTGTTTTTTATAAATTGCAGCTGTAAGCAATGATCTAACTCTTATACCA
ATAACTCTGCATCTAAAGTCCCATTGTCGTTGTGATAACGACTCTAATATCTTGATGAAAAAAAGTGCTATA
GCTAACACAAAACCTTCGCCATTAAAGCTTTCATAACCTTCAGCAACTCTTATGAAAGCTTTAAGAAATAGA
GGGCCGGCAGATACTGTGATAATTTTCATCAGTGCAAAGAATCCAGATATTAGAAGCTCTTTTGAATTGCAC

ATCACGATTGTCCTCAAGATCGATGGATGAGAATTCTTCTGGTTATTAAACTGCTCCAAGAAACGTAAATAA
CATGATTCTGCTCCATCTTCAATGCGCAACTTGGGCATATCTTTTTCTTC
AAGGGTTTTCTGACTACCCGTTTTCATTAAAGGATTCAACCACCAAAAGGACATTACATTGAGAAAACCTGC
TTTGGCAAGAGGGGTGAGATTGTCATTACAAGCTACTTTGATGCCATTAGTCTCACTATTTAAGGGGGCATA
AAGGTCTTTGTCATCAGTTTCTTCATAGTTATAGCCCTTATAAGTGCATGACAATAATAACCCTGCTCCTAGG
AAACATGCTACATCCAAACCTGTCTTGATTGACACCTTTCTGCTATCAATCAAAGATAGAAGAGATAAGATG
CCAATGATTCCAGCAAAAAGAAAGGCAACTATCGACAGAAGCCGAAAGGGTCTTTTTGGAAAACGACATCC
CCATAGGCTCACTGCTAGTGAGACTGCTAACCATGTCAATCCATGACACAAGAGCAGTAGCCATTGATGAA
AAGGAAAAGCGGTGTGATTTGCGTTTATGTTTGTCTTCAATGTCCAAATGCCCAAACACAAGTACAGAGTAC
CGAGTAAAGCATTGAAGATTGCAGAAACCAACTGTAAACTTGATTTCCTCTGAAAGCAGGATGATCTTCCA
GTAGATTTCGTTGGTGGTTTGTAAAACATGTTGAGTAATAACATTATGAGGAGCACTAAATCGAAACAAAT
GATGGAGACATGGTTCATACACGAAGATGGATTAGCGATGAAAACCAAATCTGGACCACAATGTTTGTCAC
CACTGTTCGAACACCCACAGAATGAACTCCACACATCATCCATAATGTCTTCTACAGTATATATTTAATCAC
TACCCTCTTTGCCTTTCCCAACAAGAATTAGATGTACGGAATCAAGAAAAACAGAGATGCTAGTAACTCTTC
AATCTTCTTTTTATTACTGATATATATGAAAATGTGGTATTTATGATTGGGTGATATAATAAGGGTTTACGGT
AATTTGATAGTGAAG

>ABC_19_TRINITY_DN42119_c0_g1 len=5,420
ATTCTATACTAGCAATCATGCTTAATCATCATCTTATTCAAGATCGTTAAACACAATAGCAATTCTCAGTTTC
ATTTATGGGCTGAAAGAGTAAATAAGTGCTTTCCTTGTGTAATCCTAATTACATTTCAAGTTTGAAATTCTTT
GTTGGAAAGTACTATTCACAACTTCTAGAAGAGGCAAAAATCCACGCTTGGCATAAATTATTTCAACCTATG
TAGGCTTAAACCTAATTAGCAAAAGTTCGACATAGAGCCACGCCAATCTATCTGCTACCTTGCTCCAAAGTA
TGAAAAAAGGGCTCGTCTAAAATTACAATGGCAGTTGAAATTACATTCCTAGTTGGTGTACACTTGTGAACC
AACATGGATCTTAACTTGATCAGTAAAAATCCTACATCTGAACATGGTCCCAATCAATTGTTTCATCTTTGA
AGTCGTATTCTGAATGCCGGAGCTTGTTACGACCCAACTTGCCCATCATCGAAAGACCCTCTACTACTTTAT
AGAGCGCAGACCACCATCTCTCCCTTGGAACCTGGGACTGTTCAAGCGTCTCCTCAATATCTTTATCATGTTC
CCCTTTCAAAACACTCTGAAGAGTCACAACAGCGTCCTTTGTTCTCTTAAGGATATTGTTATCATCTACAATT
TCCAAATTCACAAGGTCGTTTTGAGAGGAAGTGAGGCTAGCAGAAAGAGCAAACTGAGCGGCAGCCGCCC
ACCGTGAAGAGGCAAGCCATCTCCTCTGACCATCTATTGCAGCCTTCTCTGCCTTCTCTCCATCTGCTCCAAA
TGCTAAGTTTCGTAAATATTCTGCATTTGCAGCCCCAGTACTTTGAACCATTTTCGAGAAAGCAGTGTGTTCA
TCTTGTAAAAGTTTTACCGGTGCATCATACTCAACAACCTGACCAGCATCAAGCAGTAGAATGCGGTCACAA
TCAATTATGGTATTAAGACGATGAGCAATGATGATCATGGTACACGACTTGAATTCTTCACGGATGGTCTTT
TGGATAAGTGCATCTGTCCTGACATCAACAGCCGCAGTCGCTTCATCAAGAACAAGAATCTTTGATCTCCGA
AGCAGCGCACGTGAGAGACTTAACAATTGTCGTTGTCCAACACTGAAGTTCTCTCCAGCCTCTGAGACCTCG
GCATCTAAACCCAAGGGATTCCTCCTGATGACTTCCTTCAAATGGGCCCTCTCAAGAGATTCCCAAAGATCA
GGATCATTGTGCTCATTAAACGGATCAAGGTTAAATCTCACGGTCCCTGAGAAAAGAACAGGTGCTTGGGG
TATTATACCAAGAACCTTGCGAAGATCTGTCAACCCAAAATTTGCAATATCACAATCATCAACAATAATTCT
ACCTCTTTCCAATTCCACAATCCGAAACAAAGCATTAAGCATGCTGGATTTTCCTGCCCCAGTTCTTCCAACT
ATTCCAACCTTATCACTTGGAGGAATGAAAAAGTTCAAACCATGCAGCACTGGAGGAAGTTCGGGCCTATA
CCTCAAAACAACATCTTCAAACTTTATTGATCCCGAAGAAGGCCAGCCAGGTGGCGGGCGGTTATCTTCAAT
AACAGGAGGAGCCTCAGAAGGTAACTCAATATAAGTGCCAACACGTTCAACAGCATTCAAGCTATTTTCGG
CTAAACTTGCAAGTCTAAGGACAGCCGTTAATAAACTTGTAATATTTAATGCATAACTTAGAAGTAGACCCA
TGGTGGATGCAAATGCTTCTTGATTTCTTGCTTTGCCATTTTGCATAACAGCGAAAGTTGCGGTCAACCAAA
TCATGAGACCACCAACTGTTTCTAATCGGATTGCTAGCCAGCGGTTTGCACTCATGTTGACTAGCGTGAACC
TAATGTTATTGTCCATGGAACTCCCATTAATCTTGGCCATTCGATCATAGGCTTGATATGCACGAATGCTGG
ATAGACCATTCAGTGCTTCCCCAAATTGTGCATACACGGGAGATCTGGTTATGGAATCCAATCGCTTTACCT
CACGCGCAGTGCTCTGATAATACAAATAAGCTGCATAGAACACCAGCAAAAGTGGCAATATAGCCCAGAGA
GACATGGTGCTCAATAATCCAATTAGAACAAAGGTTGACAAGAGTTGTGAAACTTGGCCCAAAAACATGTT
GACAAATGGGGCAACATTCCGATCTATGTCACCTAGATCTTTGGCAAACCTATTAATAATGCGTCCTAGGGG
ATTCGTGTGGAAAAAGACCATTGGAGCCCTCAATATGGAGTTAAGCATAGCATTGTGCAACTTTCTAGCTGC
AAAAAGGCTTGTGATGATCAACCAATAAGAATTTCCCAGAGTCACCAAAACTTGACCAAGTGATAAAAGTG
CATATATAATATTATAGAATAGCGGGCTGTGTATCTTTGGAGTGCTCTCATCTGTCCAAATACTTAACCATGT
ACTACTCATAATTCTTAAAGTTTCTGTTGCTGTGTAACATCCAAATAGTATACCCACAACCCACCAGCCTCCT
AATGCATCTTTGTACCTTTTCAGAACATTGAAGCTGACAACCCCTGTTTCCCGTTCTTCTTGCTTAATGAGAA
CAGATTTGGTTTCTTTCTTTTTATACGCCTCATTAGCTATCTCACCATTGGTAACGGGTTTTGACATTTGTGCA
CCAGTCTCTTTCCCCTCTTCTTGTTTCTGTTCCTCAACGTATTCTTCCATTTTCCCTGCATTTTCCATCAATCTC
TGGAAGAGTTTACCATTCTGAGAAAGTTCCTCGTAGGATCCCTCCTCTTTCACCATGCCTTCATGGACTAAG
AGTATCCTATCCACTTGTGATAGAAAATGAAGCTGGTTTGTAACTAGAACTCGTGTTTTTCCTTTTAATTCTT
CTTTAATACATTTCTCAAAAACCTGTCTGCCCACATGAGCATCTAGAGCACTCAAAGGATCATCAAACACAT
ATACATCTGAGTTAGAGTATACAGCTCTAGCCATGGAAACTCTTTGCTTTTGCCCTCCACTAATGGTCACCCC
TCTTTCACCAATCTCAGTAAGATCACCTCCTGGAAGCAAGTCCAAGTCATGGTGCAATGCAGTTACATCTAT
TGTCTTCTCATACTTTGCACGTTCAAAGGGGGATCCAAACAATATGTTGTCACGTACCGTTGCATTAAAAAT
CCAAGAGACTTGTGGAACATATGCAACTGTTCCTCTCATACTAACATGTGCCTCACTAACTGGAGGAAGTTC
TCCAAGCATAGCGGATACAAGCGATGTCTTCCCCTCTCCTGTACTTCCAACTATTGCCACCAAGCTACCAAT
TGGTATATCTATATTAATATTCGAAAGTGTCGGTTTATCAGCCTTTGAATCCCAAGAAAAGGATCCATTCCT
AATTGAGATAGCCGGGAGTCCTGGTTCAAGAGGTTGGTTTGGAAGAAGGAGTCTTTCCTCGGACAACAGCA
AGTCTTCCAAACGTTTCAAGGACACATTGGCATTTACAACCTGAGTAATCATATTTGGAAGCATGAATAGTG
GAAAACGCAGAACAGCAAACAAAGAGAGTGACGTAAATGCTCTAGCAGGTGTCAACTGTCCTCCAAGTAA
CGTGAACAACCCAAATGAAACCACAATCACCACAACCGGGATACTGTTTAATATAAAAGTATTCAACGCTC

CTAGCATTTGTACTTTCTTAAACCATGACAGCTCTTCTGCCCGAACACCTTGGACCTTTCCTTGGAAACTGTT
CTCCCAAGCATAGCATTTGACAGTGTCCATAGCTGCTAAGATTTCATTCATGAGACCAATTCTCTTGTCAGTC
CGCTGCAATCCTTCCTTTTGCATTTTTTGCATCTTACTGATAACCACAGTCTGTATGGGGAACATGAAAACTA
ACAATGCAGCACCAAGAAGTGAGGCGATGCCCAATTGTTGGTAGAGAAGAACCAATGCAATAATGATACG
GAAAGGGGCGGACCACAAAGTGTGAAGTGACTGGCTTACTTGCTGGAGAGATTCAGAATCGGTTGTCATTA
AATTTGTTATCTTCCCGGATGCAATGTTTCTGCGACTCTCGTTAGTTAGCCTTAAAGTCTTCCGGAAAACTGC
AGCAATCAGAGTTGAACGAAGGCGGTATCCAACCCGCATGACATTCTGAAAATATTGAGCCTCACATAGCA
CACCAAGTACCACTCCAACAAAAATTGAAAATGCATAGATGTAACCAATCTGAGCTGGACCGCGTTCTTGC
ATAGACAATAATAGCTGGTTCAGAATCAGTGGGCCAACAAACTGAGAAAGATCATTGCCGATCTTCCAAAA
GCCTCCCCACCAAAATCTTCCTCCAAGACTACGATGTAAAGCCCTTAAAAGCCATGGTTTAGGCTTGCGGAT
CTCTTCTGCCCAATATATTTGGAATTTGCTGTTTAGTGTTTCTGT
CTTGTCCCAATCATCCAGTTTCCATATATCCTTCTCTGTAAGAGGCCTTTTGTATCCTAAATGCATGAGTGGA
TCCATCCAAGAGAAGAAGATGCTAGAAATAATGTTGGTGTGTCTCTCAGGACAAATTTGTTCTCCGCCGGCA
AGTTCTTCATATTCATCATCATCCAAAGACTCTGCCCGTATCGGGGTATAGCCCGGATAAGGATCCAAAGTA
GGAAGGTAGAAAATCAAACATAGCCCAAAGAGAACCTGCAAGGCAACCTCACTGAAAAAGAGATAGCGTA
CATTTCTTGTATAAAAATTGCTCACGGAAAGCACAAGATTGAACAAAACAGCATCTCCAAGCAAGGCATAA
ACAACACCAAATCTCACCAACCAACGAACTTCACAAACATAAACAATCGATTCGAGACCGATCATGACCAA
CATACAGCACCATGTAAGAGCCTTAATAATTAAGGTAACAATCTCATACGGAGCAAGACTAAACTCTCCAT
CAACATTCAAAGCCGAAATTCCCATTATCAATCTAAATAAAGGCTCAGCAGTACAGCAAAAAGCCAATAAC
CCCAACCAATAATTGTAGATATTCGACCTCAATTTAAACCTTTGCACTTTAAAATCCTTCTTTGTCACCCATA
TCCGGTATATGCAAGC
TCCCAAAAGGACCAAGTGGGAAATTCCAGTAACCAAAGAATCTGTTGCACATGGTGTGTAAGGCCCAAATT
CATTTTCCACGGCCGTTGCCCAAACCCCATTTTCCACCGGGCGGCAATATAAATCTAGAGCCTTAAAACCCA
TCTTCCCCTAGTAGAATGAAGCGAGTGCCTCTACAACTTCAACTTCATTTTCCACCGGGCGGCAATACAAAT
CTAGAGCCTTAAAACCCATCTTCCCCTAGTAGAATGAAGCGAGTGCCTCTACAACTTCAACTTCATTTTCCC
CTTTCAAAATTGAACTGATGAGAATGATATGTG

>CYP450_1_ TRINITY_DN41388_c1_g1 len=1,867
CCGTGCGTATCATTTCCCCTTATTCATATATATATATATATATAAATTATAGTGCCGAATTTTTTTACATAA
CAATAAACATACATGGTATGCTTGTTATCTTTTCATATGCCTAGTATAAGATCTTTTACACACTTATAAGTTA
TAAACGGTGTTTTTATATTATATATAGAGGATATCGTCTAGCAGAGGCTCATGATAAAAAGGGTGCTGCAGC
AGGAGTTGCGCAGTACATTAAGGCCTAAACTGACACTCCATACACATGTGGGGATAATCTTGGAGAAACAA
ACAGCTCAAGTGAAGAAGCTTTGACATTTGAAATTCCAGGGCTTTCAGTCAGGTCAATCTGATCATTTGATG
GATTTTGGAACTCGAATCCATGTATTATATTGGCCAATATCAACTGCATTGCTTCAAGAGAAAAAGTGATTC
CGGGGCATTTTCTTCTACCACTGCTAAAGGGAATCAACTCGAAATGTTTACCCTTGACATCGATTTCCTTGTT
GCTTGTCAAGAATCTTTCCGGTTGGAATTCAAAGGGATCAGTCCATACTTCAGGATCATGTTGAATTTTCCA
TAGATTTACCAAAAGGCGAGTCCCTTTAGGGATAGTGTAGCCACCGACAATGCAGTCGTCGGTGGAAACAT
GAGGAAGAGAAAGTGGTATTGCTGGATAAAGTCGCATCGTTTCTTTTATAATTGCTTGAAGATAGACCAGGT
TCTTCAAATCCGACTCCTCAACAATTCTATCATTTCCCACATATTTTTCGAGCTCTTTTTGGGATTTTTTCAGC
ACAAGAGGATTGTTGACTAATAAAGCTAAAGCCCATGTTAGAGTCGCGCTTGTCGTGTCAGTGGATGCTATT
AACAAGGCCATGCATGTCGCTTTCACGACCACATCAGCGCTGAATCCACGAAGATCCTCTTTTGGTTCTTTA
AGACGGGATAACAACATGGCCATGAAGTCCTGGTCTGTGCTCTCATTTACCTGCTGCTTATGCTCTTCCAAC
CACCCACTGATCAGAGCATCAATCTCTATTGCTGTCTTCTTCATCATCTTCTCGTATCCCCCTAAGTCTAACC
ACCTCAAGCCTGGAATAGCATCAGATGGCACAAAGGCACCAAATAGTACAAAAAACTGCCTAATGGCCTTC
TTGAATTGATCTTCATTTTGTTGACCATGGGAAAAACGGACGCCAAATATCACCCTCACAGCATAATTCAGC
ATTAAGTTTCCAAACCATTCTTTCATATCAACTTTTGTCATTTCAGAAGACCCTTCATTTCTCTTACAAATTCT
ATGCATTTCTAATATGGACGAGTTCACTTCAGATATTCGGATATGTGCTAACTTTTTAAGGTACTGTTGGGAC
AACAGTTCAAGCACAATTATCTTGCGCATCTCACGCCAATATGGACCATATGGCGCAAGGCCAACAGTAGC
ATAGTTGTAGGCCATTAGCTCGCTTGCCATGGCTTTTGGTCGACTAGCAAAAACTCTGTCGTTCGTAGTTAA
GCACTGTTTGGCCATTGCTGAATTGCTAACTATTAAAACAGGATGCACACCAAGCTTTATAGTGAAAATGGG
TCCAAACTTGTCAGCCATAGACCCAAACAGTTTGTGATGCACTTGAGAACCAGCTAAAAGATGCAAGTGAC
CAATTATCGGCCATGACCCCGCAGCTTCCAGTACTTTCTTTCCCTTTCCGTTATTGCTGCTATTGGCTCGAGC
TTTTATCCATGCCAAATGACCAAAGACCAGTAAACAAAATATGGTGGCTACCGAGAATATGCAAGATAGAG
GTAAATCCATAACAATATTTTCTGTGCATTAAAGGGTTCATATTTGAAGAAGGTGAGAAAATG

>CYP450_2_ TRINITY_DN27559_c0_g1 len=555
GGGTGCCTCACTAGCTCACTTAGTGCCCATTCTATAGTTGTGGATGTTGTATCGGTTCCACCAGTAAATACAT
CGAAGATGACTGCTTTTATGGCATCTCTCTCAAATGTAAATTTCGTTGCATTTTCTCTTTGAACATCGAGTAA
GATATCAACCAAATCCTGGGCTTCTTCCTTTTTAACATCAACATTAGCATCCGTCTCTATGTTCCTATTTATA
TGCTCTTCAATTACACCATCAAGAAACTCGTCAAATTCTCTAGCAACTTCATTTGCCCGTCCCTCTAAACCAC
GCAACGAATCCACCCATGATAACCAAGGAATATAACTCCCTACACTAACAACACCAAGTAGGATCTGAGAT
CTTACCAGCATGTCCTTAAACTTTAAATCATTATATGCCTTACCTAAGGCCACCTCACATATTATGTTGTTAG
CAAGGAAAAATAACATCTCAGTCAAATCAACTATAGAACCACAACTTTTCCCAATCATTTTAATCATATGAA
TCGTTTCCTTTTCTCTTTGTTTTCGAAATGATTGAACTCGTTTATGA

>CYP450_3_ TRINITY_DN22805_c0_g1 len=474
GGCAAATGATCTCCTAAATTCGACGATTTCCCTAACAAAAACGTCTCCTTAACGATCTCTTGAAACCGTTTG
CCCAACTCTATATCAACCCCAGTAAACCTTTTCCCGGATATCATCATCATCATAATATTCGTTGTATAGTCGT
TGAATACCGACTTTAAGTCCACGTGCGAAGAAGAACATTCGGACGCAATACCTTGTACCATAAGTCTTCCTT

CATCCGCGCGTATGCTATAAAACTCATTTATCCTGTGAGAGGATAGTATCTCGGTACTAGAAAGTTTTCGTA
AATTGAGCCAGTGTTCGCCATAAGGAGACCACGCCATGTTGGTGTAATTATCTCCGAGAATTTTGCCGAAGA
TTAAACGTGGGCGGTTGGCGAAAATGATGTCGTTTTTTGTGAAACATTCTTCGGTCGCTATAGGGTCGGCGA
TTAAGAGGACAGGGCGTGACCCGAAACGAAGGAGAAGGATC

>CYP450_4_ TRINITY_DN34846_c0_g1 len=365
GATTGCTGTTCTTTGGGAAATGATCTTTAGAGGAACTGATACTGTGGCGATATTGTTGGAGTGGATTTTGGC
AAGAATGGTGTTACACCCAGAGATACAATCAAAAGCACAAGAAGAGATTGAAAAAGTTGTCGGGTCGGCC
CGGCCCGTTTGTGATTCGGATCTTCCAAACTTGCCTTACCTCCATGCAATTGTGAAAGAAATTCTCCGTGTGC
ATCCACCGGGCCCACTACTATCGTGGGCTAGACTTGCTATCCACGATACTCAAGTGGGTCCGCATGTTATCC
CAGCTGGAACAACCGTTATGGTCAACATGTGGGCCATAACTCATGATGAGGGTATTTGGGTTGAACCTGAA
AGGTTTA

>CYP450_5_ TRINITY_DN15853_c0_g1 len=2,052
TTTTTTTTGACCTTTAAAAATTGCAAATTTGTTCCGTTGTATATTAATCCATATATTAATTTTGAATCATTTAC
AAATACACATGAGGTGTGAGACGAAGTTCATTCCTTTTGTGCATGGTTAACTCTGGCATAGCTTCAAAGTCT
ATATCATCTTATGCAGACCATCCGGTAAGCCCCCTTATACATGTAAAGCAGAGTAGCAAGCAAAAGCTCTG
CTGTGACTCCGGTTAATATTCCAAGACAGATTCTTTGGCCAACACCAAGAGGAATAAACTCAAAATTGTTCC
CACCTTTATCCAAATTATCTAGTTAACTAGTATAAATGAAAGACATGACCAAATAAAAATTTTGTTCATACA
ATTAATTTAGATAACGGATACTATTCTAATGAGAGTTTCGTTTTTTTAACAACTAAGTGCGGGATTATGGGA
TTAAACTAGTAATTTTTGCATGCTGGATCATAGTTTGATATATGATGATATATATGAAGATGCATATATATG
GTTGATCAGTTATTCAACGATCGAGCTTTAGATAATATAGTAGTCCTTTACGTGTAAGCTTGGGCTAAAAGA
CAAAGGTCGTTCTTGTTGTTTAGAGTGAATCCAGGCATAACACTAGAGTCTATGCCTTCCTTGTTTATACCAT
CTGGCAACACCCAATCCCACATATAAACAAGATTAGCAAGCAAAAGCTCCACCTCCGTAACTCCCATTGTG
ATTCCAGGACAGATTCTTCGACCACCCCCAAATGGAATCAGCACAAAATCACCTCCTTTATAGCCGATATCA
TTACTCAGAAACCTTTCTGGTAAGAACTCCTCTGGATTCTCCCAAGATGCAGGGTCTCTTCCAACGGCTAAT
GCGTTCACATAAACTAGAGTCTTCTTCTTAACTTTGTAGCCTTGTATGATGGTGTCTTTAATTGTTTCTCGAG
CTAAGAGAAGTGGAGCTGCAGGGAATAATCTCATGCCCTCTTTCACTACTGCCTTCAGATAGGCGAGTTTTG
GGAGATCATCTTCATCTACTACCCCTTTCTTTCCAACCACGTTCCTCACTTCTTCTTGTGCCTTTTTCATCACT
CTAGGATTCTTCACTAGGCCTGTCATCGCCCAAACCATTGTAGCTGCACTATTATCTGTCCCGGCTACTAATA
TATCCGTGAGCATGGCTTTTATGTGATCATTAGTGAGTTCAAAGGGATGCAAAACTTGATCTTTCTTGAGCT
GAATTAAAATGTCAATAAGATCTTGGTCTTCCTCTTTAGATTTTTGGTTCCGAGGGTCGAGGTGTTCATCAAT
TAGTTCATTGTAAAATGAATCCAATTCTCGGAAGATTTTATCCAAACGACGAAATTTCCCCATCAACCTGTC
AACCAAACGTACAAATGGCAAACCAGGCCAAATATCCGAAAAAATGTAGTTTGTTACTGTCGCCTGAACTT
CATCAAGCATCCGAAGAATTTCCTTGCTCTCATTTCCATCTTCATATCTTTTACCAAAACCCATTCTCGTGAC
GATAGTACTCATCACGGTGTGAGTTATTTCACTTATGTTTACATGCTTAGAAGAACTTGCTAGCCTACGTATT
TTGTTCATGGCACGCGACACCTCATCTTCACGAATATGCCTAGAAGATAGTAACCTTTTTGGGCTGAACAAG
TAAACAGTAAAAATCTTTCTCATTTCTTTCCAATTCTTGTTGTATGGTGAGGCAGCTACATCAAGGCCATTGT
AAGTTACTTTTTGTAAGCCATGCAATAATGGCCTACTACAAAAAGATTGATCTTGGGTTTTAAAGATTTCTTT
CGCGAGATTTGCCGATGAAACAACCATGGCTGGGACAAAACCAAGTCTTAGGAATACAATAGGACCATAAG
ATTTGGAGAGGTTCCACAGGGAAGTATGGAGGCTTGAGTTATCGATTTGGTGCATGTTTCCAATGAAGGGTA
GTCCATGAGGGCCTGGTAGGCGACTGGATATTGATTTGCTCTTGGTAAACAATGTAAGATAGAGTAGAGAT
AGGATGGGAAGAGATACAACAAGAAGAAA

>CYP450_6_ TRINITY_DN35527_c1_g1 len=1,646
AACCATATATACAATTAAGTGATGATCTATACATTACTTAATCATCTTTATTAAATTGAACGCCAAATTATTC
AAGGCCATAATTACCTAGCTAGCTACATGACTTTCGTTAATTTGTTGCTTCTTTGAGTTACAAATTAAACAAA
GTACGTAACTTAATTAGTTATTATAATTGAAGGAGTTTTTGAATACAATGAACATACGCTCGTAAACTGTCT
ATCGTAAAACCACATGACATTTCATTGGCGTCTTTAGCTCAAGAGAGAGTTTTAGGCACTCGGAAAGGTCTA
CTACTTTCGCGGATGATGGAAGCCATTTATACTGTTGGAGAAGTCGTGCAAGCCACAAGTGAACCGTGGCT
AGTCCTAACGACTTCCCGGGGCATACCCTACGACCAGAACCAAAAGGTGCGAGCCTAAGGTCAGAACCCAT
GATTGGGAAATCTTGTTCCATGAACCTTTCTGGCTTGAAATCCCATGGATTTTCCCATATAGACGAGTCATGT
GTGATGGCCCACATGTTAACCATTGCGGTTGTCCCTGCTGGGATGAAAAATTTACCAATGTGTACATCAACG
GTTGCTAGACGAGCCCAAGAAAGTAATGGGCCTGGGGGGTGCAACCTTAGGACTTCTTTGACAATGGCTTG
AAGATAACCTAGATTTGGGATATCAGAATCTTGAACATGTCTGTGTCTGCCTACCTTCTCATCAATCTCATGC
TGAGCCTTTGCTTGGATGTCCTGGTGCAAAACCATTCGTGCCATGATCCATTCGAGGAGGATGGCAACTGTA
TCTGTTCCTCGAAAGATCATCTCCCATAGAACAGCCAACATATCATCATGTGTGAGTTGATCCTCATCAGGC
AAAGACAACAATATACTAAGAAAATCATTTTGTCCATTGTCAACACCTTCAAGTTTTCTTTTTCTTCTTTCTT
CAATAATTTGACCTACTAGATTTTTTACTTTTAAAGTCAACTTGTGAGACCTCCTTCGGACCCCACTAAAGTC
CAAAACCCTAATTGGAAAATAATCACCCCAATGAAATTCACCTATCAACTCATACCCTTCTTTCACCATAAA
CCTTAATTCTTCTTCTTTTTCAATTCCTAAACCACATCCAAACACACTCTCCATGACATTTCTCAAAGAACCT
TTTTGCAATATCCCTCTCAACTCCACAATCCTTCTCTCCATCATCTCTTTTGACACCTTTTCGACCATTTCATT
GCACACTTGTTGTCTAACCCATTCGAGACCCGAAACTCTTTTGGGAGAGAACATGTGGTTTGAGGCAATCCT
CCTTAGATGTCTCCAATACATCCCCGATGGTGCAAACCCGATAGCTCGTTCAAACATAAGCAAACTTGCGGA
TTCCTTTATAGGACGGTCCGAAAAGGCACTGCCACTCAAAATTTCCTTTGCAGTCTCAGGGTGACCAGTAAT
AACCACACGAGTTGAGCCAAGGCTAAATGCCATAAGACGAGTTGCATCCATCGAGCTAGCCATTGAGGCAA
GTTTCCGATGAGCATGATAACCCATTTTAGGTAAAATCCCTAGGAAAGGCCAACCGACCGGACCAGGTATT
TGACTAGGGTTTAGCTTAGAATCTCTCCATGCAAACCCGCCAGGGACTAACCATGTGT

>CYP450_7_ TRINITY_DN35866_c0_g1 len=2,341
GGAATCATTTATTCATTTTCATACTCGATGCTCGTTCTGCATTTCCAGCATATGCCTAGTTACACTTAAGAAA
AGTAAGAAGCCTAATACAAAAATCATGATCTTGTAGCTTGTTACACTTCTCAACTGTTCTGCCATATCTATGT
TTCCACACCAGAGAAGGAAGTTGCAACCAGTAAAAACAGTTCAATCGACAAACAGATTCTATATTATACAA
TACCGTTTCAGTTTTTATTTATTTATCTACAACACATTTCCAAATGAAAGCAACATACACAATAAGTATTAA
AGGGTTACCTTTAGAGCTAGACTAATCAAACAACACATATCAATCTAAAAACATATAGTGTATTATTTATCC
CATGTATAGGTAGAGGCTTTCATCAGATAATCTTTGAGACGGTATAGCTATTAGGGGTTTCCTTTTCTTCATC
ACAAATCCAAACTTATCGGAAAGTTCAAACACTTCATCTTTGGGCAACCTCCACTCGAAAGAATGCAACAG
CGATGCTAATATGTATATTAACATCTTTTCACCCAAGGGTATCCCAGGGCAAATTCTTCTTCCTGATCCAAAT
GGTAAAAACTTGAAATCGTTTCCACTGTAATCCCATTTATCGTCAAAAAACCTCTCAGGCTTGAACTCCAAT
GGATTAGTCCAGTTTTGGGGATCTCTGTGGATAGCCCAAGTGTTCATGTAGACAATGGTATCCTTTGGGATG
GTGTAGCCGCCCACCTTGACCGATTTATTTGGGCATCTCTGGATTAGGAGGGGAAGTGGAGGGTGTAGTCTA
AAAGTCTCCTTGATCACTGCATCCAAATATGTTAATTTGGGGAGTCTAGACTCATCCACAATACTGTTCATG
CCGATAATCGTTGTTAACTCTTCCTGAATCTTTGTTTTCACACTAGGATTATGCAAAATCTCTGCCATCACCC
ATTCGACCATTGCAGATGTCGTGTCTGTTGCTGCAACCAGTATGTCCATTAGTAAGGCCTTTATTTGGTCAAT
GTTTATTGATGTTACAGCAGCATTCTGTTCTCTAAGATCTAATAAGATCTGGAGAAAATCTTTTCTTTCAGCT
CCATCAGTTTTCCCAGAGTTGGCATTGATTCTTTCTTCGATAATATTGTCCAAGATCATGTCCACATTTTGCT
TTTGCTTCAGCATTTCTCTCTGTTTTCCCTGTAGATCGAACCATGAAAGTATTGGAATAAAATCAGAAATATT
TGGTTCTCCAATTAATTCGATTATCTTGAAAACCACTTCTCTAAACCCGTCTCCAATATAGCTATAATCCTCC
GGTTCACCAGATTTGCTGCAACCCCATAACATGCTAGTCACAACGTTAACCTCAGTCTTGAAAGCAATATGG
TTTATATTGATCTTATTCCCAATATTACCATAAACTTTATTCACAATCTTTCTCACTTCACGTGTTCTAAAATT
CTGACTCGCTTTCATATTTGCATTGCTCAACACTTGGCTGACCAATAGCTTACGCATATTACGCCAGTGTGAG
TTGTTGGACCATGCCACATCTTGACCTCCGTAAGTGATAGAAAATGCTGTAAGTGGAGGAATACGGTTAGC
AAAGGTTTGGTCCAAGTCACGAGCCACAACCTTTACTAGGTCCACAGAGTTCACTACAACATGAAACTTACT
TCCAAGTTGCAAGCTGAAGATGGGGCCGTATTTGTGAGCTATCATGGTGAATACTTCATGTAAGTTGGAACC
GAGGAATGGAAGATACCCTACAACTGGTAAGCCTTTGGGGCCGGGTGGCAAGAGAGCCGTTGTGCCCTCTT
TGGAATATGAGAACATCCATTTGTACCATAGTAGTACTAGTGTTGCAACA
AATATGGTGAGAATCGTACGAGCAAGCTTATCTTGTTCATTATTGACTTCCCACCACCATGACCACAAGGTG
ATCATTGTGATATAACTCTTATATCCAAGCTCTAAAATCATATTATTTGCTGCAAACGGAATGTGATCTCAG
TTCATTCTATGGACTAGGAGACATTTCACCTGCATATAGCATCTCTATGAAGCTCAAAGATGATGTAGTTCT
TGAGTGGGTTCTTTCTTAGTTGCTTTGATCGATCTCTTCCTTTACAAATCAAGAAAACGAGTGGCCTTAATTA
CATAGAATTTTATAGACAAATAAATATTTGCTTATTCCCCTCATGTATTTGATTTTGGCGTCCGCGCCAAAGG
AAACTCAATCATATGACTCAAGTGGCAAAGTACTATTTTTCTTTTATGAATGTGTCCAAGAAGTGAGATTGC
ACGCTGATAAGGGCGTGTCTGTTTTTATTTATTAAGTGTTATCCAG

>CYP450_8_ TRINITY_DN25141_c0_g1 len=1,495
GAGAGGGTTAGCTCTAAAAAGAGTGAGGTTTAGTCCCTCTTCCATATCAACACTTTGTAAATCTCCGTCTTT
CCCATCCTTCCATTTGAAACATTGAATCATAGCGCCGAGCATTGCTGGTACCATCTGCATCACTAGTGATGT
TGGAGGGCACATCCTCCTCTCACTACCAAATGGTAACATCTGATAGTATTGTCCTCTCACATCCACCGGTGT
CTCTAAGAATCTTTCCGGCTGGAATTCAAGCGGGTTATCCCAATAACTTGGGTCTCTCCCAATGGCCCATAT
GTTCACAAAAACAGTAGTCTTAGCAGGAATCTGGTAACGTGTCATGGTACAATCTTTTGTTGATTGTCTCGG
AATCAACGACGCAGGTGGGTAAAGACGTAATGTTTCCTTGATGATTGCTTGCAGGTATGGAAGGGTTGGTA
TGTCTGATTCTTGTAAAAGTCTTTTTTTTCCAATAACTTGATCGATTTCATTAATTGCTTTTTTCATGATATTC
GGATGATTTATGAGTTCTGCTAAAGCCCATTCAATTGTAACCGCTGTAGGATCTGTTCCTGCAGTGAACATA
TTTAAGATGAGGGCTTTGATATTTTCTCTGCCCAACCCAATCTCCATGCTTCCATCTTCTTGAATATCGAGTA
AAATGTGGAGCAAGTCCTTCCCATGTGATCTAGTCTCTTTCTGTCTTGCCTCTTCATGCTCTTTAATTATTCTT
TCTATCAATTCATCATGTCTGTCTCGAATATCTTTCAACTTTGTCCCGAATCCTTGCAAGTCTAGATTCTTAC
AAAATGATAGATAGTCCGAGATGTTAAACTTACCTGCTATCTTAGTTATTTCTGCAACCAAATTCTTGACATT
CTTTGTCTCACTATCTTTGTCGGAACACCGTTCATTCATAAGCATTCTTGAGATCATGTTGTTAGATGTCTTC
ATAAGTTCCCCGCTTACATCAAACGTTTTCCCCTCTCTAGCCATATTAGCTAAGGATACCATAAAAAAATTA
ATCTCATCTTGCCTAACAAAAAGGAGCAAGTCAAGTGTTCCACGATTTAGGAGCCGTGACATAACTATCTTA
TTCATAAACTTCCAATACGACCCATAGGGAATAAACGCAAAGTCTTTCTTTCCATACGTTAGGTAATCTAAG
GCTGCGTTTTGTGGTCGATCTAAATTGAAACTTTCATAGGTTTTAAGGAATTCTTTAGCAGTTTCGGGTGAAG
AAACTACAACACAAGGTACATTGCCCATAAGTACCCGAATAACTGGCCCATATTGAAGAGAAAGCTTGTGA
AAAACTTGGTGAGGGGTAGGAACAAGGAGGTGGAGATGTCCAATGATGGGAAGTGCAAAGGGGCTTGGCG
GAAGACGAAATTTATTAGTACGAGTCGATTTGAACAACACCATGATCAAGATAGTTGAGATCAGGCATATG
GAAAACATGATAAAGTAGCTTTGGAAAACCATCGTTAGGCGAACTTTAAAA

>CYP450_9_ TRINITY_DN31873_c0_g1 len=1,833
ATATTGTTTTGCTAAATTTTAGATAGAAATGGCTGTGTACGTGTGAACTGTTAGTAGGCCGGGTGCTTGTAT
ATATAAAAGCAAAACGAATGGAGCTCCTTCAATATAATTATTTACACTCCATGGAGATAACATCTTTTCAAA
ATGTTGTTCTAATCTTCACCATATTTCTAACAATCTACTATCTCTTCTTTCCTAAATCCGCCACTAACCAATTC
GGGTTTAAAAATTACCCAATACTCGGAACTTTACCCGAATTCCTCCTAAACCGTCACCGTTTTCTTGAATGG
AGCACCGATGTCCTCTCTTTGTGTCCAACCAACACCGCGACCTTCAAACGCCCCGGAAAAATCCATGGTGTT
ATTACTGCCCACCCTGCAAATATCGAACATATGTTAAAAACCAATTTTGATAACTACCCTAAAGGAACCCGG
TTTATTTCCCTCCTTGAAGACTTCCTAGGCCGAGGTATATTTAACTCGGATGGTGAAGGATGGAAGACGCAG
CGCAAAACTGCTAGTTATGAATTTAACACACGTTCCCTTAGGAACTTTGTGTTGGAAACAGCTGCTTTGCAG

CTATTTACACGGTTCATACCTGCTCTCGAAAGGGCAGAGAATATGAACCGTGTTGTTGATTTACAAGAGATT
TTAGAGAAATATGCTTTTGATAATATATGTAAAGTAGCTTTTAATGTCGACCCTGGTTGTCTATCTGGAGAT
AACAAAACTACCTCTAGTACTAGTAGCGAATTTATGTATGCTTTTGAAGATGCTGCAACTCTTAGCTCTGGA
AGATTTATGTATGTTTTTCCAGGAATTTATAAAATTAAGAAATTTCTAGATTTCGGTTCAGAGAACAAGTTG
AAACAATCTATTGACACGGTTCATAAGTTTGCTGATGGTATCATAAAGTCACGAATAGAAGAAAAGGGTAA
TGAAGATACAGAAAAGGATTTGTTATCGCGATTCATGGGTAATTCGGACTTTTCACCAGAATTTCTTCGGGA
TATTGTGATAAGTTTCATATTAGCTGGCCGTGATACGACTTCATCGGCTCTTACATGGTTCTTTTGGCTTTTG
GCATCATATCCAAAAGTCGAGCAAAAAATACTAGACGAACTGAAAACAATTCGGTCTAGCTCTAGCAAAGG
TCTCAATGATTTTTACAGTTTTGACGAGCTTAGACAGATGCATTACTTGCAAGCCGCGATATCTGAGGGATT
ACGATTATACCCTCCAGTGCCAGTGGACACAAAGG
CTTGCTTAAAAGATGATATTTTGCCGGATGGACAGTTGTGAAGAAAGGTTGGTTTGTAACTTATAGTACAT
ACGCAATAGGAAGGATGGAGAGTGTTTGGGGCAAAGATTGTCGTGTTTTTAGACCCGAACGATGGTTGGAT
TACGATGAGAGCTCTGTTGGAAATGTGATTTATAGACCTGAAAGCCCGTTTAAGTTTCCGGTGTTTCATGGT
GGACCAAGAATTTGTTTGGGGAAAGAAATGGCCTATACTCAAATGAAGTTGGTTGCGGCAACTATCATCGA
GAGGTTTCATCTCGAGATGGAAGAGCCGGCCGACGAGAAGAACCCGCCCGGGCATGTGTTATCATTGACAA
TGCGGATGAAAGATGGGCTGAAGGTTAGAGTTAGGAAAAGATGAATGAGGTCATTTGGATGATATGTTTGG
AGTTAATTTATATTGCTTTATTAATAATGTACTATGACTCTATGAGTAAACATTATACTCGTACTTTACTAGC
ACCATGAATAAAAACTTTATTAAAATTTGGTTGAAAAGTAGATAATTATCTAATACTGTTTCATTTTAAAAA

>CYP450_10_ TRINITY_DN73328_c0_g1 len=399
TGGAAAAGATCAAACATATTCATATATATATGCACTATATATACTTTTACAGAAAGTAAATATATTAGAACT
GGATACACTGAGACAATAGTTATTATAAGAAAAGATATGATACACACACGAATAATCCAAAATACGAACTT
CCATTTATTTTTGTGATAAGCGTGGGGAAACTAGGACTTCAAGTGGAGTTGCCTTAGCATTGGTCATCCCAA
CGCTCGCGGTCATATCAACTGGTTCATCGTTTATGGTCGACATCTCAAAATTTTGTAGCAAAGTGGCTAAAG
TTATAGGTAACATTTGAAGCGAGAAACCTATCCCAGGACAACACCTTCTTCCAGCACCAAAAGGGATCAAC
TCAAAATCGGCCCCTTTAACATCCACATCCCTATGGTTTGG

>GST_1_ TRINITY_DN34237_c0_g1 len=1,759
AAGCAATCTATCTCTTTTTTTTTTTTTTTGGTTAGAAAGAAATATGGGGGAACGGAGTAATCAAACTCCACTTT
TAAAAGCTTCAGAAGATGGCAAATCTAGGAGCAAATGGACAACTGCAACTTTATTTTTCACTTCACTCACAG
CTCTTATTTTTATCTTTATTACAATAGGAAGCAACTCAATTCCATTTAGTATAAGTGATGGGAAGATACATA
GCAAGAGTGTACAATTAGGCAATGTGTTCAAGTTACACGAAGCAACGAATAAGATACCACCGAGTGATGTC
GTCGAGTCATGGGAAGCAGTCGTGGCAGCAGATGATTCCCGTTGTTCAAAGATTGGGGCATCGTTTCTTAAT
GCTGGTGGTCATGCTGTTGATGCTGCTGTCTCGGTTACTTTGTGTGGCGGAATTGTTAATCCGATGGCTAGTG
GTATCGGAGGCGGTGGGTTCATGGTGGTTCGCTCAGCTGACACATCAAAAACACAAGCATTCGATTTCAGG
GAAACGGCCCCTTTAGCTTCTTCGCAGAACATGTATCAAAACAATCTGGACGACAAGTATAAAGGAGCACT
AGCCATGGGTGTCCCCGGGGAAATAGCCGGTCTTTACAAAGCTTGGTTACAACACGGTCGCTTGCCATGGA
AATCTTTGTTTCAGCCTGCCATTCGCCTTGCCAAAGACGGGTTTGTAGTTGCTCCTTATCTTGCAAGTTCACT
CTCGAGTAATGCTGACAAGATACAAAAAGATCCAGGCCTACAAAAAGTGTATGCGCCTAACGGGGAGATTT
TAAAGGCAGGTGAAATGTGTTATAACTATGAGCTCGGTTTGAGCTTGGAAGCTATAGCAGAAAGAGGTCCA
GAGGCTTTATATAGTGGTGATATAGGGGAAAAGTTGGTTAGAGATGTTCGGTTGGCGGGTGGGATCTTAAC
AATGGATGATCTGAAAAACTATAAGGTGAAAGTTACTGATGCAGTTGAAGTGAACACGATGGGTTTCACTA
TTTTAGGGATGCCACCGCCGTCAAGTGGTACTCTTGGGCTAGCTCTGGTAAAACTGCATCAGAAATGCTTTC
TCCTACATTCGCGAAGAAAGTTAGAGAACGGATTTTTGACACCACCACCTTCCCTTCTGAATATTATATGCC
TGGGTACATTCAATCACCTTTGGGCTAATATTAGTCAGTTTTGACCTCAAGTATTAAACTGTGTATCTTACTG
TGTGTTGACTAGGTGGAGTCAGATTCAAGACCATGGTACAAGTCATTTTTGTATAGTAGATGCAAATAGAAA
TGCTGTGTCAATGACAACAACTGTGAACTACCCATTTGGTGCCGGTGTTCTATCTACAGATACCGGAATTAT
ACTAAACAACGAAATGGGTGACTTCTCATCACCTACAGAAATATCCCCTGACAGCCTTCCTCCTTCACCTAC
AAATTTTATCAGTCCAAATAAGAGGCCGTTGTCTTCCATGACTCCTATCATTATTGTCAAGGGTGATCAGTT
GGCTGGGGTTATTGGAGGAAGTGGTGGGATGTACATAATTCCAGCAGTTATACAGGTCTTCCTTAACCATTT
CATTCTAGGAATGCAGCCCTTAGATGCGGTTCAGTCTCCAAGAGTCTACCATAAGTTGATGCCGAATGTGGT
GTACTACGAAAACTGGACAGTGATAGACGGGGATCATATAGAGCTTTCAGACGACCTAAAACAATTCTTAG
GAGAGAGGGGTCATGTACTGGAGCCAAAATCTGGCG

>GT_1_TRINITY_DN32482_c0_g1 len=830
ATATTATTTTGCTAGCCTGCCAAAGCAACAACACAGTACAAGGACAGGGTAGTACAATATGAAAGTATAAC
TAACTTGAATAAAATATACAGTTTCTTTCTAATACGGAGCTAGTATTTTATTACACTATTATTGTTTATGATT
GATTGATTTATATGGATAAATATGCTGATCTCTATGTGTTAAGCCGTGCGCCGCCTTTCAGTTAAATCTTGGA
TCAAGGATTTCACATTGACATCAGAAGATCCCCCTTCAGCCATTGCTATTTTTGCCATTTCTGAAAGCTTTTT
CGCTCTCTTTTTTCTTTGTTTCCCCTCTTCGTCTTCTGCCATTAGGCATTTGACAGCCGTCTTGACGTCCTCCTT
CGTTACCAGTGCTTCAATCTTATCTTCTTCCCCGTAAGGAACAGGAACTAGGTCCACACCAATCCTAACTCC
AATCTTCAAAATCTCTACAATAAATGTTTCATTTAGAAATTGGTCTACGAAATGCGGCCATGTAACCATTGG
CAAGCCAGCACACACTGCTTCCAAGGTCGAGTTCCATCCACAGTGTGTCAAGAAACCCCCGATGGCTGGAT
GCGACAGTATCAGAACTTGCGGCGCCCAACCATGCACGATCAACCCTCGGTCTTTTACCCTTTCTTCAAACC
CTTTTTCTAAAATCCATTTATCCAGTTCTTCTGTTTCCTTTCTCACGCACCATATGAACGGTTGATTGGTGGAC
TCAAGTCCTAGTCCGAGCTCAATCCCTTGTTGTGTGGTGACGCGAGCTAGACTTCCTAAGCAAACATACAAC
ACGGAACCAGGTTCCCTTTCATCAAGCCATTT

>GT_2_ TRINITY_DN30716_c0_g1 len=1,635

GGAAGTTAGATATATACAATAGCAATATGATCAAAGTTTAAATACTTACTATACATACTTACTATACAATAG
CAAAATACTTGCTATACAATAGCAAAATAGATAATAGAGTTTGATACCCGCAATATATGTCGCAAGTTGGA
CATAACAATTGTTAAAAATTAAGAGTTTACTACTACTCCATGATAGAGGTATTTCTAGTTGGATGCACAATA
ACAAACCGAGCAAAAGTTGTGTAAGCACTAATCCCTTTCAAGAATAGACAATAATTTATAGAGGTATTTATT
GAAGTCAAAGCGATGATATATAATCCAACATTACTCTTTTGATCGCTCTCTCGATTCCCATCCTTTCAAGACC
ATCTTCTAACAGAATGCCGATCTTCCATACATCACTTACGTATCTTGCAATTATGGGTTGGCCCACGTTACAA
GGCGAACAAATCATGGGAACTCCTTTGCATACGCTCTCTAATGTTGAATTCCATCCACTGTGAGTCCAAAAA
CACCCTATCGCGGGATGTGCTAGAACTTCATTCTGCGGACACCATTTCACTATGTGCCCCTTACCAGCGACT
TTCTCTAGAAACTTCTCTGGCAACCACTCGAGCCATTCAGAATCTTCAACCAACCCATGTTGAACCACCCAC
AAGAAAGGAAAGCCAATGTTGGCTAACCCATGAGCCACTTCTTGAAACTCTGACTTCCTTATTTTTGCAAAG
CTCCCAAAACTTGCATATATTACAGACTTGGGTTTTTGAACATCTAGCCATGAGAAAAAAGTCCTATCTTCT
TCAAGCAGCTGGTTGCTTGTGAAACCTGGATGATACATGTGAAGTGGGCCTAAAGTGAATACTGGAACCCC
AAAATCTTGTCTAAGGTTTTCCAGTATAGGTTCTTCTGGTTGTACGAAGGAATTCCAAATTATTCCTGATGAT
GCTTTTATCTGTTTAGCAATATTCCTTTGAAGTTCTGCTGAACTTTTTGGATTAGTTGTAATCTTTACAATGTC
TTTGATTTTCAGAATTGGATATTTTGGCACCCGCGTTTCAAATTCTGAGTCTTTTACTGTAAGATTGAAGCAA
CCTTTATCATAGAAAGAAGGTAAATCCCTATAGACAAGGACCGAAACAATATTGCAAGCCCAAAACACCAA
CCGATGGACTTTCAAAGAGTTGGCCACATCCTGAGCAAAGTAGTATTCAGCGTCCGTGATCAAACAAGCCA
CCGGAGGCTCATTGGGTTCGGCCAATAATTCAGCTAAACAATCCCTAAATGAATCCATACAGTTGTTATTAA
GATAGGTAACGAAAAAGCTCGTGCTTGGTTTCTTCGGCATATACTTTTGAATATCCGAAAATCCATCATAAA
TCGACTTGAAAGTGAAGTGGGGATAGTTTGATTGATCAGGAGACAATGTTTGCGGATGTACGATTGTTATTT
TGAAACCTTGATTATGAAGAGTGTATGCTAGTTGAAATATAGGAGTAAGATGACCTTGAGCAGGCAATGGT
AACAATATTATTCTTTGGCCTATTCGCGACGATCTTGGAGTGATATGTTGATCTTCTTCCATATTTCAGTGAG
GTTTTAATTTAAAATAATTAAATTTTCATAGCTTTTTGTTGTAAGAACG

>GT_3_ TRINITY_DN44583_c0_g1 len=393
TGGGGGAATCTGCAGTATTGCCTCATGAACTTGAGGAGCTTATAAAAAAGAGAGGTTTTATAGGGAGCTGG
TGTTCGCAGGAAAAGGTTCTGAACCACCCTTCGGTTGGAGGGTTCATAACTCATGGTGGTTGGGGTTCAACA
ATTGAGAGCTTGTCAGCTGGGGTGCCGATGATATGTTGCCCATATGTGGCAGACCAAATGACTGATTGTAGG
TATATATGCAATGAATGGGGGGTTGGAATGGAGATGGAGAATAATATCAAAAGGGAAGGTGTTGAGAAGC
TTGTAAGGGAGTTGATGGGAAAAGGAGGTGAAAGGTTCAGGAGCAAAGCCATGGAGTGGAAAGAAAAGGC
CCGAATCGCCACAGGCCCTAATGGTTCGTCTTTCTTGA

>GT_4_ TRINITY_DN30626_c0_g1 len=1,749
GACTGTTTCACCTTTTTCACATTATTGTAACAATCAATTTTAATCTAAATAATACCCATATATCATTCTCTCTC
GCACTGCATACAACTAACCATTCTTGATTTCTTTCATGGAGAGCATAATGAAAGGTGCACATATACTAATAT
TCCCATTTCCAGCACAAGGTCATATGCTTCCACTACTAGACTTGACACACCACTTAGCCACCCATGGGCTAA
CCATAACCATCTTGGTCACACCCAAAAACCTACCAATTTTGAACCCACTTCTAGCTTCATGTCCCAACATCC
AACCACTGGTCTTCCCTTTTCCTGACCACCCTTCACTTCCAAAAGGTGTTGAAAATGTTAAAGATATAGGAA
ACCATGGAAACTTACATATAATCAACTCACTTGCCAAACTTCAAGAAACAATAATCCAGTGGTTCAACTCCC
ACCCTAGTCCTCCTGTTGCCATGATTTCAGATTTTTTTCTTGGATGGACTCAACACCTTGCTAACAAACTGGG
TATCCCCCGGGTCGGGTTTTTTTCATCTGGTGCTTTCTTGACTGCTGTTCTTGACTATGTTTGTCATAATATCA
AGTTGGTTCAGTCTCAAGATGTGACTGTTTTTCATGACTTGCCAAATGGGCCTGCTTTAAAATGGGAGGAGC
TTCCTACACTGGCCCGGGTGTATAAAGAATCTGACCCGGAATGGGAGTTGGTTCTTGATGGTCATATTGTTG
GGAGGTCTAGTTGGGGTTCGATTGTGAATACTTTTTATGGATTGGAGACTCAATATATGGAGTATTTGACCA
AAATAATGGGTCATGGGCGAGTTTTTGGAGTTGGTCCGGTTAGTTTGCTTAATGGGTCGGATCCAATGACCC
GTGGGAAATCGGAGTCCGGGTCCGATTATGATGTGCTGAGATGGCTTGATTCGAGGCCTGATGGGTCGGTTT
TGTATGTGTGTTTTGGAAGCCAAAAGTTTCTTAGTAAGGCCCAAATGGAGGCATTGGCTATTGGGCTTGAGG
AGAGTGGGGTCCATTATGTTTGGGTGGTGAAACAAGAGTTAGGTGACCCGGTTGTTTCGGGATCGGGTAGA
GGAATGGTGATAAATGGTTGGGCCCCACAGGTGTCGATATTGGGTCATCAAGCAGTGGCTGGATTTTTGAGT
CATTGTGGATGGAACTCGGTTTTGGAAGCTATTGTGGGTGGAGTGAAGATATTGGCATGGCCTATGGAGGCT
GACCAATACGTTAATGCTAGGTTGTTAGTAGACGACCATGGTGCGGCGGTGCGAGTTTGTGAAGGGGCGAA
TACCGTTCCCGACTCGGCCGAGTTGGCTCGTAAAATCGGCGAGTCAATGAGTGGGTCTGAGAATAATAAGA
AACTGAGAGCAAAAGAGCTTAAAGATAAAGCAATTGAAGCAGTCAAGGAAGGTGGGAGCTCATTTATGGA
ATTGGACAGGCTTGTCAACGAGTTATCCAACTTGGAACAAAAATTGGATGTTCCTCATTAGCATTTGATCTG
AAGAGCAAATTATGGTCATATTCTATTTAAAATTTAAAATATAAAATATATCTAGAAGCTCAGAAACTTTGA
TGTTCTATTTTCTTTGACGTTTTATTTGTTTTTAATGATAGTTATGGCTAAAACATGTAATGAGAATCGAAGT
GGTGATGGAAAAATTCAAAGCACACATTTCATTTAATCTCCCTCATAGCTATTTTAATTTCAGAAACTTATATA
GTTTTTTAAGTAGTTTTTA

>GT_5_ TRINITY_DN23238_c0_g1 len=2,126
GGGGCTTAAATACTCAAAGACCATCTTACAATATTAATATCCTAACAAAGAGGAAGATGATGGTAGAGTAT
TTCGAATAATGTTGTGAAAAATAAACAAACATATATTTTAATTAATGTATAAAACTTTTAACCCACAACTTA
TGATTATACTGGGTTGTTGTTTATGGCCTTTGAAGCAACATACGGGTAAACGAATATTAGTCGTCTCAGATA
TACAGGCTTAACCGGGTTATCCTGGGGCCGTATCCAAACCCCTCCCTAGCCAATATTAGTCGTCTGTCAATA
TTGTAACAACATCAGCAACAAGAGAAGGGAGCATCAAGAAATTGTAATGGAAGATGTAACGATATCATCA
ACGAAACTAACAAACTTGTCCAACTTAAAATATAACAAATTTCAAAAAATGACTGCAAGAACGCTTCAGAT
CAATCTTGATATAGAGGCTAACCGAGTAAATAGCCGAAAAAGTACTATCAGGGAATGAAGAGCAGAATATT
AGCATATTGTGTTAGCTAGGACCCTGGACAAGTTCAATGATATGCTACAAATAGATAGAAATTAATGAACTT

GAAATTGGGAAGCCATTGTTACTTTGGACTTAAAGATCTTAAATAGTAAAGTAGAAAATTGCAATAAAAAA
AAGTAAGAGAAACTAGTT
TCTTGACAATATGGTGATTTCACGAGCAAGATTTTCAATATTTAAGTAAGACGAGCCCTTAAGACCTGCAGC
AATTTCCGCAGTTTCCTTCCATTCCATAGCCTTCTTTCTCATCCGCTTACCTTCTATTCCATCCATTAACTCCC
TTACAAGTTTCTCGACTTCATCCCTCTTCACAGTACCCTCAATCTCCATTCCGACCTCCCATTCCTTACACATT
TGTCTACAATTGACCTGCTGATCATGAGAAACTGGCCAGCAAAGCATTGGTACCCCAGCAGACAAGCTCTC
AATCACTGAACCCCACCCACCATGAGTCAAGAACCCACCAACTGCAGGGTGGTTCAAGACCTCTTCTTGTGA
ACACCAGCTTGCAATAAAGCCTCTCTTTCTAATCACCTCCTCGAGTTCTTGAGGCAAAAGGGAAGGCTTCCC
ATCAACCAAATCAGTCCGTATAATCCATAGAAAATAATGGTTGCTGTTGACTAGTCCCCAACCAAATTCTAT
CAAGTCTTGTAAAGACATTGTTGCCAAACTTCCAAAGTTGACATACACCACAGAATTCGGTTCCTTCAACTG
CAGCCACTGGACACACTCGGGCTCTTCTTTCCATAAACTATATCCATTTAAATTTGTGCTATTGGTTTCTTTTT
CTGTTATCTGATTCAAAAGTAACTGCAGAGGCCCAACCGTGTAGACATTAGAAAAAATGGCTTTAACCTCAT
TAACAAGACTAGCTTCTAAGCCGTCAAAAGTATGAATAATCATGTGTGGAACTAGATCAGCCCTAGCAGCC
GTTTCCACAAGCCAATTAAAGCTAGGATCCTCAGGTTTTGTGGCTAGCATAAGTTTGGGTAGATCCCTTAAA
CGGACTTTTTGCATACCTGGAATCCAATCTATCAAGGTGTCAAGATACCCATTGGTCAAATCACTTTCATCTT
TAAGCGGGACAAGTCCTTTGTCCAGTAGAACTTTTGGCAGGTAATATGCCATGAAGCCACAGGCAGCCATA
GTCCAGTAAAGGATGAGCGGTACCTTTAGCTTCTCGGCAGCGTAAATAGTGTTTGTGAAAGTCATGAAACC
ATCACAGATAATACAAGTAGGAGGAGTTTTAAGCCCGTCTACAAGGTCAATAAAAGAATCAAAGAAATAGT
TTAAAAGGTGCAAAGTAATCTCTTCTGGGGTTTGAGGTGGTTCAGTTTCGTCAGCACAAGTATCAGGGAGAC
CGTCTGGAACCGTTTTGAACTGGAAACCAGGAAGGCCAGTGAGACCGTGAGAGCCCCCTTGCTTAAGAAGG
CGTTTCTGGTTCGACTTGGTATTGATAAAAGTTATGTCAAGGCCTTTGTGGTGTAGGACTCTGGCTAGTTTCA
GCATACACTTGATGTGGCTTTGGGCCGGAAATGGTATAACGATCACATGAGGCTTCTTCTGATCATCAACTT
TGGACACTACTGCTTCCATTTCTT

>CAT_1_ TRINITY_DN41650_c0_g1 len=2,751
GGAAGGTGAACATGTGCAGACTCTCGGGGTGGTGGGAGAAGAAGTCGAGGATTCTCCAGTCTTCTTGGATG
TGTGACTTGGGGTTGGGTTTCAAAGCATGAACCATGTCAGGAAACTTCATCCCATCACGAATGAAGAACAC
AGGGAAATTGTTTCCCACCAAATCAAAGTTTCCCTCTCTGGTATAGAACTTCACTGCAAAACCTCTAGGGTC
CCTGAGAGTTTCAGGGCTTCCACGCTCGTGAATAACAGTAGAGAAACGAACAATGACAGGAGTTTGGACTC
CAGGGGCACGAAGGAAATCGGCACATGTAAGTCCAGAAATGTCATGTGTGACCTCGAAGAATCCCTTAGCA
CTAGCACCCCTGGCATGGACAACACGTTCTGGGATACGCTCACGATCAAAGTTAGCTAGTTTCTCCACCAAA
TGGTAATCCTCGAGAAGGATTGGACCTCTAGAGCCAACTGTTAATGAGCTATTGTTGTTGTAAACTGGAGCA
CCAGAATTGGTAGTCCAGAATGGGGCGTTGTAGGCACTTGAAGGACGGTACTGGTTTTGGATATATCTTTGA
GGGTCTTGAAATGAAACTTGCATAGCATTACTGGACTTCATTTGGGTAAGAATTTTTGTTGACCGGATCTGC
ATGTAAAACTTTGGGCTTATAGCATCTTGGGCCATTTTATTGGCCCAAGAAAACCCAAGAAACGTCTATCAG
TTTTGACTTTTGAATAACTTGGAACACATTGAAGAAATTGGAATGGATTTCGTACCATAAGTTCATGGAGTT
GATCAAGGAGTCAAAGTCTTGCATTAATCCAGAGATTGACAAATCAGTAGCAGAAGCTTAAACCGTTGAGT
GTCATGCCAAGTGGACCAATGACAAAATGTCGCGTCGTTTTAATAACAGTTACTGTAAAAGTTGTATAAACG
AGTATACAAAGTGACGTTGCTAACTTTCATGATGTGTCAATGAGCAGATCCCGTTTCAAGTCCAAATACATA
TGCATACATCTTTAATCTTTCTCTTGACGGTAATCATTGGGCTTTTCACTAATTGGGCCCATGACAAATCGCT
GTTATTCATTGGGCTTTTCACTAATTAGGCCCATAACAAAATGCTGTCATTCATTGGGCTTTTCTCTTGACGG
TAATCAATGTGTTGAAAGTATGATACGTACACCATGCTACTTGTAATGAACCGTTTTGATAGTTGTTATAAT
ACAAAGTCGTCGGGGTAACCTTTACCGGAAAAAAAAAAGGAAAAGAAAAAACAAAACCACCATGGATTTA
AGCCCACAAAGAGCCACTGGCCAAAGATTGTTTCTCTGAACTTGGGCTCAGTAAAAGTTCAAACACATGAA
ATTCTTGCACAAAGCATCAATTAATGGCCAGAAACAACATTACATGTAAAAAGAAACTTACATAATACATG
TGTTCCACAAAGTTTAATCTTTCTCTTTACAGACTCGAACCTGAAACGCAGTAACTCAACACATCGTCGTGT
GCACTTATTTAAGTTACAGGTTCCTTAAGTAAGGGAAAATATTACAGAACGATGATAGATAGAACTCAAAC
ATTGACACCAGTAGTACAAACATAGAGACAGACCTTTTCTATAC
TAGCATGTTGACCATACTCGCTATGAAATGGAGTCCCGATATATTTATCTTCAATAGTTGGGGCGTACATTG
AGGCGTGATGCTATCTTCTGGCCCAAAGACTGGTCACACTGGGTCCAGTATGAGATCCAAATACTTCGGAGC
TCATGGGTGACCCGTGGGTCAGACAACATCTTAACCATTCGGACAATGAATCGTTCTTGCCTATCTGGTGCC
CATGATCTGTATCTCTCTCCGGGCTGCTTGAAATCATTAGCTTTTGGGATGACAGTCCTATCGCGGGTTCCAG
TGACCCTAGCAGGATTGATCGGGTACTGCTGAGCATGTTGAACACGGTCGTACCTTGAAGGGAAGTAATCC
ACCTCCTCATCTCTATGCATGAAATTCATATTTCCATCATAATGGTTGTTATGATGGGCACATTTAGGCGCAT
TCACTGGGAGCTGCAAGTAGTTTGGCCCGAGACGATGCCTCTGAGTATCAGAATACGCAAAGATCCTACCTT
GAAGCATTTTATCATCAGAGTAATAAATTCCAGGGACAACAAGACCAGGGTTAAAAGCCAACTGTTCATTC
TCAGCAAAGAAGTTATCGATGTTCTTGTTCAAGACCATACGACCCACTGGCTGCAATGGGATGATGTCCTCG
GGCCAAGTCATGGTGCCATCAAGCGGGTCAAAGTCAAGTCTGTCTTCATGATCAGGGTCAATAACTTGCAG
ATATAACTTCCATTCAGGGAAGTTACCAGCTTGGATCGAGTCATAAAGATCTTGAGTGGCATGACTATGATT
AGCACCTCCAATCTTGATAGCTTCCTCATCAGTCAAACATTTGACTCCACAAGTAGGCTTCCATGTGAACTT
CACATAATGCATTTTTCCCTCTTTGTTAACCAAAGTGAGAGTGTTAACACTCGAACCCTCCATGTGTCGGTA
ATCTGTTGGAATACCAACATCATCAAGCCAAAAAGTCATAGTGTTCAAACTTTCAGGATGGTGTGACAAAA
AGTCAAGAATTCTCCAGTCTTCTTGGATATGTGACTTTGGGTTGGGTTTAAACGCGTGAATCACATCTGGAA
ACGCCATTGCATCACGAGTAAAGAACACTGGGAAATTGTTACCCACAATGTCA

>CAT_2_ TRINITY_DN32938_c3_g1 len=590
TCCGATCCACAAGCTTAAACAACCTTCAATTCTCTCTTCTTTCTTTTGTTTTATTTTTCATTCCCAACATCATG
GATCCCTACAAGGGTGCGAAACTCATCCTCCCTCACCCTGATCAACGCCCTTCAAGCGCTTACAACTCTCCG
TTCTGGACAACTAATGCTGGTTTACCTGTTTACAACAATACTGCATCATTGACTGTTGGAAACAGAGGTCCA

ATTCTCCTTGAGGATTATCATTTGATTGAAAAGCTTGCAAACTTCACCCGTGAGCGGATCCCAGAACGTATT
GTTCATGCAAGGGGAGCGAGTGCAAAGGGATTCTTTGAAGTCACACATGACATTACTCATTTGACATGTGCT
GATTTCCTTCGTGCACCAGGAGTCCAGACTCCTACTATTGTTCGTTTCTCTACTGTTATCCATGAACGCGGAA
GCCCTGAAACAATCAGAGACCCTCGGGGTTTTGCAACAAAGTTTTACACCAGAGAGGGTAACTTTGACATT
GTGGGTAACAATTTCCCAGTGTTCTTTACTCGTGATGCAATGGCGTTTCCAGATGTGATTCACGCGTTTAAAC
CCAACCCAAAG

>POD_1_ TRINITY_DN35984_c0_g1 len=1,249
GCCCATCTTGTTTACATACATTCAATACCAATATAACAAACCTCGTTAAGAAAAAAGGGAAAAACTAGCTA
TCAAGCAGTATAAGTTGTTCAAACCGAAATTTTTTGATCAAGAGTGCAAAACGAAATTGAAATCAGTTGATT
TTGCGACAGTTTTGACGAACTTCTCCAGTCTCTTTAACATCAAGCAACCCCATCTTCACCATGGCTCGTTGGA
AATCAAAGAAGAACATGGCTTGGTTCATGGAGTACGCGTTGACAATCGGTCTGGTCTTTGGGTCAGTCATCA
AGGTTTGGTCCGATGATAGGAGCCCTGCCTGCCTTTGAAGCGCATAGTAGTAATTATTATCAAACATGTTTC
TTGTCGTGTCGAAGGGCTGTTCCGCATTGTCGCCTGCATTACATGTTTTAGCTAGCGTTTTCACAAATTGAGC
GTCGATGCTTGGGTCCACTTCGTGCGTTGAATCAAATCCTTTTAGCCGGTTCTTGAATGATGAACATCGAGC
CACTCCTAGTGTATGTCCGCCAGAAAGAGCAACCATTTCTTGAGCAGTGAAACCATGTTGGCCAAACATTTG
AATGAGTTCACTACTGTTAAAAGTTGGTGGAGGCAGGTTTCTTGTGTCTTCTATTTTCGATCGTGTTCCATCT
TTTCTTCCTTTTGGTATATCATAAACTGGACCCCCGGCAAAGAAAACAGCGTCACGGGCAGCCATTGCCACA
ATATCGGCACAAGATACTACACCGGGACATTGGTCTTCAAGTAGTTTCTTGGCGTCGTCGATTACTTTATAG
CCTCTTAAACTCAAATTAGCAGGCGAGTCTTTCTCTGCAGTGTTGTCACTAGTAGAATCAAGCAAAATTGAC
GCGTCACAACCCTGAATGAAGCAGTCGTGAAAATGCATCCTTATAAGACCAGCCGCAAGAGTTGGATCAGC
TCTCAAAGCTGAGTTAACCGAGTTCTTAACAATGAACTCGGCCATAGGGCAACTCATCAAGTAGTAATTCAT
GTTGAGACCGTTGCATTGTCTCAACATAACTATCATTACCATCACCACAATCATCATCATTTTTGTGACTTTA
AATATGATGGTAGTCGATGAACTTAGCGACCTTTGCTTATATATATCAAAGTGGAAAACGTGTTGCTATAAG
GGCAAAATTTGTCACTAAATATGTAGTAATAAAACTTGTTGGTGGCAAGTAGTGCAAGAATGACAAAAAGC
AATACAAACAAGCTGGCTTTTGAT

>POD_2_ TRINITY_DN34083_c0_g1 len=1,334
GTACAAACCTTATCCCCTACTTTCACCTATACATTGAACCGCAAGTGATATATAAAACATACTTCTCATTTCA
TTTCATTCCTTCATTATAAAGGTAAAACCACACTTAAAAACCACACCCCCATAAACATTTTTATTTTTCTTTC
TAAACAATGTATCTCCCTTCTTTTTGTCTCCCTATAATCTTCTTCTATCAACTTACGGCAATCATCACAGCATC
ATCACCACTCCGGCCCGGATATTATGCCGAAACTTGCCCGAATGCAGAGTTCATTGTTAGAGATGTTATGAG
AAAAGCCATGATCAGAGAACCAAGAAGTGGTGCTTCTGTCATGAGGTTTCAGTTTCATGATTGTTTTGTTAA
CGGATGTGATGGTTCAATGTTGTTGGATGATACTCCAACCATGCTGGGAGAGAAGCTATCTTTGTCCAACAT
AGATTCGTTGAGATTGTATGAGGTTGTCGATGAAATCAAGGAAGCACTCGAGAAGGCCTGCCCTATGACTG
TTTCTTGCGCGGATATCATTATCATGGCATCTAGAGACGCTGTTGCACTGAGTGGAGGTCCGGACTGGGAAG
TAAAATTAGGAAGAAAAGACAGTCTTTTCGCGAGCCAAAAGGATGCAGATGACATAATGCCGAGTCCAAG
AGCTAATGCAAGTATGCTCATCGATCTTTTTGCTAAATACGACCTCACGGTCAAAGATTTAGTAGCTCTTTC
GGGTTCTCATTCAATTGGCAAGGGACGTTGTTTCTCCATTGTGTTTAGGCTATACAACCAATCAGGCACCGG
CCGTCCTGACCCCACCATCGAGCCTAACTATAGAAAAATGTTAGACAAACTTTGCCCCCTTGGTGGTGATGG
TAATGTGACCGGCGATTTGGACTTCACGCCTCGTGTGTTTGACAACCAATATTATAAAAATTTGGTGGATGG
AAGAGGTTTTTTGAATTCTGATGAAACTTTGTTTACTTATCCGAAAACTAGAAAATATGTTCAAAGGTATAG
TGAAGATGAAGCCGAATTTTTTAGGGATTTTGTGGATGGGATGATAAAAATGGGGGACTTACAATCAGGAC
GACCCGGGGAGATTAGGAAGAATTGTAGGGTGGTTAATGGCCAGCCACCAGTTAGCGTGGTGGTCGGCGAG
GAATGGGAAGATAGAAAAAGATCGACCGGGTCTCTAAGGGTAAACTTTACCATAGAATGATTATTATTACC
ATGAGAATGGATAATTTGTAGCATCTGTTTTCATCTTATAATTTAAACGATTTTCTGAAGTTCTTTATACGAA
GATTAATTATCATTCATTAAGATCTATAGAGCGAACGAC

>SOD_1_ TRINITY_DN36049_c1_g1 len=1,320
GAAGAATCCTAAAAATCTCTACTTAAAAAAGGTCCTCCTTTTACACTCCATTCTACATTTCAAGCAAATCCA
ACAAAAGCTCTTTTCAAGAAACACCCACAATCACTTTAACCTATTACACCTTACCCAACACAAGCAGAACCA
TAGTCAACAAAGCTATATAGTTGTGTTTCTGTCAAGGTGCTAGAAAGAGGTGGTCAGTAGATATGAGTTGGT
CATGTAGTAACTATCTTTGCATATCAACTTCAAGTTCTAGCTCTGTTATAAGTTCTAATGAGCTTACAAGGTC
TAATAGCTGCAAGTTGGTTTCTAAGTATAACGAGCAAAGAAAAAAAAGGTCATGACTTCAAAACCCCAACA
AGTATTATTGCTTACAGTGGTTTGAGACCAACATCTTTTAGGCTTGATAAAGGTCAAATAAGGAAAAGTCAT
GTCTTGAATGAGCCAAACAACATCTGTGCTTATTATAGCTTGAAACCTCCATCTTATAATTTCGATGCTCTGG
AGCCATATATGAGTCAAAGGACTCTTGAGGTGCACTGGGGAGAGCATCACCGTGGTTATGTTGAAAGGTTG
AATACGTTCTTAAAGAAAGACGATATAATGTATGGATACACATTGGATGAATTGATCAGAATAACATACAA
CAATGGAAATCCATTACCAGAATTTAATGTTGCTGCTCAGGTCTGGAATCATGATTTCTTCTGGGAATCCAT
GCAACCAGGAGGTGGTAACTTACCGATATTGGGTCTCCTTCAGCAGATTGAAAAGGATTTCGGCTCCTTCTC
AAATTTCAGAGAAGAGTTCATCGAAACTGCCGTCTCATTATTTGGCTCTGGCTGGGTTTGGCTTGTTTTAAA
GCGGAATGAGAGACGACTCGCAATAGCTACAACAACGAATGCTATAACTTCTATTGTTTGGGACGATATTC
CTCTTCTTAGTTTGGATATGTGGGAGCATGCTTATTATCTGGATTACAAGAATGACAAAGCCCGGTATGCCA
ACACTTTCATGGACCATCTTGTGTCTTGGAATGCGGCTTTGGCTCGCATGGCTCGTGCACAGGCTTTTGTAAA
TTTAGGAGAACCCAAGATTCCAGTTGCTTAGGATTTTATTTCTTTCTCTCCTTTTTAGATAGCAGATTTTATC
AGGATTAGTGCGACTTCAATTTAGAAAATGTCTGTTTGAGTATTTAAGTTTTGAGGCTGCTGTAGTTTGTACT
TTCTAGTGTACTCGAGTAATGTTGTTTGGGCTTATATTTTTCATTTGTTTCTTTCAAACTATTGGAGTATTGGA
CTATTCTTCATTCACTTTGGC

>SOD_2_ TRINITY_DN32389_c0_g1 len=1,006
CTTCTCTTTAGTATAATTGGTAACGGATTATTCGACGTTGCATATGAGTTGATCAAGAAGTACCCTGACATG
GCTAGAACAAAGAAAGACTTTTTCTTTCCATTGAAGGCTATCGCTGGAAAGTCAGGCGCCAGAAGAACATA
CAACTTCTACCAACAATTTGTTTATCATTATGTGCCATTACAAGACCAGGACCACAACCAGGACAATGCACA
CCAGATTGATCGCGATATCGAGAATCAAGTTCGAGATACCACTATACCAATTGTCAACGGCAGGAGAAGCT
GCTTCTACTCAGTCATCAGATGGATACTTGTCAAGTTATGGAAAGTTATACTACTACATGGAAGTTCCGGAA
GAAGGAACTAGTGATCTTGACTTGAAGCAATAGTTGGTTTTGAGGTGTTTTGCGAGCTTGGAAGGTATTTAG
GTCACTCTTGATGGAATGACTCGTTTTTGGATTAGAGGCCTAGTATTCCCTCTCTTCTTCGGCTCTTGGTACA
TTTGGTTATTTTGGTGCTTTGGGTTGAAATTGTATATGTCAAATGTAAGACTTTTGAATGTATGTAAAGTTGG
GCGAAAATGATGTCAACTTGGGTAAACGACCCATTTAGGGTGTTCCCAAGTCTTTTATCACTTTAAGGTCGG
TATGTTGATCGAATCTGGTTCATAATGTTGTTCGAAACTTTGTAATGGTATGTTTGAAGTTAAGTTCGAAAAT
GTAATGGTTATAAGTCCTTGAAGGTGTTGTTTAGGTTTTTGGCATTTCTGTGCAGGTGTTCCACTGCGGCGCA
GTGGGATATGGTTTTATCCACTGCGACGCAGTGGATCTATTCTGTTAATGTTTTTACGAAACTTCCCACTGCG
GCGCAGTGGGATGGCGTCAAGACCCACTGCGGCGCAGTGGGGGCTTTTTGGTCCGGAGCAGAGGATTTCCA
CTGCGGCGCAGTGGGGAGTTCTCGACCCACTGCGGCGCAGTGGGATCCTTCTGTAAAAAAAAAAAAAGA

>EPSPS_1_GR len=1,830
CTGGAATGGCAAAATATTATATTACACTAAAAAAATCTTACCAAAGAATACAACTTCATAAAAATAGTCCC
AAAACTCACATATTATACACGTTACAAATGAAACAAAAATTACATATAAAAGAGCAAACCACTACCCATAA
TCACATTAATGCTTGGCAAATCTTTGAAGAACTTCAAAGTAATCAGGAAACGTCTTACGAGTGCAAGAAGG
GTCCTTAATGGTCACAGGAACATCTGCACAAGCAGCAAGAGAGAAAGCCATGGCCATCCTATGATCATCAT
ATGTGTCTATTGCTGTCACGTTTAACTTCTCTGGCGGAGTGATCACACAATAGTCTGGACCTTCTTCAACTGT
TGCTCCCAACTTTCTAAGTTCTGTGCAAATGGCAATCATCCTTTCGGTTTCTTTAACTCTCCAGCTAGCAACA
TCTCTAATGGCTGTAGGGCCATCAGCATAAAGAGCGACCACAGCAAGAGTCATGGCAACATCAGGCATCTT
GTTCATGTTCACATCAACAGCACGTAAATGTTTCCTTCCAGAAGAATCCCTTGGTGGGCCCTTAACTGTGAC
TGAGTTCTCAGTCCATGTTACTTCCGCACCCATTTGTCCAAGTACCTCCGCAAACTTCACATCACCCTGCAAA
CTACTTGTTCCGCAGCCTTCAACGGTGATGGTTCCTCCAGTTATGGCAGCACCAGCCAAGAAGTAACTCGCA
CTTGATGCATCACCTTCTACATAAGCATTTCCAGGTGACTTGTACTTTTGGCCGCCTCGAATAAAGAACTGG
TCCCAACTATCACTATGTTCTACCGAGACGCCGAACCGTTCCATTAATTTCAGTGTCATCTCGACATATGGTA
TGGAAATTAGTTTATCTATGATTTCAATTTCCACGTCCCCAAGGGCAAGGGGAGAAGCCATAAGCAGCGCA
GTAAGGTATTGACTACTAATAGATCCCGACAACTTAACCTTTCCTCCAGGGAGACCACCTCCACCAACTACA
CGCACAGGAGGGCAGTTCGTCCCGAGAGAACAGTCAACATCTGCCCCAAGCTGCTTAAGACCCGTGACCAA
ATCACCTATTGGTCTCTCTCTCATACGAGGAACGCCATCTAGTACGTAGCTTGAATTACCACCAGCAGCAGT
AACTGCGGCAGTCAATGGACGCATAGCAGTTCCTGCATTCCCAAGAAAAAGCTGTATGTCATCTTTAGCTTC
TTTACCCACAGGAAACATACCGCCACAACCTTCCACAATTGCCCTTTTAATTGCACCATCCTCCTCAACGTTT
AGCCCTAGAGTTCTTAAAGCTCCAAGCATGTAATGAACATCATCACTGTTGAGTAAGTTGTCAACAATGGTC
GTTCCCTCGGCGAGCGCAGCAAGGAGGAGGATCCGATTAGACAAAGACTTGGACCCGGGTAAATTAACCGT
ACCCGATATTTCTTTAATGGGTTGCAACACAATTTCCGGCACCGTTGATGGTTTCTCGGTGGTGGCAACGGC
AGAAACAATTAAACATTTTTTTCCAAGATTTCTGTTATTATTAGAAGAAACAGAGAAATGGGCAATTGGGTT
TGTGTGTTTTGGCACAAAAGATAAAAAAGGTTGTGACTTTATTGATGGGGTTTGGGTTTTGGAAAGACTGGT
GGTAGCTTGGAGATTATGGGCAATGTTGGTGGTGTTAATGTGAGTAGCTGCCATTGTTTGGTGTGTGTGAAA
TTTCACACAAAACACACAGTGGGTTTGAAGGATTTAATGATTTGGATTTGGGTTGGGTTTGAAGGGAGGGGT
CAGTAGTAGGTGGGTGTTTGAAGAATTTTCATA

>EPSPS_1_GS len=1,918
TTTTTTTTTTACTAATAATTACTGTAATAGAAATCAACTGGAATGGCAAAATATTATATTACACTAAAAAAA
TCTTACCAAAGAATACAACTTCATAAAAATAGTCCCAAAACTCACATATTATACACGTTACAAATGAAACA
AAAATTACATATAAAAGAGCAAACCACTACCCATAATCACATTAATGCTTGGCAAATCTTTGAAGAACTTC
AAAGTAATCAGGAAACGTCTTACGAGTGCAAGAAGGGTCCTTAATGGTCACAGGAACATCTGCACAAGCAG
CAAGAGAGAAAGCCATGGCCATCCTATGATCATCATATGTGTCTATTGCTGTCACGTTTAACTTCTCTGGCG
GAGTGATCACACAATAGTCTGGACCTTCTTCAACTGTTGCTCCCAACTTTCTAAGTTCTGTGCAAATGGCAA
TCATCCTTTCGGTTTCTTTAACTCTCCAGCTAGCAACATCTCTAATGGCTGTAGGGCCATCAGCATAAAGAG
CGACCACAGCAAGAGTCATGGCAACATCAGGCATCTTGTTCATGTTCACATCAACAGCACGTAAATGTTTCC
TTCCAGAAGAATCCCTTGGTGGGCCCTTAACTGTGACTGAGTTCTCAGTCCATGTTACTTCCGCACCCATTTG
TCCAAGTACCTCCGCAAATTTCACATCACCCTGCAAACTACTTGTTCCGCAGCCTTCAACGGTGATGGTTCCT
CCAGTTATGGCAGCACCAGCCAAGAAGTAACTCGCACTTGATGCATCACCTTCTACATAAGCATTTCCAGGT
GACTTGTACTTTTGGCCGCCTCGAATAAAGAAGCGGTCCCAACTATCACTATGTTCTACCGAGACGCCGAAC
CATTCCATTAATTTCAGAGTCATCTCGACATATGGTATGGAAATTAGTTTATCTATGATTTCAATCTCCACAT
CCCCAAGGGCAAGGGGAGAAGCCATAAGCAGCGCAGTAAGGTATTGACTACTAATAGATCCCGACAACTTA
ACCTTTCCTCCAGGGAGACCACCTCCTCCAACTACACGTACGGGTGGGCAGTTCGTCCCAAGAGAACAGTC
AACATCTGCCCCAAGCTGCTTAAGACCCGTGACCAAATCACCTATTGGTCTCTCTCTCATACGAGGAACGCC
ATCTAGTACGTAGCTTGAATTACCACCAGCAGCAGTAACTGCGGCAGTCAATGGACGCATAGCAGTTCCTG
CATTCCCAAGAAAAAGCTGTATGTCATCTTTAGCTTCTTTACCCACAGGAAACATACCGCCACAACCTTCCA
CAATTGCCCTTTTAATTGCACCATCCTCCTCAACGTTTAGCCCTAGAGTTCTTAAAGCTCCAAGCATGTAATG
AACATCATCACTGTTGAGTAAGTTGTCAACAATGGTCGTTCCCTCGGCGAGCGCAGCAAGGAGGAGGATCC
GATTAGACAAAGACTTGGACCCGGGTAAATTAACCGTACCCGATATTTCTTTAATGGGTTGCAACACAATTT
CCGGCACCGTTGATGGTTTCTCGGTGGTGGCAACGGCAGAAACAATTAAACATTTTTTTCCAAGATTTCTAT
TATTATCAGAAGAAACAGAGAAATGGGCAATTGGGTTTTTGTGTTTTGGCCCAAAAGATAAAAAAGGTTGT
GACTTTATTGATGGGGTTTGGGTTTTGGAAAGACTGGTGGTAGCTTGGAGATTGTGGGCAATGTTGGTGGTG

TTAATGTGAGTAGCTGCCATTGTTTGGTGTGTGTGAAATTTCACACAAAACACACAGTGGGTTTGAAGGATT
TAATGATTTGGATTTTGGGTTGGGTTTGAAGGGAGGGGTCAGTAGTAGGTGGATGTTTGAAGAAGGTCAAA
AACAAAATTTCATACCAACCACCACCACCACCACTTCCTACCAAACCTCCA

>EPSPS_2_GR len=662
AAGAAGTAACTCGCACTTGAAGCATCACCTTCCACATAAGCATTTCCAGGTGACTTGTACTTTTGACCGCCT
CGGACATGGAATCTGTCCCAAGTATCACTGTGTTCTACTGAAACCCCAAACCGCTCCATCAACTTAAGTGTC
ATCTCCACATATGGTACAGAGATCAATTTATCTATAATTTCTATCTCTACGTCTCCCAGTGCAAGAGGAGCC
GCCATAAGCAAAGAAGTCAAGTATTGGCTACTTATAGATCCTGACAATTTCACCTTTCCACCAGGAAGGCCT
CCACTTCCAACTACACGAACCGGTGGGCAGTTTGTACCGAGAGAACAATCAACATTTGCACCAAGCTGTTTT
AGACCGGTGACCAGATCACCAATTGGCCTCTCCTTCATTCGAGGAACACCATCTAGTATGTAGCTTGAGTTT
CCACCGGCAGCAGTAACGGCAGCAGTCAATGGACGCATAGCTGTTCCTGCATTTCCAAGAAAAAGCTGGAT
TTCATCCTTGGCTTCTTTACCCACAGGAAATACACCACCACAACCTTCTACGATTGCTCTTTTAATTGCACTA
TTTTCTTCAACATTTAACCCTAGAGCTCTTAAAGCTCCAAGCATGTAATGAACATCATCACTGTTTAATAAGT
TGTCTACAACAGT

>EPSPS_2_GS len=1,445
CAAAAACACTTTTCATTATAATACAAAAAATACATTATGCATAAAAAGAATACATTCCAATTTAGCACAAA
GATTCGAGATGATACTTATTCCACCTTTAAGAAACTACAAACTCAACTAATAGAAAAAGATTAATTATACGC
ACGGGTCGTCCATATATATGTGTGGTTTAAGTGAAACCCTTCTGAAGAAAGATAACGATGAAGGGTAAAGG
CGTCATTTTAAACACGCTAAACCCTTTCTTTTTTATCATAAAAGATTTGATTTAATGCTTAGTATATCTTTCA
AGAACTTCAAAGTAGTCGGGGAAGGTCTTACGGGTGCAACCTGGGTCCTTAATGGTGACAGGAACCTCTGC
ACAGGCGGCAAGCGAGAAAGCCATGGCCATTCTGTGATCATCATATGTGTCAATCGCTGTCACATTCAATTT
CTCTGGTGGAGTGATCACACAATAATCTGGACCTTCTTCGACTGTTGCTCCCAACTTTCTTAGTTCAGTGCAA
ATGGCAATCATCCTTTCTGTTTCTTTTACTCTCCAACTAGCCACGTCTCTGATGGCAGTGGGGCCATCAGCAT
AAAGGGCAACCACAGCAAGAGTCATCGCAACATCTGGCATTTTGTTCATGTTCACATCTACTGGACGCAAGT
GTCCCCTTCCGGAAGAATTCCTTGGTGGACCCTTCACTGTGACAGAGTTCTCTGTCCAGGTTACTTCAGCACC
CATTTGTCCAAGGACCTCAGCAAATTTTACATCACCCTGTAAACTGCTTGTCCCGCAACCTTCCACGGTGAC
AGTTCCGCCAGTGATGGCAGCACCAGCTAAGAAGTAACTCGCACTTGAAGCATCACCTTCCACATAAGCAT
TTCCAGGTGACTTGTACTTTTGACCGCCTCGGACATGGAATCTGTCCCAAGTATCACTGTGTTCTACTGAAAC
CCCAAACCGCTCCATCAACTTAAGTGTCATCTCCACATATGGTACAGAGATCAATTTATCTATAATTTCTATC
TCTACGTCTCCCAGTGCAAGAGGAGCCGCCATAAGCAAAGAAGTCAAGTATTGGCTACTTATAGATCCTGA
CAATTTCACCTTTCCACCAGGAAGGCCTCCACTTCCAACTACACGAACCGGTGGGCAGTTTGTACCGAGAGA
ACAATCAACATTTGCACCAAGCTGTTTTAGACCGGTGACCAGATCACCAATTGGCCTCTCTCTCATTCGAGG
AACACCATCTAGTATGTAGCTTGAGTTTCCACCGGCAGCAGTAACGGCAGCAGTCAATGGACGCATAGCTG
TTCCTGCATTTCCAAGAAAAAGCTGGATTTCATCCTTGGCTTCTTTACCCACGGGAAATACACCACCACAAC
CTTCTACGATTGCTCTTTTAATTGCACTATTTTCTTCAACATTTAACCCTAGAACTCTTAAAGCTCCAAGCAT
GTAA

>EPSPS_3_GR len=1,830
CTGGAATGGCAAAATATTATATTACACTAAAAAAATCTTACCAAAGAATACAACTTCATAAAAATAGTCCC
AAAACTCACATATTATACACGTTACAAATGAAACAAAAATTACATATAAAAGAGCAAACCACTACCCATAA
TCACATTAATGCTTGGCAAATCTTTGAAGAACTTCAAAGTAATCAGGAAACGTCTTACGAGTGCAAGAAGG
GTCCTTAATGGTCACAGGAACATCTGCACAAGCAGCAAGAGAGAAAGCCATGGCCATCCTATGATCATCAT
ATGTGTCTATTGCTGTCACGTTTAACTTCTCTGGCGGAGTGATCACACAATAGTCTGGACCTTCTTCAACTGT
TGCTCCCAACTTTCTAAGTTCTGTGCAAATGGCAATCATCCTTTCGGTTTCTTTAACTCTCCAGCTAGCAACA
TCTCTAATGGCTGTAGGGCCATCAGCATAAAGAGCGACCACAGCAAGAGTCATGGCAACATCAGGCATCTT
GTTCATGTTCACAT
CAACAGCACGTAAATGTTTCCTTCCAGAAGAATCCCTTGGTGGGCCCTTAACTGTGACTGAGTTCTCAGTCC
ATGTTACTTCCGCACCCATTTGTCCAAGTACCTCCGCAAACTTCACATCACCCTGCAAACTACTTGTTCCGCA
GCCTTCAACGGTGATGGTTCCTCCAGTTATGGCAGCACCAGCCAAGAAGTAACTCGCACTTGATGCATCACC
TTCTACATAAGCATTTCCAGGTGACTTGTACTTTTGGCCGCCTCGAATAAAGAACTGGTCCCAACTATCACT
ATGTTCTACCGAGACGCCGAACCGTTCCATTAATTTCAGTGTCATCTCGACATATGGTATGGAAATTAGTTT
ATCTATGATTTCAATTTCCACGTCCCCAAGGGCAAGGGGAGAAGCCATAAGCAGCGCAGTAAGGTATTGAC
TACTAATAGATCCCGACAACTTAACCTTTCCTCCAGGGAGACCACCTCCACCAACTACACGCACAGGAGGG
CAGTTCGTCCCGAGAGAACAGTCAACATCTGCCCCAAGCTGCTTAAGACCCGTGACCAAATCACCTATTGGT
CTCTCTCTCATACGAGGAACGCCATCTAGTACGTAGCTTGAATTACCACCAGCAGCAGTAACTGCGGCAGTC
AATGGACGCATAGCAGTTCCTGCATTCCCAAGAAAAAGCTGTATGTCATCTTTAGCTTCTTTACCCACAGGA
AACATACCGCCACAACCTTCCACAATTGCCCTTTTAATTGCACCATCCTCCTCAACGTTTAGCCCTAGAGTTC
TTAAAGCTCCAAGCATGTAATGAACATCATCACTGTTGAGTAAGTTGTCAACAATGGTCGTTCCCTCGGCGA
GCGCAGCAAGGAGGAGGATCCGATTAGACAAAGACTTGGACCCGGGTAAATTAACCGTACCCGATATTTCT
TTAATGGGTTGCAACACAATTTCCGGCACCGTTGATGGTTTCTCGGTGGTGGCAACGGCAGAAACAATTAAA
CATTTTTTTCCAAGATTTCTGTTATTATTAGAAGAAACAGAGAAATGGGCAATTGGGTTTGTGTGTTTTGGCA
CAAAAGATAAAAAAGGTTGTGACTTTATTGATGGGGTTTGGGTTTTGGAAAGACTGGTGGTAGCTTGGAGA
TTATGGGCAATGTTGGTGGTGTTAATGTGAGTAGCTGCCATTGTTTGGTGTGTGTGAAATTTCACACAAAAC
ACACAGTGGGTTTGAAGGATTTAATGATTTGGATTTGGGTTGGGTTTGAAGGGAGGGGTCAGTAGTAGGTG
GGTGTTTGAAGAATTTTCATA

>EPSPS_3_GS len=1,786

AAAAAATACATTATGCATAAAAAATAATACATTGCAAACCAAATTTAGCATAAAGATTCGAGATGATACTT
ATTCCACCTTAAAAAACTACAAACTCAACTAATAGAAAAGATTAATTATAGGCTCGGTCTTCCATATATATG
TGTGGTTTAAGTGAAACCCTTCAGAAGAAAGATAACGGTGAAGAGTAAAGGCGTCATTTTAAACACGCTAA
ACCCTTTCTTTTTGAACATAGAAAATTTGATTTAATGCTTAGTGTATCTTTCAAGAACTTCAAAGTAGTCGGG
GAAGGTCTTACGGGTGCAACCTGGGTCCTTAATGGTGACAGGAACCTCTGCACAGGCGGCAAGCGAGAAAG
CCATGGCCATTCTGTGATCATCGTATGTGTCAATTGCTGTCACATTTAATTTCTCTGGTGGAGTGATCACACA
ATAATCTGGACCTTCTTCCACTGTTGCTCCCAACTTTCTTAGTTCAGTGCAAATGGCAATCATCCTTTCTGTTT
CTTTTACTCTCCAACTAGCCACGTCTCTGATGGCAGTGGGGCCATCAGCATAAAGGGCAACCACAGCAAGA
GTCATCGCAACATCTGGCATTTTGTTCATGTTCACATCTACTGGACGCAAGTGTCCCCTTCCGGAAGAATTCC
TTGGTGGACCCTTCACTGTGACAGAGTTCTCTGTCCAGGTTACTTCAGCACCCATTTGTCCAAGGACCTCAG
CAAATTTTACATCACCCTGTAAACTGCTTGTCCCGCAACCTTCCACGGTGACAGTTCCGCCAGTGATGGCAG
CACCAGCTAAGAAGTAACTCGCACTTGAAGCATCACCTTCCACATAATTCCAGGTGACTTGTACTTTTGACC
GCCTCGGACATAGAATCTGTCCCAAGTATCACTGTGTTCTACTGAAACCCCAAACCGTTCCATCAACTTCAG
TGTCATCTCTACATATGGTACAGAGATCAATTTATCTATAATTTCTATCTCTACGTCTCCCAGTGCAAGGGGA
GCTGCCATGAGCAAAGAAGTCAAGTATTGACTACTTATAGATCCTGACAATTTCACCTTTCCACCAGGAAGG
CCTCCACTTCCAACTACACGAACTGGTGGGCAGTTTGTACCTAGAGAACAGTCAACATTTGCACCAAGTTGT
TTTAGACCAGTGACCAGATCACCAATTGGCCTCTCTCTCATTCGAGGAACACCATCTAGTACGTAGCTTGAA
TTTCCACCAGCAGCAGTAACGGCAGCAGTCAATGGACGCATAGCTGTTCCTGCATTTCCAAGAAAAAGCTG
GATTTCATCCTTGGCTTCTTTACCCACAGGAAATACACCACCACAACCTTCTACGATTGCTCTTTCAATTGCA
CCATTTTCTTCAATATTTAACCCTAGAGCTCTTAGAGCTCCAAGCATGTAATGAACATCATCGCTGTTTAATA
AGTTGTCTACAACAGTAGTCCCCTCAGAAAGGGCAGCAAGAAGGAGGATCCGATTAGATAAAGACTTGGAT
CCGGGTAAATGAACCGTACCCGAAATTTCTTGAATGGGTTTCAACACAATTTCTTCTGGAGCTTTAGATGGC
TTCTCTTTGGTGGAAGTGGCAGAGAAAGCTGATACTTTAAATGGGTTTTTGTTGTTTTTCACATTACAAGAA
ACAGAGGTATAATTATTGTTATTATTGTTTTTTAATGGGTTTTTGAAGTTGGATCCAAAAGATAAAAAAAAA
AAATAAGAAAAGATGATGACTTTGAAGATGGCTTTTGGGTTATGAGATTGGAAGTG
